
JOURNAL INFORMATION
==============================
NLM Title Abbreviation: Insights Imaging
Journal ID: Insights Imaging
NLM Journal ID: 101532453

Insights into Imaging

EISSN: 1869-4101
Publisher: Springer

ARTICLE INFORMATION
==============================
PMCID: PMC6548780
PMID: 31165358
DOI: 10.1186/s13244-019-0748-0
Article ID: 748
Article version: 1
Subjects: Meeting Abstracts

ESGAR 2019 Book of Abstracts

Electronic publication date: 2019 Jun 4
Publication date: 2019 Jun
Volume: 10
Issue: Suppl 2
Issue sponsor: ESGAR 2019 is the annual meeting of the European Society of Gastrointestinal and Abdominal Radiology. Publication costs were not supported by sponsorship.
Electronic Location ID: 58
Copyright: © The Author(s). 2019
License: Open AccessThis article is distributed under the terms of the Creative Commons Attribution 4.0 International License (http://creativecommons.org/licenses/by/4.0/), which permits unrestricted use, distribution, and reproduction in any medium, provided you give appropriate credit to the original author(s) and the source, provide a link to the Creative Commons license, and indicate if changes were made.
License URL: https://creativecommons.org/licenses/by/4.0/

Conference name: The European Society of Gastrointestinal and Abdominal Radiology 30th Annual Meeting and Postgraduate Course
Conference Acronym: ESGAR 2019
Conference location: Rome, Italy
Conference date: June 5 - 8, 2019
issue-copyright-statement: © The Author(s) 2019

==============================
European Society
ESGAR
Gastrointestinal and Abdominal Radiology

### Insights into Imaging

Education and strategies in European radiology

ESGAR 2019 Book of Abstracts / Volume 10 / Supplement 2 / June 2019

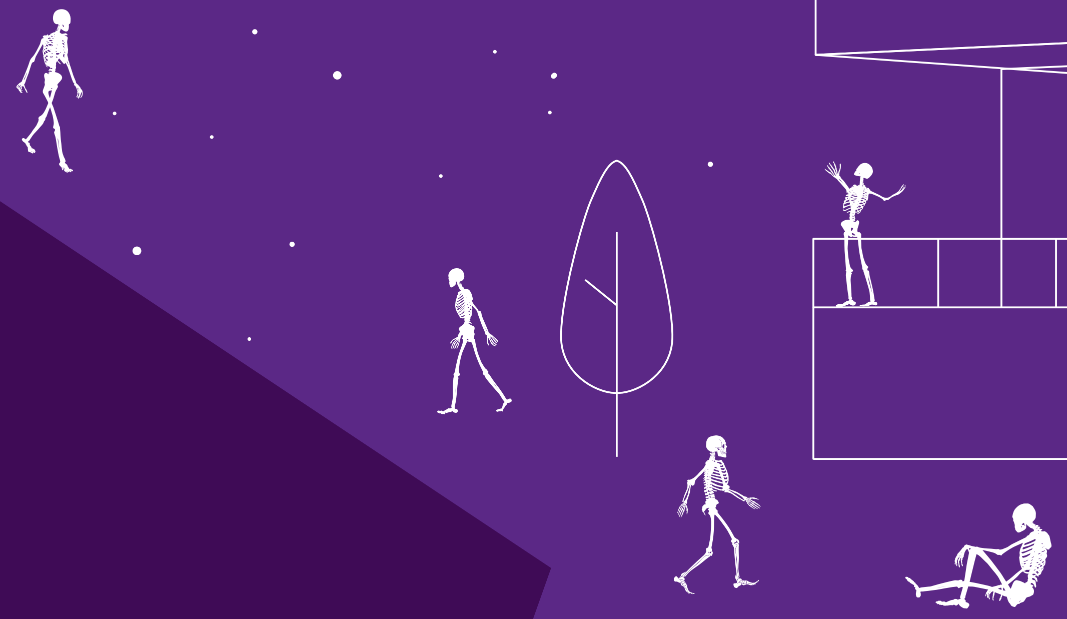

ESGAR 2019 / June 5 – 8 / Rome, Italy
30th Annual Meeting and Postgraduate Course

ESR
European Society of Radiology

European Society
ESGAR
Gastrointestinal and Abdominal Radiology

### ESGAR

### Annual Meeting and Postgraduate Course

### 2019

ESGAR 30 YEARS ANNUAL MEETINGS

### June 5-8

ROME
ITALY

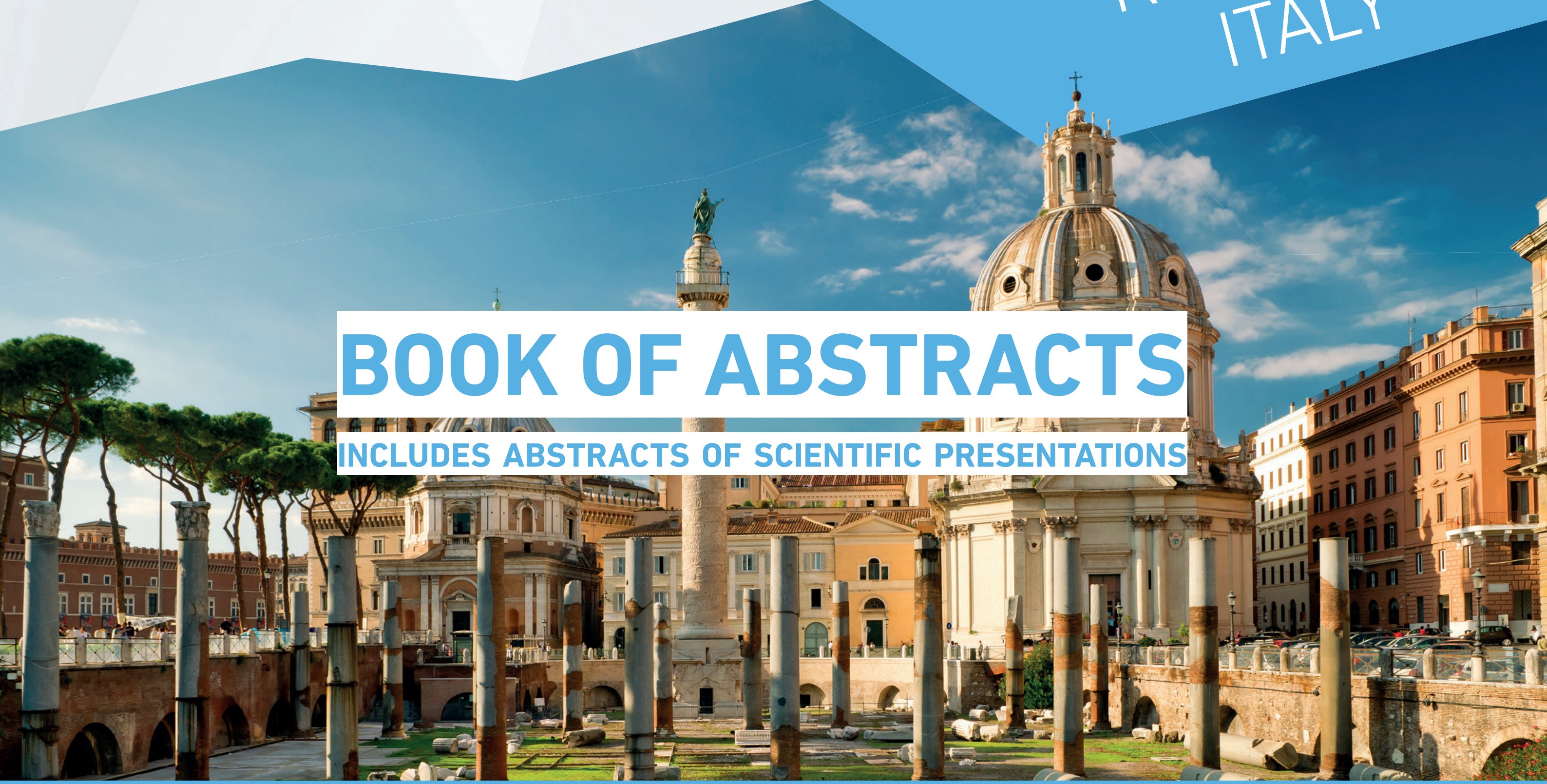

### BOOK OF ABSTRACTS

INCLUDES ABSTRACTS OF SCIENTIFIC PRESENTATIONS

*ORGANISING SECRETARIAT*
**Central ESGAR Office**
Esslinggasse 2/3
AT – 1010 Vienna
Phone: +43 1 535 89 27
Fax: +43 1 535 89 27 -15
E-Mail: office@esgar.org

*WEBSITE*
www.esgar.org

*CONFERENCE VENUE*
**Roma Convention Center**
La Nuvola – "The Cloud"
Viale Asia
IT – 00144 Rome

### CME CREDITS

The "ESGAR European Society of Gastrointestinal and Abdominal Radiology" (or) "ESGAR 2019 – 30th ANNUAL MEETING AND POSTGRADUATE COURSE" is accredited by the European Accreditation Council for Continuing Medical Education (EACCME) to provide the following CME activity for medical specialists. The EACCME is an institution of the European Union of Medical Specialists (UEMS), www.uems.net.

The ESGAR Annual Meeting and Postgraduate Course, Rome, Italy, 05/06/2019-08/06/2019 has been accredited by the European Accreditation Council for Continuing Medical Education (EACCME®) with 23 European CME credits (ECMEC®s). Each medical specialist should claim only those hours of credit that he/she actually spent in the educational activity.

### CONTRIBUTING SOCIETIES

Asian Society of Abdominal Radiology (ASAR), Since 2007
European Crohn's and Colitis Organisation (ECCO)
European Society of Urogenital Radiology
SAR Society of Abdominal Radiology

### SPONSORS

ESGAR wishes to gratefully acknowledge the support of its Corporate Members:

BAYER
BRACCO LIFE FROM INSIDE
GE Healthcare
Guerbet Contrast for Life
PHILIPS

The Final Programme of ESGAR 2019 is available on the ESGAR Website www.esgar.org

Date of publishing: June 2019

*Insights Into Imaging* (2019), 10(Suppl 2): 58
https://doi.org/10.1186/s13244-019-0748-0
Published Online: 04 June 2019

### *TABLE OF CONTENTS*

| | |
|---|---|
| Scientific Sessions, Thursday, June 6 (SS 1 – SS 5) | 8-21 |
| Scientific Sessions, Friday, June 7 (SS 6 – SS 10) | 22-34 |
| Scientific Sessions, Saturday, June 8 (SS 11 – SS 14) | 35-45 |
| Authors' Index | 47-51 |

### ESGAR EXECUTIVE COMMITTEE

PRESIDENT
*S. Halligan, London/UK*

PRESIDENT-ELECT
*R.G.H. Beets-Tan, Amsterdam/NL*

VICE PRESIDENT
*A. Laghi, Rome/IT*

SECRETARY/TREASURER
*M. Zins, Paris/FR*

PAST PRESIDENT
*C. Matos, Lisbon/PT*

EDUCATION COMMITTEE CHAIRMAN
*S. Jackson, Plymouth/UK*

MEMBERSHIP COMMITTEE CHAIRWOMAN
*M.A. Bali, Brussels/BE*

RESEARCH COMMITTEE CHAIRMAN
*J. Stoker, Amsterdam/NL*

WORKSHOP COMMITTEE CHAIRMAN
*G. Brancatelli, Palermo/IT*

MEETING PRESIDENT
*A. Laghi, Rome/IT*

PRE-MEETING PRESIDENT
*J. Stoker, Amsterdam/NL*

MEMBERS AT LARGE
*A. Ba-Ssalamah, Vienna/AT*
*V. Vilgrain, Clichy/FR*
*C.J. Zech, Basel/CH*

ESGAR EXECUTIVE DIRECTOR
*B. Lindlbauer, Vienna/AT*

### LANGUAGE ABSTRACT EDITOR

*R. Karia, Nottingham/UK*

### ESGAR 2019 MEETING PRESIDENT

*Prof. Andrea Laghi*
*Full Professor of Radiology*
*Department of Surgical and Medical*
*Sciences and Translational Medicine*
*“Sapienza” – University of Rome*
*Chairman of Radiology Unit -*
*Sant’Andrea University Hospital*
*Via di Grottarossa 1035*
*IT – 00189 Rome*

### ESGAR 2019 PROGRAMME COMMITTEE

CHAIRMEN
*M. Zins, Paris/FR*
*C. Matos, Lisbon/PT*

MEMBERS
*M.A. Bali, Brussels/BE*
*A. Ba-Ssalamah, Vienna/AT*
*R.G.H. Beets-Tan, Amsterdam/NL*
*G. Brancatelli, Palermo/IT*
*H. Fenlon, Dublin/IE*
*S. Halligan, London/UK*
*S. Jackson, Plymouth/UK*
*A. Laghi, Rome/IT*
*J. Stoker, Amsterdam/NL*
*V. Vilgrain, Clichy/FR*
*C.J. Zech, Basel/CH*

### ESGAR 2019 LOCAL ORGANISING COMMITTEE

*G. Argento, Rome/IT*
*B. Beomonte Zobel, Rome/IT*
*C. Bibbolino, Rome/IT*
*S. Canitano, Rieti/IT*
*C. Catalano, Rome/IT*
*M. Caterino, Rome/IT*
*P. Cerro, Rome/IT*
*R. Cianni, Rome/IT*
*P. D‘Andrea, Belluno/IT*
*V. David, Rome/IT*
*M. Galluzzo, Rome/IT*
*A. Giovagnoni, Ancona/IT*
*M. Guazzaroni, Rome/IT*
*E. Iannicelli, Rome/IT*
*R. Manfredi, Rome/IT*
*A. Orlacchio, Rome/IT*
*E. Pofi, Rome/IT*
*M. Rossi, Rome/IT*
*P. Schiroso, Rome/IT*
*F.M. Solivetti, Rome/IT*
*L. Tipaldi, Rome/IT*

*O. Akhan, Ankara/TR*
*C. Aubé, Angers/FR*
*M.A. Bali, Brussels/BE*
*I. Bargellini, Pisa/IT*
*T.V. Bartolotta, Palermo/IT*
*A. Ba-Ssalamah, Vienna/AT*
*R.G.H. Beets-Tan, Amsterdam/NL*
*E. Biscaldi, Genoa/IT*
*A. Blachar, Tel Aviv/IL*
*G. Brancatelli, Palermo/IT*
*D.J. Breen, Southampton/UK*
*F. Caseiro Alves, Coimbra/PT*
*N. Courcoutsakis, Alexandroupolis/GR*
*L. Crocetti, Pisa/IT*
*L. Curvo-Semedo, Coimbra/PT*
*R.F. Dondelinger, Liège/BE*
*M. D'Onofrio, Verona/IT*
*H. Fenlon, Dublin/IE*
*A.H. Freeman, Cambridge/UK*
*A. Furlan, Pittsburgh, PA/US*
*Y. Gandon, Rennes/FR*
*M.J. Gollub, New York, NY/US*
*S. Gourtsoyianni, Athens/GR*
*S. Gryspeerdt, Roeselare/BE*
*L. Guimaraes, North York, ON/CA*
*J.A. Guthrie, Leeds/UK*
*S. Halligan, London/UK*
*A. Hatzidakis, Heraklion/GR*
*T. Helmberger, Munich/DE*
*P. Huppert, Darmstadt/DE*
*F. Iafrate, Rome/IT*
*S. Jackson, Plymouth/UK*
*M. Karcaaltincaba, Ankara/TR*
*N. Kartalis, Stockholm/SE*
*A. Laghi, Rome/IT*
*D. Lambregts, Amsterdam/NL*
*M. Laniado, Dresden/DE*
*J.M. Lee, Seoul/KR*
*P. Lefere, Roselare/BE*
*M. Lewin, Villejuif/FR*
*O. Lucidarme, Paris/FR*
*M. Maas, Amsterdam/NL*
*A. Madureira, Porto/PT*
*M. Maher, Cork/IE*
*D.E. Malone, Dublin/IE*
*T. Mang, Vienna/AT*
*V. Maniatis, Sønderborg/DK*
*D. Marin, Durham, NC/US*
*L. Martí-Bonmatí, Valencia/ES*
*C. Matos, Lisbon/PT*
*Y. Menu, Paris/FR*
*G. Morana, Treviso/IT*
*G.H. Mostbeck, Vienna/AT*
*E. Neri, Pisa/IT*
*A. Palkó, Szeged/HU*
*P. Paolantonio, Rome/IT*
*N. Papanikolaou, Lisbon/PT*
*R. Pozzi Mucelli, Verona/IT*
*P. Prassopoulos, Thessaloniki/GR*
*E. Quaia, Padua/IT*
*G.A. Rollandi, Genoa/IT*
*S. Romano, Pozzuoli/IT*
*M. Ronot, Clichy/FR*
*L.H. Ros Mendoza, Zaragoza/ES*
*W. Schima, Vienna/AT*
*S. Schmidt Kobbe, Lausanne/CH*
*A.G. Schreyer, Regensburg/DE*
*O. Seror, Bondy/FR*
*S. Skehan, Dublin/IE*
*S. Somers, Dundas, ON/CA*
*M. Staunton, Cork/IE*
*S. Stojanovic, Novi Sad/RS*
*J. Stoker, Amsterdam/NL*
*J.-P. Tasu, Poitiers/FR*
*S.A. Taylor, London/UK*
*S. Terraz, Geneva/CH*
*D. Tolan, Leeds/UK*
*C. Triantopoulou, Athens/GR*
*V. Valek, Brno/CZ*
*V. Vilgrain, Clichy/FR*
*M.-P. Vullierme, Clichy/FR*
*D. Weishaupt, Zurich/CH*
*G.A. Zamboni, Verona/IT*
*C.J. Zech, Basel/CH*
*M. Zins, Paris/FR*

European Society

ESGAR

Gastrointestinal and Abdominal Radiology

**11:00 - 12:30** **Room Florence**

*Scientific Session SS 1*

### Diffuse liver diseases: assessing fibrosis and steatosis

### SS 1.1

### US attenuation imaging coefficient for the diagnosis and grading of hepatic steatosis: a pilot study in a biopsy-proven cohort

M. Dioguardi Burgio, M. Ronot, E. Reizine, P.-E. Rautou, L. Castera, V. Paradis, P. Garteiser, B. Van Beers, V. Vilgrain; Clichy/FR

**Purpose:** To assess the role of US attenuation imaging coefficient (ATI) in the diagnosis and quantification of liver steatosis.
**Material and methods:** 101 consecutive patients underwent liver biopsy and US ATI measurement the same day. Mean ATI value resulting from the 2 acquisitions presenting with the highest coefficient of correlation was used for statistical analysis. Liver steatosis was graded according to biopsy as absent (S0 <5%), mild (S1 5-33%), moderate (S2 33-66%), severe (S3 >66%). Correlation between ATI values and steatosis (%) was calculated using the Pearson correlation coefficient. T test was used to compare mean ATI values (S0 vs S1-3 and S0-1 vs S2-3 patients). Receiver operating characteristic curve analysis was used to assess the diagnostic performance of ATI in diagnosing steatosis.
**Results:** 43 (42%), 35 (35%), 12 (12%) and 11 (11%) patients were classified as S0, S1, S2 and S3, respectively. ATI was correlated with % of steatosis on pathology (r=0.58, p<0.01). Patients with steatosis had higher ATI value than patients without steatosis (0.77±0.13 dB/cm/MHz for S1-3 patients vs. 0.63±0.09 dB/cm/MHz for S0 patients; p<0.01, area under (AU) ROC=0.805). Patients with moderate/severe steatosis had higher ATI value than patients with no/mild steatosis (0.85±0.11 dB/cm/MHz for S2-S3 patients vs. 0.667±0.11 for S0-S1 patients; p<0.01, AUROC=0.892). ATI>0.69 dB/cm/MHz showed 75.9% sensitivity and 86% specificity in diagnosing steatosis (S1-3), and ATI> 0.72 dB/cm/MHz had 95.6% sensitivity and 74.4% specificity in diagnosis of moderate or severe steatosis (S2-S3).
**Conclusion:** ATI is promising for the non-invasive diagnosis and quantification of liver steatosis.

### SS 1.2

### Evaluation of fatty liver with US attenuation imaging: a prospective study using histopathology as a standard reference

J. Bae, D.H. Lee, J.Y. Lee, H. Kim, J. Lee, E.J. Cho, Y.B. Lee, J.K. Han, B.I. Choi; Seoul/KR

**Purpose:**
To evaluate the diagnostic performance of attenuation imaging (ATI) in the assessment of hepatic steatosis using histopathology as a standard reference.
**Material and methods:** We prospectively enrolled 108 consecutive patients (35 male and 73 female patients; median age, 54.0 years; age range, 19–81 years) who underwent percutaneous liver biopsy for evaluation of diffuse liver disease between January 2018 and November 2018. Grayscale US examinations with ATI were performed just before biopsy, and an attenuation coefficient (AC) was obtained from each patient. The degree of hepatic steatosis, fibrosis stage and necroinflammatory activity were assessed on histopathologic examination. The significant determinant factor for the AC was determined by a linear regression analysis, and the diagnostic performance of the AC in detecting each hepatic steatosis stage was evaluated by receiver operating characteristic (ROC) analysis.
**Results:** The AC was successfully obtained in all patients with less than 10 seconds of examination time. The distribution of hepatic steatosis grade was 53/11/22/16/6 for none/mild (<10%)/mild (≥10%)/moderate/severe steatosis, respectively, according to histopathology. The area under the ROC curve, sensitivity, specificity and optimal cut-off AC value for detection of hepatic steatosis ≥5%, ≥10%, ≥33% and >66% were 0.843, 74.5%, 77.4%, 0.635; 0.876, 79.5%, 82.8%, 0.660; 0.886, 86.4%, 81.4%, 0.700; 0.926, 100.0%, 82.4%, and 0.745, respectively. Multivariate analysis revealed that the degree of steatosis was the only significant determinant factor for the AC.
**Conclusion:** The AC from ATI provided good diagnostic performance in detecting the varying degrees of hepatic steatosis. The degree of steatosis was the only significant factor affecting the AC.

### SS 1.3

### Liver MRI susceptibility-weighted imaging and T2* mapping in the presence of steatosis and fibrosis

V. Obmann, C. Marx, J. Hrycyk, L. Ebner, M. Ith, C. Gräni, A. Berzigotti, J. Heverhagen, A. Christe, A. Huber; Bern/CH

**Purpose:** To show that both susceptibility-weighted imaging (SWI) and T2* mapping are dependent on liver steatosis, which should be taken into account when using these parameters to grade liver fibrosis and cirrhosis.
**Material and methods:** 184 patients without focal liver disease underwent multiparametric MRI at 3T including susceptibility-weighted imaging (SWI), T1/T2* mapping, proton density fat fraction (PDFF) quantification and MR elastography. SWI, T2* and T1 measured in the liver (4 locations), as well as their liver-to-muscle-ratio (LMR, measured in the paraspinal muscles) were compared between patients with different steatosis grades (PDFF <5%, 5-10%, 10-20% and >20%) and between patients with normal, slightly and moderately increased liver stiffness (<2.8 kPa, 2.8-3.5 kPa and >3.5 kPa, respectively). For statistics, ANOVA with Bonferroni-corrected post hoc tests as well as multivariate analysis were used.
**Results:** Both SWI (98±30, 89±26, 65±33, 42±16) and T2* (22±4, 19±5, 17±4, 15±2) were highly dependent on the degree of liver steatosis (p<0.001). In Bonferroni-corrected post hoc analysis, SWI showed significant differentiation in 4/6 comparisons, while T2* was significant in 3/6 comparisons. However, SWI allowed a better differentiation between liver fibrosis grades (p <0.001) than T2* (p=0.05). The liver-to-muscle ratios (LMR) were not superior to the respective parameters alone. Best prediction of liver fibrosis could be achieved by including PDFF, age, T1 and SWI in a multiparametric model (combined $r^2$=0.44).
**Conclusion:** Both SWI and T2* mapping are highly dependent on liver steatosis grades. Nevertheless, both parameters are useful predictors for liver fibrosis when using a multiparametric approach.

### SS 1.4

### Quantitative hepatorenal B-mode ratio for the noninvasive diagnosis of liver steatosis

A. Paisant, A. Moret, J. Boursier, J. Lebigot, C. Aubé; Angers/FR

**Purpose:** The aim of the study was to evaluate the quantitative hepatorenal sonographic index of the B-mode ratio (Supersonic Imaging; Aix-France) for the noninvasive diagnostic and quantification of steatosis.

**Material and methods:** Between January and October 2018, all patients with an indication of liver biopsy for suspicion of chronic liver disease were prospectively included. For each patient, 3 B-value measurements were acquired the same day followed by a liver biopsy. The mean of the values was used for the analyses. Steatosis were staged histologically as follows: S0 (0-<5%); S1 (5-<33%); S2 (33%-<66%); S3 (≥66%).

**Results:** 89 patients were included. Two patients were excluded, one because of a right nephrectomy, one due to the lack of the B-mode ratio recording. There were 59 males and 28 females, mean age 58.1 ± 12.2 y.o. and mean BMI 33.3 ±7.0 kg/m². Mean B-ratio values were 0.98±0.24 for S0; 1.72±0.69 for S1; 1.92±0.84 for S2 and 1.98±0.81 for S4. The best cut-off value was 1.21 for S0 versus S1-S3 (Se=0.92; Sp=0.85; area under the receiver operating characteristic curve (AUROC)=0.952, p<0.001); and 1.55 for S0-1 versus S2-S3 (Se=0.66; Sp=0.63; AUROC=0.686, p=0.003). A B-ratio of 1.27 ruled in steatosis (positive predictive value=96.8%) and 0.96 ruled out steatosis (negative predictive value=100%).

**Conclusion:** B-mode ratio is an easy-to-perform and accurate tool for the noninvasive diagnostic of steatosis. Its performances are lower to differentiate mild to severe steatosis.

### SS 1.5

*Withdrawn by the authors*

### SS 1.6

### The value of intravoxel incoherent motion in detecting and staging liver fibrosis: a systematic review and meta-analysis

Z. Ye, Y. Wei, B. Song; Chengdu/CN

**Purpose:** To evaluate the diagnostic performance of intravoxel incoherent motion (IVIM) in detecting and staging liver fibrosis (LF).

**Material and methods:** A comprehensive literature search was conducted to identify studies on the diagnostic accuracy of IVIM for the assessment of histology-proven LF. The degrees of LF were defined as F0 (no fibrosis), F1 (portal fibrosis without septa), F2 (periportal fibrosis with few septa), F3 (septal fibrosis) and F4 (cirrhosis), according to METAVIR scoring system. Data were extracted to calculate the pooled sensitivity, specificity, positive and negative likelihood ratios, as well as the area under the summary receiver operating characteristic curve (AUC) in each group.

**Results:** 10 studies with 679 subjects were included in this meta-analysis. The pooled sensitivity, specificity, positive likelihood ratio and negative likelihood ratio were estimated to be 0.77 (95% confidence interval: 0.71-0.83), 0.82 (0.74-0.87), 3.99(1.89-8.41) and 0.33 (0.18-0.60) for ≥F1 detection with IVIM; 0.87 (0.82-0.91), 0.77 (0.71-0.83), 3.51 (2.16-5.71) and 0.18 (0.09-0.37) for ≥F2 detection; 0.86 (0.79-0.92), 0.84 (0.78-0.90), 5.33 (3.69-7.71) and 0.20 (0.06-0.63) for ≥F3 detection, and 0.85 (0.79-0.91), 0.78 (0.73-0.82), 3.56 (2.49-5.09) and 0.22 (0.11-0.44) for ≥F4 detection, respectively. The AUCs for ≥F1, ≥F2, ≥F3, ≥F4 detection were 0.86 (0.79-0.94), 0.90 (0.86-0.94), 0.90 (0.88-0.93) and 0.89 (0.87-0.93), respectively. Substantial heterogeneity was observed. No publication bias was detected.

**Conclusion:** IVIM shows high diagnostic accuracy in detecting and staging LF and may serve as a noninvasive tool for LF evaluation.

### SS 1.7

### Effect of nodular regenerative hyperplasia on liver stiffness measurement in magnetic resonance elastography

R. Cannella[1], M.I. Minervini[2], V. Rachakonda[2], A.A. Borhani[2], A. Furlan[2]; [1]Palermo/IT, [2]Pittsburgh, PA/US

**Purpose:** To investigate the effect of nodular regenerative hyperplasia (NRH) on the liver stiffness measurement (LSM) obtained with magnetic resonance elastography (MRE).

**Material and methods:** This retrospective, IRB-approved study included 39 subjects with NRH (Group 1) and no or minimal fibrosis (≤F2), a control group (Group 2) made of 30 subjects with non-advanced fibrosis (F0-F2) and a control group (Group 3) made of 30 subjects with advanced fibrosis (F3-F4), all with available MRE. LSM was measured in each subject along with the assessment of hepatic morphological features of cirrhosis and signs of portal hypertension. The significance of the difference in mean LSM between Groups 1 and 2 and between Groups 1 and 3 was evaluated using the Mann-Whitney U test. The difference in the distribution of imaging features among groups was assessed using the Pearson $\chi^2$ or Fisher exact test. Statistical significance was set at P<0.05.

**Results:** The mean±SD LSM in Group 1 (3.52±1.08 kPa) was significantly higher compared to Group 2 (2.91±0.52 kPa, P=0.028) and significantly lower compared to Group 3 (7.18±2.08 kPa, P<0.001). Twelve (31%) patients with NRH had LSM ≥ 4.11 kPa, and 6 (15%) patients had LSM ≥ 4.71 kPa. Surface nodularity (p=0.032), caudate lobe hypertrophy (P=0.004) and enlarged periportal space (P=0.039) were more commonly visualized in Group 1 than in Group 2. At least one feature of portal hypertension was observed in 17 (43%) subjects with NRH.

**Conclusion:** NRH increases the LSM obtained with MRE and may represent a confounding factor when using liver stiffness for the non-invasive diagnosis of fibrosis.

### SS 1.8

### Evaluation of liver fibrosis and necro-inflammation using stiffness and dispersion slope from 2D-shear wave elastography

D.H. Lee, E.S. Lee; Seoul/KR

**Purpose:** To prospectively evaluate whether liver stiffness (LS) and shear wave (SW) dispersion slope obtained from 2D-shear wave elastography (SWE) could evaluate fibrosis stage and necro-inflammatory activity (NIA) using histopathology as a standard reference.

**Material and methods:** We prospectively enrolled 114 patients who underwent percutaneous liver biopsy. All patients underwent SWE examination just before biopsy, and LS (kilopascal [kPa]) as well as SW dispersion slope ([m/s/]/kHz) were obtained. On histopathologic examination, fibrosis stage (F0-F4) and NIA (A0-A4) were assessed. Multivariate linear regression analysis was done to determine the significant affecting factors for LS and SW dispersion slope. Diagnostic performance of each variable in staging fibrosis and grading NIA was assessed using receiver operating characteristic (ROC) analysis.

**Results:** Both LS and SW dispersion slope were significantly different among the different fibrosis stage and NIA grade (P<0.001). Only fibrosis stage was significantly associated with LS (P<0.001). However, both fibrosis stage (P<0.001) and NIA grade (P<0.001) were significant determinant factors for SW dispersion slope. LS provided significantly better diagnostic performance in detecting cirrhosis than SW dispersion slope (area under curve [AUC]: 0.950 vs. 0.812, P=0.020). The optimal cut-off LS were 5.4 kPa, 7.8 kPa, 9.4 kPa and 12.2 kPa, respectively, for F≥1, F≥2, F≥3 and F=4. Diagnostic performance of SW dispersion slope in detecting A≥3 of NIA was marginally higher than LS (AUC: 0.782 vs. 0.836, P=0.065).

**Conclusion:** LS obtained from 2D-SWE provided quite a good diagnostic performance in evaluating liver fibrosis stage, and SW dispersion slope might help assess the NIA grade.

### SS 1.9

### Reduction in MRI liver proton density fat fraction in patients with type 2 diabetes mellitus following treatment with duodenal mucosal resurfacing

N. Sakai[1], A. Bainbridge[1], D. Maggs[2], M. Hall-Craggs[1], S.A. Taylor[1], M.D. Chouhan[1]; [1]London/UK, [2]Lexington, MA/US

**Purpose:** To investigate the effects of endoscopic duodenal mucosal resurfacing (DMR) in patients with type 2 diabetes mellitus (T2DM) on liver fat fraction (FF) and liver iron content (LIC) using MRI proton density fat fraction (PDFF).
**Material and methods:** Revita-2 is a phase II, blinded, sham-controlled, international multi-site, multi-scanner vendor cross-over trial (NCT02879383). An initial open-label training cohort (n=20) received liver MRI scans at baseline and 12 weeks following DMR on the same MRI scanner across six sites, using vendor-derived PDFF sequences (TR=5-10ms, TE of first echo= 1-2ms, number of echoes=6, alpha=3 degrees, 6mm slice thickness, 2-2.5 mm isotropic inplane resolution). For each scan, circular region-of-interest (ROI) measuring was placed in each of the 9 Couinaud liver segments, colocalised on PDFF and T2* maps (for LIC). Longitudinal measurement stability was confirmed using custom-built fat-water liquid-emulsion-based phantoms.
**Results:** All liver segments demonstrated statistically significant reductions in FF (p<0.01), with an average 12-week reduction across all liver segments in absolute FF of -5.9% (p=0.008) and relative FF of -28.2% (p=0.002). The average 12-week change in absolute and relative LIC across all liver segments was -0.87umol/g (p=0.1197) and -2.8% (p=0.3729), respectively.
**Conclusion:** MRI-PDFF demonstrated significant changes in absolute and relative liver FF 12 weeks after DMR. There was no significant change in LIC 12 weeks after DMR. Further longitudinal assessment of liver FF and LIC in this cohort will assess potential sustained reductions in liver FF, longitudinal effects on LIC and correlation with biochemical markers of diabetes.

### SS 1.10

### CT-based liver surface nodularity for the detection of clinically significant portal hypertension: defining measurement quality criteria

R. Sartoris, A. Nivolli, M. Lazareth, P.-E. Rautou, V. Vilgrain, M. Ronot; Clichy/FR

**Purpose:** To establish measurement quality criteria for liver surface nodularity (LSN) quantification on CT in the setting of portal hypertension.
**Material and methods:** 77 consecutive cirrhotic patients (50 males, mean 62±6 years), including 30 with clinically significant portal hypertension (CSPH), 39%), underwent CT and hepatic venous portal gradient. Three independent readers performed 15 LSN measurements/patient on contrast-enhanced CT using a dedicated software. Quality criteria were identified to maximize LSN accuracy while minimizing inter- and intrareader variability. LSN was computed both as median and mean using 1 to 15 individual measurements. Accuracy of LSN for the diagnosis of CSPH was assessed using receiver operating characteristic (ROC) curve analysis. Variabilities were assessed by computing the intraclass correlation coefficient (ICC), coefficient of variation (CV) and Bland-Altman plot (BA).
**Results:** Using 1 to 15 individual measurements, mean and median LSN showed similar diagnostic performance (area under (AU) ROCs from 0.79±0.05 to 0.91±0.04 and from 0.86±0.04 to 0.91±0.03, all pair-wise comparisons p>0.05). AUROCs of mean LSN increased from 1 to 8, and plateaued from 8 to 15 measurements. Using mean LSN values, inter- and intra-reader variability decreased when using 1 to 15 individual measurements, but improvement was marginal > 8 measurements. With 8 measurements, intra- and inter-observer variability was low (ICC 0.90 [0.84-0.94], CV 7.9%, BA +4.2% [-15.3;+23.7%] for intra, and ICC 0.93 [0.89-0.95], CV 9.1%, and BA +4.8% [-17.5;+27.1%] for inter).
**Conclusion:** We propose performing at least 8 valid measurements to compute mean LSN value for the detection of CSPH on CT.

**11:00 - 12:30** **Room Pisa**

*Scientific Session SS 2*

### Rectal cancer: diagnosis and imaging of treatment response

### SS 2.1

### Performance of texture analysis in predicting tumoural response to neoadjuvant chemoradiotherapy in rectal cancer patients studied with 3T MR

M. Zerunian[1], D. Bellini[2], M. Rengo[2], N. Panvini[2], D. De Santis[1], D. Caruso[1], A. Laghi[1]; [1]Rome/IT, [2]Latina/IT

**Purpose:** To determine the performance of texture analysis (TA) in predicting tumoral response in colon–rectal cancer (CRC).
**Material and methods:** Forty consecutive CRC patients were prospectively enrolled and underwent 3T-MRI pre- and post-chemo-radiotherapy (CRT). After surgery, the histological results were considered the reference standard. A region-of-interest was manually drawn around the tumour area on axial T2-w slice and analyzed with a dedicated software (TextRad) extrapolating the following parameters: skewness, kurtosis, entropy, and mean value of positive pixels (MPP). Non-parametric Mann-Whitney U test was used to compare TA parameters and the response rate among complete responders (CR), partial responders (PR), and non-responders (NR) before and after CRT. Receiver operating characteristic (ROC) curves were used to assess the discriminatory power of TA to predict complete response to CRT.
**Results:** Thirteen patients (32.5%) showed CR, twenty-two patients (55%) showed PR and five patients (12.5%) were classified as NR. After CRT, CR showed significant reduction of entropy (6.17±0.54 vs 6.49±0.43), kurtosis (0.72±1.05 vs 2.60±2.01) and MPP (306.33±168.97 vs 414.24±219.26); all P <= 0.002. After CRT, PR/NR showed significant reduction of entropy (6.47 ± 0.57 vs 6.70± 0.50) and skewness (0.53±0.75 vs 0.35±0.67); all P <=0.029. After CRT, CR showed significant lower kurtosis (0.33 ± 0.65) and MPP (262.19 ± 135.28), compared to PR/NR (kurtosis: 1.87±1.85; MPP: 357.54±155.07); all P <= 0.042. Entropy was the only TA parameter showing significance in the predicting CR(AUC 0.64[95% CI: 0.57-0.71]), P < 0.001 with the best cut-off value of >= 6.68, with a sensitivity of 76.9% and a specificity of 38.46% in predicting CR.
**Conclusion:** TA from T2 images potentially plays an important role as imaging biomarkers of tumoral response to neoadjuvant-CRT in CRC.

Scientific Sessions

Authors' Index

### SS 2.2

### Comparison of MRI morphology, diffusion-weighted imaging patterns and a combination of both in assessing complete response in patients with locally advanced rectal cancer treated with neoadjuvant long-course chemoradiotherapy

A. Chandramohan, U. Siddiqui, R. Mittal, A. Eapen, M.R. Jesudason, T.S. Ram, A. Singh, D. Londhe; Vellore/IN

**Purpose:** To compare the diagnostic performance of MRI (T2-HR), diffusion-weighted imaging (DWI) and their combination in determining complete response to neoadjuvant chemoradiotherapy (LCCRT) in patients with locally advanced rectal cancer.
**Material and methods:** In this retrospective study of 312 patients (mean age 47.3+/-14.3 years, range 19-86 years, M:F= 204:108) with locally advanced rectal cancer treated with LCCRT and surgery, two radiologists blinded to the surgical histopathology, independently reviewed MRI performed pre- and post-LCCRT. Diagnostic performance of morphological assessment on T2-HR images, pattern on DWI and combination of both in determining complete (CR) versus incomplete (IR) response was assessed with pathological response as the reference standard. Either restoration of rectal wall morphology or thin band of fibrosis less than normal rectal wall thickness or complete absence of restricted diffusion was considered as MRI-CR. Level of confidence was recorded on a scale of 1 to 5, 5 being very confident.
**Results:** Rate of pathological CR was 16%. Pattern-based interpretation of DWI had the highest accuracy (AUC = 0.822), followed closely by combined interpretation of DWI and T2-HR images (AUC of 0.806), p<0.001. Interobserver agreement was substantial (k=0.688) for combined DWI + T2-HR images and moderate (k=0.402) for DWI alone with both observers exhibiting significantly higher confidence with the combined approach, p<0.05. Morphology-based assessment on T2-HR alone had poor sensitivity of 8% and accuracy (AUC= 0.532) and interobserver agreement (k=0.231), p>0.05.
**Conclusion:** MR-complete response to neoadjuvant LCCRT can be determined with high accuracy and confidence with a combination of DWI signal pattern and morphology on T2-HR images.

### SS 2.3

### Artificial intelligence automatic identification of complete- and non-responders using texture analysis of rectal cancer 3T MR images performed before, during and after neoadjuvant chemoradiotherapy

M. Zerunian[1], R. Ferrari[1], C. Mancini Terracciano[1], C. Voena[1], M. Rengo[2], R. Paramatti[1], D. Caruso[1], R. Faccini[1], A. Laghi[1]; [1]Rome/IT, [2]Latina/IT

**Purpose:** To evaluate an artificial intelligence (AI) model for classification of complete (CR) and non-responder (NR) patients with rectal cancer treated with neoadjuvant chemo-radiotherapy (CRT). AI input is based on texture analysis of high-resolution 3T MR images performed before, during and after CRT.
**Material and methods:** 55 consecutive patients with rectal cancer were prospectively enrolled in this study. Patients underwent 3T T2-weighted (T2w) MRI before, during and after CRT; volumetric regions of interest (VOI) around the tumor were manually drawn. All patients underwent complete surgical total mesorectal excision and the gross specimen was used as reference standard. Two AI models were built with the most statistically significant features training a random forest (RF) classifier on 28 patients (training cohort). The model performances were estimated on 27 patients (validation cohort) using a ROC curve analysis and a decision curve analysis.
**Results:** Textural analysis points to a lower intra-tumor heterogeneity at the pre- and during-treatment stage for CR patients with a characteristic time evolution of some of the textural features. The obtained AI model shows good discriminatory ability with a ROC area under curve (AUC) of 0.94 (95% CI: 0.89,0.99) in the validation cohort. The discriminatory power of the AI model built for NR discrimination has a ROC AUC of 0.85 (95% CI: 0.80,0.90). Decision curve analysis confirms clinical usefulness of the models.
**Conclusion:** AI models based on textural parameters of MR images of patients with rectal cancer taken before, during and after CRT show good performances for stratification of response to therapy.

### SS 2.4

### 18F-fluoro-2-deoxy-D-glucose-avid presacral soft tissue mass in previously treated rectal cancer: diagnostic outcome and additional value of MRI, including diffusion-weighted imaging

J.P. Pennings, R.J. De Haas, K.J.A. Murshid, K.P. De Jong, R.A.J.O. Dierckx, T.C. Kwee; Groningen/NL

**Purpose:** This study aimed to determine the positive predictive value (PPV) of positron emission tomography/computed tomography (PET/CT) with an $^{18}$F-fluoro-2-deoxy-D-glucose (FDG)-avid presacral lesion for locally recurrent rectal cancer, and the additional value of MRI, including diffusion-weighted imaging (DWI).
**Material and methods:** This retrospective study included 38 patients who completed primary rectal cancer treatment and who presented with a suspicious FDG-avid presacral lesion on PET/CT. Twenty-seven patients also underwent MRI, of whom 24 with DWI. PPV of FDG-PET/CT and additional value of MRI, including DWI, for the diagnosis of recurrent presacral cancer were determined.
**Results:** The PPV of PET/CT with an FDG-avid presacral lesion for the diagnosis of locally recurrent rectal cancer was 58% (22/38). Air in the FDG-avid presacral lesion, as visible on the CT component of the PET/CT examination, favoured the diagnosis of benign presacral tissue with a sensitivity of 56.3% (9/16) and a specificity 81.8% (18/22). Areas under the receiver operating characteristic curve (AUCs) of MRI without DWI for the diagnosis of locally recurrent rectal cancer in FDG-avid presacral tissue were 0.765 and 0.840, for observers 1 and 2. AUCs of MRI with DWI were 0.803 and 0.811, for observers 1 and 2. There were no significant differences among any of these AUCs (P=0.169 to 0.906).
**Conclusion:** FDG-PET/CT has a poor PPV for locally recurrent rectal cancer in the presacral space. The observation of air in the FDG-avid presacral lesion and additional MRI assessment are diagnostically helpful, without a significant additional value of DWI.

### SS 2.5

### Diagnostic value of dynamic perfusion MRI in patients with locally advanced rectal cancer in the assessment of chemo-radiation treatment: relation to tumor regression grade at histology

P.P. Arcuri[1], A.K. Sikora[1], S. Roccia[1], G. Fodero[1], V. Aiello[1], C. Bertucci[2], E. Mazzei[1], D. Laganà[1]; [1]Catanzaro/IT, [2]Buckingham/UK

**Purpose:** To investigate the value of dynamic contrast-enhanced perfusion MRI parameters in the evaluation of the response to chemo-radiation therapy in patients with locally advanced rectal cancer in comparison with histology.
**Material and methods:** We evaluated, retrospectively, thirty-eight patients affected by rectal adenocarcinoma (confirmed by biopsy) studied with dynamic contrast-enhanced MR sequence, before (MR-T0) and after chemo-radiation therapy (MR-T1). The protocol included T1 gadolinium-enhanced THRIVE sequence. A region of Interest (ROI) was manually drawn on tumor tissue and normal rectal wall. The following parameters were calculated and statistically analyzed: maximum enhancement (ME), relative enhancement (RE), time to peak (TTP), wash-in rate (W-inR) and wash-out rate (W-outR). Data were expressed in terms of median value ± range. Kruskal–Wallis non-parametric test was performed. Sensitivity, specificity and accuracy were assessed. Fisher's exact test was used to evaluate statistical significance. A p value <0.05 was considered statistically significant. The biopsy of the masses was the gold standard of reference. Perfusion parameters were related to pathologic tumor regression grade (Mandard's criteria; TRG1=complete regression, TRG5=no regression).
**Results:** Thirteen tumors (35%) showed complete or subtotal regression (TRG1-2) at histology and were classified as responders; twenty-five tumors (65%) were classified as non-responders (TRG3-5). At baseline (MR-T0), no significant difference in perfusion parameters was found between responders and non-responders. After six months post-chemo-radiation therapy (MR-T1), responders showed significantly (p< 0.05) lower perfusion values [ME(%)=93±38, RE(%)=99±41, TTP(s)=21±36, W-inR (l/s)=84±28, W-outR(l/s)=96±41] compared to non-responders [ME(%)=158±31, RE(%)=182±43, TTP(s)=42±21, W-inR (l/s)=178±33, W-outR (l/s)=178±36]. Sensitivity, specificity and diagnostic accuracy were, respectively, ME=81%, 78%, 83%; RE=77%, 75%, 73%; TTP=78%, 81%, 84%, W-inR=69%, 72%, 74%; W-outR=71%, 68%, 74%. In non-responders, there was no significant difference between perfusion values at MR-T0 and MR-T1.
**Conclusion:** Dynamic contrast perfusion-MRI analysis represents a complementary diagnostic tool for identifying vascularity characteristics of tumor tissue in locally advanced rectal cancer, useful in the assessment of treatment response.

### SS 2.6

### T2-weighted, apparent diffusion coefficient and positron emission tomography texture analysis of locally advanced rectal cancer after preoperative chemoradiotherapy: correlation with tumour regression grade

F. Crimì, V. Aldegheri, G. Spolverato, C. Lacognata, P. Zucchetta, A. Barison, L. Albertoni, C. Campi, R. Stramare, E. Quaia; Padua/IT

**Purpose:** To investigate the correlation among T2-weighted (T2w) images, apparent diffusion coefficient (ADC) maps, fludeoxyglucose ($^{18}$F-FDG) positron emission tomography (PET) image texture in the volume of interest (VOI) of the primary lesion and the tumor regression grade (TRG) in patients affected by locally advanced rectal cancer (LARC) after preoperative chemoradiotherapy (pCRT).
**Material and methods:** 19 patients (16M) affected by LARC were prospectively enrolled and underwent $^{18}$F-FDG PET/MRI for restaging after pCRT. The MRI protocol included an oblique-axial T2w-sequence and an axial diffusion-weighted imaging (DWI)-sequence. ADC maps and PET images of the pelvis were re-sliced and re-oriented with a specific software (PMOD) to perfectly match to the T2w images. A region of interest (ROI) was manually drawn along the boundaries of each slice on the T2w images including the rectal cancer, obtaining a VOI; each ROI was then copied on the corresponding PET and ADC datasets. Voxel-based standardized uptake values (SUVs), ADC values and T2w-intensity values were collected from the entire VOI and mean, skewness and kurtosis were calculated. Spearman's correlation coefficient was applied to evaluate the correlation among the variables and TRG (according to Mandard).
**Results:** Six LARC showed a complete regression (TRG1). A significant positive correlation was found between SUV mean values (ρ=0.480; p=0.037) T2w-intensity mean values (ρ=0.505; p=0.037) and TRG. No significant correlation was detected between other variables and TRG.
**Conclusion:** The preliminary results of our study showed that post-pCRT texture analysis of SUV values and T2w intensity signal are predictors of TRG in LARC. Further studies on a larger sample are necessary to assess the role of ADC values.

### SS 2.7

### MR of rectal cancer response to therapy: comparison between 3.0 and 1.5 Tesla

E. Lucertini, M. Zerunian, A. Guarnera, F. Pucciarelli, D. De Santis, D. Caruso, A. Laghi; Rome/IT

**Purpose:** To compare image quality on T2-weighted (T2w), diffusion-weighted imaging (DWI) and apparent diffusion coefficient maps (ADC) between 3.0T and 1.5T MRI in patients with rectal cancer pre-, during and post-neoadjuvant chemoradiotherapy (CRT).
**Material and methods:** 22 consecutive patients with rectal cancer were prospectively analyzed on both 3.0T MRI and 1.5T MRI acquiring a dedicated axial oblique T2w, DWI and ADC maps pre-, during and post-CRT. Two expert radiologists analyzed a total of 396 slices drawing a region-of-interest (ROI) of the tumor and obturator internus muscle. Signal intensity (SI) and relative SI (rSI) were analyzed. Additionally, a sub-analysis evaluating the SI difference before and after-CRT (ΔS.I.pre-post) in complete responder patients (CR) was performed.
**Results:** Significant differences were observed for T2w on 3.0T compared to 1.5T MRI for pre-CRT (632.60 ± 183.02 vs. 341.88 ± 120.98), during CRT (511.85 ± 193.59 vs. 318.23 ± 107.77) and post-CRT (525.08 ± 149.67 vs. 293.68 ± 99.17), and for DWI for pre-CRT (321.32 ± 176.99 vs. 70.77 ± 21.44), during CRT (184.35 ± 95.47 vs. 51.47 ± 17.00) and post-CRT (136.33 ± 70.03 vs. 43.52 ± 14.84) (all P <0.001), ΔSI CR showed significant differences between 3.0T MRI and 1.5T MRI for DWI-ΔSI CR (188.39 ± 166.90 vs 30.45 ± 21.73; P value 0.02) and ADC-ΔSI CR (-0.58 ± 0.27 vs -0.21 ± 0.24; P value 0.02).
**Conclusion:** T2wSI and DWISI showed significant differences for 3.0T compared to 1.5T. A significant DWI-ΔSI CR and ADC-ΔSI CR on 3.0T may provide a better visual assessment in discriminating response to therapy.

### *SS 2.8*

### Association between texture analysis parameters and oncogenic Kirsten-ras mutation in patients with primary rectal cancer

S.H. Kim[1], S.J. Jo[1], S.J. Park[2]; [1]Busan/KR, [2]Seoul/KR

**Purpose:** To evaluate the association between the texture parameters based on T2-weighted images (T2WI) and Kirsten-ras (KRAS) mutation in patients with primary rectal cancer.
**Material and methods:** Forty-six rectal adenocarcinoma patients who had undergone preoperative rectal MRI and subsequent surgical resection were included in the study. Texture analysis was performed on axial T2WI by a radiologist using a dedicated software (Medical Imaging Solution for Segmentation and Texture Analysis, MISSTA, Seoul, South Korea). Regions of interest were drawn along the tumor border to cover the whole tumor volume. Representative texture parameters of each tumor were calculated and analyzed. The association between texture parameters and KRAS mutation was analyzed using the Mann-Whitney U test. To extract an optimal cut-off value for predicting KRAS mutation, a receiver operating characteristic (ROC) curve analysis was used and corresponding predictive values were estimated. Molecular biologic results on KRAS mutation served as the reference standard.
**Results:** Skewness in the mutant group (n=22) was significantly higher than that in the wild-type group (n=24) (0.221 ± 0.283; -0.006 ± 0.178, respectively, P=0.003). The AUC of skewness was 0.757 (95% CI, 0.606 to 0.872, P=0.0005) with a maximum accuracy of 71%, sensitivity of 64%, specificity of 78%, PPV of 74% and NPV of 69%. Other texture parameters were not associated with KRAS mutation (P>0.05).
**Conclusion:** Skewness derived from texture analysis based on T2WI was associated with KRAS mutation in patients with primary rectal cancer, and the estimated accuracy for prediction of KRAS mutation was 71%.

### *SS 2.9*

### Predicting neoadjuvant therapy response in locally advanced rectal cancer using texture features

V. Giannini, S. Mazzetti, I. Bertotto, E. Delmastro, P. Gabriele, D. Regge; Candiolo/IT

**Purpose:** To implement a multivariate classifier using texture features derived from positron emission tomography (PET) acquisitions, for predicting pathological response to neoadjuvant chemo-radiotherapy (CRT) in locally advanced rectal carcinoma (LARC).
**Material and methods:** 47 patients with a) histologically diagnosis of LARC, b) pre-treatment PET/CT, c) neoadjuvant treatment consisting of 46-55 Gy in 23-30 RT fractions alone or in association with either infusional 5-FU or oral capecitabine, and d) total mesorectal excision were included in this study. Patients with complete (tumour regression grade, TRG=1) or near complete (TRG=2) regression were defined as responders (pR+), while patients with moderate to no regression (TRG>2) were considered as non-responders (pR-). After image acquisition, tumours were semi-automatically segmented and texture analysis was implemented on segmented tumours, computing a) first-order parameters: median, mean and percentiles, b) SUV, metabolic volume and glycolytic volume, and c) 22 second-order texture parameters, derived from Haralick analysis. To identify the features most correlated with TRG, a multivariate logistic regression was performed.
**Results:** After total mesorectal excision, 26 patients were classified as pR-, and 21 as pR+. The multivariate regression model reached statistical significance (p=0.0001), using four texture parameters. The area under the ROC curve was 0.873 (95%CI=0.742-0.953), and sensitivity and specificity in detecting responders were 90% and 77%, respectively.
**Conclusion:** Texture analysis on PET acquisitions could help clinicians in early predict tumour response to neoadjuvant treatment in locally advanced rectal cancer patients. These results could be used to personalize the oncological pathway for patients, delaying or advancing surgery, according to the prediction of treatment response.

### *SS 2.10*

### Characteristics of non-metastatic lymph nodes of rectal cancer: high-resolution MR and fast spin-echo T2-weighted imaging with fat saturation

J. Zhu, X. Wang, J. Gao; Beijing/CN

**Purpose:** To probe the feature of non-metastatic lymph nodes of rectal cancer with high-resolution (HR) MR and fast spin-echo T2-weighted imaging with fat saturation (FSE T2WI/FS) to prevent unnecessary neoadjuvant therapy.
**Material and methods:** 35 patients of rectal cancer underwent HR MR imaging and FSE T2WI/FS. The following attributes were used to describe all of the MR-detected regional lymph nodes: 1) location (above/at/under the level of rectal tumor); 2) diameter; 3) chemical shift effect (CSE) on HR MRI; 4) lymph nodes-to-rectal tumor signal intensity (SI) ratio on FSE T2WI/FS. The characteristics of non-metastatic and metastatic lymph nodes proved by postoperative pathology were compared.
**Results:** A total of 286 non-metastatic and 42 metastatic nodes were studied. 1) There was no predominant location of non-metastatic nodes (P>0.05). 2) The mean diameters of non-metastatic and metastatic nodes were 5mm and 10mm. 3) 75.2% of non-metastatic nodes were detected with CSE, whereas only 14.1% of metastatic nodes were detected with CSE. Area under the ROC curve to assess non-metastatic nodes was 0.89. 4) Lymph nodes-to-rectal tumor SI ratio was 2.24 for non-metastatic nodes and 1.06 for metastatic nodes (P=0.002). A threshold value of 2.12 guaranteed distinction with high sensitivity (81.5%) and specificity (91.5%). Area under the ROC curve/sensitivity/specificity to evaluate non-metastatic nodes were 0.92/87%/91.6% when combining the factors of CSE and lymph nodes-to-rectal tumor SI ratio.
**Conclusion:** Combination of CSE and much higher signal intensity of lymph nodes on FSE T2WI/FS might be useful signs to differentiate benign and malignant lymph nodes of rectal tumor.

**11:00 - 12:30** **Room Bologna**

### *Scientific Session SS 3*
### Advances in abdominal oncologic imaging

### *SS 3.1*
**Measuring colorectal tumour hypoxia with oxygen-enhanced and dynamic contrast-enhanced MRI**
D. Prezzi[1], I. Dregely[1], R. Neji[2], J. Stirling[1], S. Jeljeli[1], G. Cook[1], V. Goh[1]; [1]London/UK, [2]Frimley/UK

**Purpose:** To assess the feasibility of tumour hypoxia measurement in patients with primary colorectal cancer, using oxygen-enhanced blood oxygenation level dependent (OE-BOLD), tissue oxygenation level-dependent (OE-TOLD) and dynamic contrast-enhanced (DCE) MRI.
**Material and methods:** Following ethical approval and informed consent, 19 prospective colorectal adenocarcinoma patients (13 males, mean age 60 years) underwent 3T MRI (Siemens Healthcare). In addition to anatomical T2-weighted sequences, OE-BOLD and OE-TOLD sequences were acquired before and during 100% oxygen inhalation, yielding R2* and R1 tumour maps, respectively. DCE MRI was performed following intravenous injection of gadovist, yielding semi-quantitative area under the curve ($AUGC_{60}$) tumour measurements. Changes in tumour R1 and R2* during oxygen challenge (ΔR1 and ΔR2*) were assessed using Wilcoxon signed rank test and correlated with $AUGC_{60}$. Statistical significance was set at 5%.
**Results:** R1 and R2* quantification was unsuccessful in 7/19 patients, mainly due to bulk tumour motion. Significant negative ΔR1, corresponding to severe hypoxia, were found in 2/12 tumours; non-significant ΔR1, corresponding to moderate hypoxia, in 2/12 tumours; significant positive ΔR1, corresponding to normoxia or mild hypoxia, in 8/12 tumours; significant negative ΔR2*, expected in moderate or severe hypoxia, were found in 5 tumours. No significant correlation was found between ΔR1 and ΔR2*. Conversely, $AUGC_{60}$ correlated positively with ΔR1. The 2 tumours with the highest $iAUGC_{60}$ had the largest positive ΔR1. The 2 tumours with the lowest $iAUGC_{60}$ had negative or non-significant ΔR1.
**Conclusion:** Tumour hypoxia measurement with OE-BOLD, OE-TOLD and DCE MRI is feasible but challenging. ΔR1 correlates positively with tumour perfusion on DCE MRI.

### *SS 3.2*
**Clinicopathologic significance of a new imaging classification of intrahepatic cholangiocarcinoma into ductal and parenchymal types**
H. Rhee, M.-J. Kim, Y.N. Park, C. An; Seoul/KR

**Purpose:** To investigate the clinicopathologic significance of a subclassification of mass-forming intrahepatic cholangiocarcinoma (MF-iCCA) into ductal and parenchymal types based on MRI.
**Material and methods:** We enrolled 72 consecutive patients, in whom MF-iCCA was diagnosed on preoperative MRI and surgical resection from January 2000 to March 2013. Two readers independently evaluated MRI findings of adjacent bile duct dilation, periductal tumor spread, and the presence of diffuse dilatation or abnormality of intrahepatic bile duct. MF-iCCAs with none of the aforementioned findings were defined as parenchymal type, and those with one or more findings were defined as ductal type. The enhancement pattern in the arterial phase was also evaluated. Clinical and histopathological findings, as well as post-surgical outcomes, were collected from medical records.
**Results:** Parenchymal type MF-iCCA (21/78, 27%) exhibited significantly lower serum carbohydrate antigen 19-9 (12.8 vs. 173.8 U/mL) and carcinoembryonic antigen (1.7 vs. 4.2 ng/mL), more frequent viral hepatitis (43% vs. 18%), less frequent biliary intraepithelial neoplasia (0% vs. 26%), less frequent perineural invasion (0% vs. 59%) and lymph node metastasis (7% vs. 46%), compared with ductal type (57/78, 73%) ($P < 0.05$ for all). Parenchymal type MF-iCCA showed more frequent arterial hypervascularity ($P = 0.001$) and better overall survival ($P = 0.030$) than ductal type.
**Conclusion:** Subclassification of MF-iCCAs into parenchymal and ductal type may be useful to discriminate clinical and histopathological characteristics and post-surgical outcomes.

### *SS 3.3*
**Oral administration of multi-target tyrosine kinase inhibitors targeting angiogenesis is associated with submucosal fat deposition in the GI tract**
S. Tamir[1], S. Gavrielli[1], C. Abitbol[2], V. Neiman[1], L. Yosef[1], E. Atar[1], A. Zer[1]; [1]Petah Tikva/IL, [2]Beit Shemesh/IL

**Purpose:** We observed abnormal submucosal fat deposition (SMFD) in the GIT in patients on multi-target TKIs (mtTKI) and, therefore, aimed to define this association and evaluate SMFD in patients on various TKIs.
**Material and methods:** Retrospective evaluation of patients who received mtTKIs (pazopanib and sunitinib) and a comparison cohort of patients on single-target EGFR TKIs (stTKI). All consecutive patients (April 2011-September 2018) with available CT scans before and during therapy were included. An abdominal radiologist blinded to the drug group scored submucosal fat (SMF) as either 0=none, 1=mild (<2mm layer), or 2=moderate-severe (>2mm layer), for each part of the GIT (esophagus, stomach, duodenum, jejunum, ileum, terminal ileum, right colon, left colon). The scores were summed to calculate SMF index (SMFI) for each study. Baseline SMFI before treatment was subtracted from SMFI in each study during treatment, and maximal increase was documented.
**Results:** 40 patients on mtTKI and 23 patients on stTKI were included. Maximal increase in SMFI during treatment was 0 in fifty patients (79%), 1 in six patients (10%), and 3-6 in seven patients (11%). All patients with SMFI increase ≥3 received mtTKIs, with a 17.5% incidence as compared to 0% in the EGRF stTKI group ($p=0.04$). SMFI increase ≥3 was associated with complaints of abdominal pain and/or diarrhea in 5/7 patients.
**Conclusion:** Submucosal fat deposition in the GIT occurs in a significant proportion (17.5%) of patients on multi-target TKIs and is associated with abdominal symptoms. This phenomenon may be unique to multi-target TKIs, as it was not found in patients on single-target TKIs.

### *SS 3.4*
**CT-based nomogram for predicting survival in patients with resectable gallbladder: a retrospective multicenter analysis**
S.-Y. Choi[1], J.H. Kim[2], J.E. Lee[1]; [1]Bucheon/KR, [2]Seoul/KR

**Purpose:** To establish a prognostic nomogram for patients who underwent attempted curative resection for gallbladder cancer, using preoperative CT.
**Material and methods:** Data on 151 consecutive patients (M:F=64:87, 73.26years) with gallbladder cancer underwent CT and subsequent surgery with margin negative resection were retrospectively collected from 2010 to 2018 at two tertiary institutions. The demographic and radiologic parameters were analyzed using univariate and multivariate Cox regression analyses to select independent prognostic factors. The final CT-based nomogram was established using the concordance indices and calibration curves for the 1-, 3-, and 5-year survival probabilities were obtained. Univariate and multivariate survival analyses were performed to select independent prognostic factors. On the basis of the prognostic factors, a nomogram-based predictive model, tumor morphology, bile duct invasion, duodenum invasion and regional LN metastasis were established for the prognosis after curative resection of gallbladder cancer.
**Results:** According to the multivariate model, old age (years) (HR=1.02, $p=0.245$), male (HR=1.38, $p=0.236$), mass-forming type (HR=5.62, $p=0.001$), bile duct invasion (HR=2.99, $p=0.005$), duodenum invasion (HR=2.81, $p=0.027$) and regional LN metastasis (HR=1.92, $p=0.031$) on CT were independent predictors of poor survival and used for constructing nomogram. The nomogram showed good probabilities of survival on calibration curves and the concordance index of the model in predicting OS was 0.745.
**Conclusion:** Preoperative CT findings could predict the prognosis of gallbladder cancer and the CT-based nomogram accurately predicted OS in patients with gallbladder cancer after attempted curative resection.

### SS 3.5

*Withdrawn by the authors*

### SS 3.6

### Diagnostic accuracy for metastatic disease in newly diagnosed colorectal cancer: prospective comparison of whole-body MRI with standard staging pathways - the "Streamline C" trial

S.A. Taylor[1], S. Mallett[2], L. Quinn[2], S. Halligan[1]; [1]London/UK, [2]Birmingham/UK

**Purpose:** Whole-body (WB)-MRI may be an alternative to multi-modality staging of colorectal cancer but its relative diagnostic accuracy, effect on staging times, test number, and impact on treatment decisions are unknown. We undertook a prospective multicentre cohort study to address this.
**Material and methods:** We recruited from 16 hospitals. Eligible patients were 18 years or older, with newly diagnosed colorectal cancer. Patients underwent WB-MRI, the result of which was withheld until standard staging investigations were complete and the first treatment decision was made. The multi-disciplinary team (MDT) recorded its treatment decision based on standard investigations, then on the WB-MRI staging pathway (WB-MRI plus additional tests generated), and finally on all tests. The primary outcome was difference in per-patient sensitivity for metastases between standard and WB-MRI staging pathways against a consensus reference standard at 12 months (paired proportions). Differences in treatment decisions, staging time, and test number were secondary outcomes.
**Results:** 299 patients completed the trial; 68 (23%) had metastasis at baseline. The WB-MRI pathway sensitivity was 67% (95% CI 56 to 78), not significantly different from standard pathways (63% [51 to 74]), p=0·508. Specificity was not significantly different (95% [92 to 97], vs. 93% [90 to 96]). Agreement with the MDT final treatment decision was 96% and 95% for WB-MRI and standard pathways, respectively. Time to complete staging was significantly shorter for WB-MRI. WB-MRI pathways required significantly fewer tests.
**Conclusion:** WB-MRI staging pathways are as accurate as standard pathways, but reduce tests, staging time, and cost.

### SS 3.7

### Structured report for HCC: how we do it?

A. Perez Girbes[1], A. Torregrosa Andrés[1], D.J. Ribeiro Castro Roriz[2], J.P. Azorin Vicente[1], P. Rudenko[1], A. Batista Domenech[1], L. Marti-Bonmati[1]; [1]Valencia/ES, [2]Coimbra/PT

**Purpose:** To describe the process of developing a structured report tool in our center reporting MRI in patients at risk for HCC.
**Material and methods:** First, a preliminary list with relevant questions was presented at the liver disease multidisciplinary team (MDT) meeting. After discussion, a final list of questions and answers was accepted by consensus. We agreed to use the Liver Imaging Reporting and Data System (LI-RADS) v2018 lexicon for reporting the MRI findings. Bioimaging engineers developed a web-based platform using Angular® with a Mongo Database to save data. All data were stored in an ancillary server located at the hospital.
**Results:** The developed structured report tool includes mainly a customized checkbox for each item. Following sections were included: 1) clinical data (age, gender, Child-Pugh, model for end-stage liver disease (MELD), performance status test, etiology of chronic liver disease); 2) technical MRI aspects (magnetic field strength, UKCA used, quality of study); 3) chronic liver disease findings (morphologic cirrhosis changes, steatosis, inflammatory changes, iron deposit, fibrosis and portal hypertension findings); 4a) non-treated focal liver lesions (major and minor criteria based in LI-RADS v2018); 4b) treated focal liver lesion; 5) other findings not related to liver disease. After completing all items, the tool offers a final report with LI-RADS category of each lesion and suggests an EASL staging and treatment to start discussion at MDT meeting. The tool was easily accepted by radiologists and MDT members.
**Conclusion:** The model of structured report tool for HCC based on LI-RADS standard lexicon and EASL staging system facilitates communication and research within MDT.

### SS 3.8

### Visceral obesity and colorectal cancer: is there a relationship between them? First CT-based volumetric study

M. Ersen, S. Akay, M. Urkan, U. Balyemez, M. Tasar; Ankara/TR

**Purpose:** To examine the possible relationship between abdominal adiposity parameters and the presence of colorectal cancer (CRC) and between these adiposity parameters and various histopathological findings of the tumor.
**Material and methods:** 60 control subjects and 111 patients with colorectal cancer (CRC), 63 with early-stage and 48 with advanced-stage disease, were enrolled. Medical data and abdominopelvic CT examinations of each study group were retrospectively reviewed. Abdominal adiposity parameters, including visceral adipose tissue (VAT) volume, subcutaneous adipose tissue (SAT) volume, and total adipose tissue (TAT) volume, were calculated on all slices of the CT examinations with specialized software, and results of each study group were compared. Adiposity parameters were also compared with tumor histopathologic findings.
**Results:** We found lower VAT and higher SAT volumes in advanced-stage CRC patients, compared to the early-stage group. However, this relationship was not statistically significant (p = 0.721 for VAT and p =0.432 for SAT volumes). We detected significantly lower VAT and SAT volumes in the early-stage CRC group, when compared with the control group (p = 0.014 for both). There was no significant relationship between TAT volumes and the study groups (p = 0.06). No statistically significant relationship was detected between adipose tissue parameters and histopathological features of the CRC group (p > 0.05).
**Conclusion:** We found statistically significant lower VAT and SAT volumes in patients with early-stage CRC compared to the control group. Volumetric adipose tissue measurements may be more accurate than area measurements and can easily be performed on abdominopelvic CT, which is the routine imaging modality for CRC patients.

### SS 3.9

### Correlation between apparent diffusion coefficient value on MRI and histopathologic World Health Organization grades of neuroendocrine tumors

W. Mebis, B. Corthouts, H. El Addouli, S. Nicolay, A. Snoeckx, M.J. Spinhoven, A. Van Hoyweghen, B. Op De Beeck; Edegem/BE

**Purpose:** To investigate the possible correlation between apparent diffusion coefficient (ADC) value on MRI and histopathologic World Health Organization (WHO) grades of neuroendocrine tumors (NET).
**Material and methods:** Electronic patient records from patients presented at the multidisciplinary NET board between November 2017 and December 2018 were retrospectively reviewed. Patients with both available MRI (primary tumor or metastasis) and known WHO tumor grade were included (n=46). Analysis was performed by a radiology resident under the supervision of a senior radiologist. Average and minimum ADC values (avgADC; minADC) were measured by drawing a freehand region of interest (ROI) excluding only the outermost border of the lesion. The largest axial size (primary tumor) or most clearly delineated lesion (metastasis) was used with the post-contrast T1 images as a side by side reference.
**Results:** 46 patients met the inclusion criteria (age 30–88; 23F/23M). 21 patients (46%) were diagnosed with WHO G1 tumor, 17 with G2 (37%) and 8 with G3 (17%) tumor. Primary tumor location was pancreas (n=29), small intestine (n=6), rectum (n=3), stomach (n=2), lung (n=2), colon (n=1) and unknown (n=3). A significant difference was found between low-grade (G1+G2) and high-grade (G3) tumors (Mann-Whitney; avgADC: p<0.001; minADC: p=0.001). There was a moderate negative correlation between WHO grade and avgADC/minADC (Spearman; avgADC: -0.601; 95% CI [-0.770; -0.371]; minADC: -0.586; 95% CI [-0.758; -0.355]).
**Conclusion:** Our data show a significant difference in both average and minimum ADC values on MRI between low (G1+G2)- and high (G3)-grade NET. A moderate negative correlation was found between ADC value and histopathologic WHO grade.

### *SS 3.10*
### Imaging evaluation of liver tumour burden in metastatic pancreatic neuroendocrine tumours
G. Rizzo, A. Beleù, A. Sarno, N. Cardobi, A. Giaretta, A. Grecchi, I. Testa, R. De Robertis, M. D'Onofrio; Verona/IT

**Purpose:** Half of pancreatic neuroendocrine tumors (PNET) show metastases at diagnosis, with liver involvement in 28-77% of cases. The aim of the study is to evaluate the relationship of radiological qualitative/quantitative descriptors of liver tumour burden (LTB) and liver metastases distribution pattern (LMDP), with fludeoxyglucose (18FDG) positron emission tomography (PET-CT) findings and histological grading (HG).
**Material and methods:** LTB of 47 patients with metastatic PNET were retrospectively evaluated, before any treatment intervention, on contrast-enhanced computed tomography and/or magnetic resonance. Two skilled radiologists independently assessed LMDP (unifocal, paucinodular, multinodular and bulky), qualitative and quantitative descriptors of LTB. Accuracy in assessing positive PET-CT and HG were calculated. Cohen's K-coefficient was calculated to measure inter-observer agreement (IOA).
**Results:** The most accurate parameters in assessing PET-CT positivity were late-phase hypodensity (88.9%), venous-phase hypodensity (87.2%), arterial-phase hyperdensity (83.7%), major-axis sum >5 cm (74.5%) and >10 cm (61.7%), arterial-phase inhomogeneity (69.8%) and metastases number >5 (61.7%). In assessing HG only arterial-phase hypodensity (76.7%) and infiltrating margins (71.8%) resulted to be accurate. Multinodular LMDP was the most common pattern reported. A confluent pattern was reported only in aggressive tumours with Ki67 >5%. IOA was higher when evaluating major-axis sum >10 cm (0.96) and >5 cm (0.76), arterial-phase homogeneity (0.83) and arterial-phase hyperdensity (0.63), but lower when evaluating localization (0.51) and late-phase hypodensity (0.36).
**Conclusion:** LTB predicts functional and anatomopathological characteristics of PNETs. Arterial-phase hyperdensity, major-axis sum >5 cm are accurate in evaluating functional characteristics of the tumour, while arterial-phase hypodensity relies on HG. Confluent pattern relies on more aggressive neoplasms with Ki67 >5%.

**11:00 - 12:30** **Room Amalfi**

### *Scientific Session SS 4*
### The acute abdomen: diagnosis and intervention

### *SS 4.1*
### Cost-effectiveness of imaging in the assessment of appendicitis
C. Ivan, A. Al-Nowfal, S.R. Hudson, A. Osman, R. Verma, J.A. Stephenson; Leicester/UK

**Purpose:** Studies have found a decrease in negative laparotomy and appendiceal perforation rate when CT imaging was used in selected patients with suspected appendicitis. The aim of our study was to determine the cost-effectiveness of performing diagnostic imaging as part of the preoperative assessment of patients with right iliac fossa pain (RIF).
**Material and methods:** We have developed a local pathway for patients with RIF pain. On this pathway, if the examination is suggestive of appendicitis, and the C-reactive protein >10mg/L or white cell count >10.5 x $10^3$/uL, then an US focussed on the assessment of the appendix is performed. If this is equivocal, the patient automatically has a low-dose CT abdomen and pelvis. We have calculated the cost of managing 1500 patients pre-pathway and 1500 post-pathway.
**Results:** After introducing imaging assessment in this patient group, the negative appendicectomy rate decreased to <4% (from 36%) in our institution. The cost of an US scan is £32, of a CT scan is £118 with a laparoscopic appendicectomy costing £2358 (if complicated £3494, rate of complicated appendicitis/complications 10-20%). Pre-pathway, we performed 800 US scans, 100 CT scans and 825 laparoscopies (of which 82 were complex), resulting in a cost of £2,075,902. During the pathway, 1500 US scans, 1000 CT scans and 450 laparoscopies (45 complex) were performed, resulting in a cost of £1,278,220.
**Conclusion:** Introducing an imaging pathway for patients with suspected appendicitis has saved our institution ~£800,000 per annum and has significantly reduced the negative appendicectomy rates.

### *SS 4.2*
### Non-strangulated adhesive small bowel obstruction: CT findings predicting failure of non-surgical treatment
Y. Lee, J.E. Kim, J.-H. Yoon, S.H. Kim; Busan/KR

**Purpose:** To identify CT findings that predict failure of non-surgical treatment in patients with non-strangulated adhesive small bowel obstruction (SBO).
**Material and methods:** This retrospective study was approved by our institutional review board, and informed consent was waived. Contrast-enhanced CT studies in 189 patients with adhesive SBO that was initially treated conservatively were reviewed independently by two radiologists to identify CT findings predicting failure of non-surgical treatment. The findings included the location of transition zone, number of beak sign, maximum diameter of bowel dilatation, C-loop, fecal sign, whirl sign, bowel wall thickening, mesenteric haziness, amount of mesenteric or peritoneal fluid and submucosal edema. These findings were statistically compared according to the success or failure of non-surgical treatment.
**Results:** Nonsurgical treatment succeeded in 144 patients (76.2%) and failed in 45 patients (23.8%). At univariate analysis, anterior location of adhesion and a fecal sign were associated with success of non-surgical treatment, whereas two beak signs or more, the presence of c-loop, mesenteric haziness and moderate amount of mesenteric fluid were associated with failure of non-surgical treatment. At multivariate analysis, lack of fecal sign, mesenteric haziness and moderate amount of mesenteric fluid were independent findings predicting failure of non-surgical treatment, with odds ratios of 5.08, 7.77 and 6.74, respectively.
**Conclusion:** The lack of fecal sign, mesenteric haziness and moderate amount of mesenteric fluid are useful indicators to predict failure of non-surgical treatment in patients with non-strangulated adhesive SBO.

### SS 4.3

### Bowel infarction from acute mesenteric ischemia and strangulated small bowel obstruction: distinct CT features

P. Calame, A. Malakhia, F. Grillet, E. Delabrousse; Besançon/FR

**Purpose:** To assess if transmural bowel necrosis (TBN) shows distinct CT features according to the three main etiologies, i.e. occlusive acute mesenteric ischemia (O-AMI), non-occlusive AMI (NO-AMI) and strangulated small bowel obstruction (SBO).
**Material and methods:** The local review board approved this retrospective study. From January 2010 to December 2017, all patients with a diagnosis of TBN at pathology were extracted from the pathology department database of our University Hospital. The inclusion criteria of our study were 1) the presence of TBN on pathology examination and 2) available contrast-enhanced CT performed within the 24 hours prior to the pathological examination. 77 patients were finally included. The CT studies were retrospectively reviewed by 2 independent abdominal radiologists to identify the classic CT findings of TBN.
**Results:** Pneumatosis intestinalis was statistically more frequent in NO-AMI than in O-AMI and strangulated SBO (66% vs 28% vs 7%; p<0.01) as well as mesenteric venous gas (57% vs 29% vs 0%; p< 0.01) and portal gas (52% vs 10% vs 0%; p<0.01). Decreased or absent bowel wall enhancement was more frequent in AMI groups than in SBO (83,81% in Non-occlusive, occlusive AMI and 59% in SBO, p=0.02). Increased unenhanced bowel wall attenuation was more frequent in strangulated SBO (39%) than in the two AMI groups (10% and 19% in NO-AMI and O-AMI, respectively, p<0,01).
**Conclusion:** TBN shows distinct CT findings according to its three main etiologies. In our study, no portal gas has been found in strangulated SBO.

### SS 4.4

### CT in occlusive and non-occlusive ischemic colitis

N.S. Gonzalez[1], V. Plodeck[1], J. Dobroschke[1], M. Wulf[2], M. Laniado[1]; [1]Dresden/DE, [2]Buenos Aires/AR

**Purpose:** To evaluate CT findings of ischemic colitis (IC) and to correlate findings with the etiology (occlusive/OIC vs non-occlusive/NOIC).
**Material and methods:** We retrospectively evaluated 60 patients with IC confirmed by surgery and/or endoscopy. CT and clinical history were reviewed. CT signs of IC (pericolic fat stranding/fluid, colonic wall thickening/thinning/enhancement, bowel dilatation, intraabdominal free air/wall pneumatosis, and/or mesenteric vascular stenosis/occlusion) and its localization were analyzed and correlated with the presence of OIC and NOIC. The association between CT findings and OIC vs NOIC was statistically analyzed and compared using Pearson‘s chi square.
**Results:** There were 41 male and 19 female patients (mean age 70 years). NOIC was found in 38 patients and OIC in 22. All patients showed pericolic fat stranding and/or fluid. Wall thinning was found in 34 patients (57%) and wall thickening in 26 (43%). Twenty-six patients (43.3%) showed abnormal wall enhancement, and 19 (31.6%) presented with wall pneumatosis. Segmental involvement was more frequent (n=38/63%) than generalized manifestation. A significant association was found between NOIC and wall thinning and dilatation (p<0.001), right colonic involvement (p=0.008), and intraabdominal free air (p=0.005). OIC was significantly associated with wall thickening (p<0.001). Forty-six patients showed mesenteric abnormalities (77%). Overall mortality was 46%. Patients were at greater risk with right and subtotal colonic involvement compared to left colonic involvement (p=0.010).
**Conclusion:** NOIC is more frequent than OIC. NOIC usually shows wall thinning with dilatation. OIC frequently presents with wall thickening. Knowledge of risk factors and the spectrum of CT signs are essential for the diagnosis of IC, a potentially fatal disease.

### SS 4.5

### Comparison of contrast-enhanced US and MDCT with angiography: enhanced assessment of bleeding in blunt abdominal trauma

J. Lee[1], Y. Kim[1], J.Y. Moon[2], K.S. Park[1]; [1]Cheongju/KR, [2]Seoul/KR

**Purpose:** To compare bleeding findings of patients with blunt abdominal trauma in contrast-enhanced ultrasound (CEUS) and contrast-enhanced MDCT with angiography as a gold standard.
**Material and methods:** Twenty-six patients with 28 lesions of blunt abdominal trauma who underwent CEUS, MDCT, and angiography were enrolled in this retrospective study. Injuries included liver (n=14), kidney (n=5), and spleen (n=9). Extravasation of contrast agent was classified into three types: (a) isolated pseudoaneurysm; (b) pseudoaneurysm with low-velocity extravasation; and (c) active extravasation. Sensitivity, specificity, positive predictive value (PPV), negative predictive value (NPV), and accuracy of CEUS for the detection and classification of bleeding were calculated based on angiography and compared to MDCT using McNemar test.
**Results:** All 24 extravasations of contrast agent were detected in both CEUS and MDCT and other 4 lesions without bleeding were equally observed in CEUS and MDCT. Bleedings were classified into 4 isolated pseudoaneurysms, 8 pseudoaneurysms with low-velocity extravasation, and 12 active extravasations based on angiography. Sensitivity, specificity, PPV, NPV, and accuracy for assessment of bleeding were 87.5%, 100.0%, 100.0%, 57.1%, and 89.3%, respectively, for CEUS and 86.4%, 66.7%, 90.5%, 57.1%, and 82.1%, respectively, for MDCT. P value of comparison for two modalities was 0.62, and difference in each test was not statistically significant.
**Conclusion:** CEUS and MDCT exhibited high consistency with angiography for both detection and classification of bleeding in patients with blunt abdominal trauma. Therefore, it could be possible to perform CEUS as the first-line approach to determine appropriate management in blunt abdominal trauma.

### SS 4.6

### Inflammation-related radiological findings of gallbladder adenomyomatosis

W.-S. Chung, J.Y. Kim, H.J. Lee, J. An; Daejeon/KR

**Purpose:** A relationship between gallbladder (UK) adenomyomatosis and early-stage UK cancer remains unclear, while cholecystitis secondary to adenomyomatosis may lead to dysplastic changes and cancer. The purpose of this study was to assess radiological findings for differentiating adenomyomatosis without chronic cholecystitis from with chronic cholecystitis.
**Material and methods:** We retrospectively identified cases of 80 patients with pathologically proven adenomyomatosis without chronic cholecystitis (n=7) or with chronic cholecystitis (n=73) who underwent CT and MRI followed by surgery. Analysis of the MR findings included evaluation of UK wall thickening type, the presence of intramural cyst, location of intramural cyst, and the presence of stone. CT finding included UK wall thickening type. A multivariate logistic regression model was constructed to identify the image-based findings of adenomyomatosis associated with chronic cholecystitis.
**Results:** MR-based UK wall thickening type, the presence of stone, and CT-based UK wall thickening type were significantly different between adenomyomatosis without chronic cholecystitis and with chronic cholecystitis. On multivariate analysis, the presence of stone was only identified as the significant predictor of adenomyomatosis with chronic cholecystitis (odds ratio: 14.709; 95% CI: 1.487–145.491; p = 0.021). There was no significant difference in other MRI- and CT-based findings between adenomyomatosis without chronic cholecystitis and with chronic cholecystitis.
**Conclusion:** In patients with adenomyomatosis, the presence of stone was independently associated with chronic cholecystitis.

### SS 4.7

### Diagnostic accuracy of unenhanced CT in acute abdominal pain

U. Russo, V. Murelli, R. Cobelli, G. Pedrazzi, A.A. Palumbo; Parma/IT

**Purpose:** To evaluate the diagnostic accuracy of unenhanced CT scan in acute abdominal pain and estimate the incremental diagnostic value of contrast medium injection in determining the final diagnosis.
**Material and methods:** We retrospectively evaluated CT scans of 146 patients that acceded our emergency department with acute (< 3 days) abdominal pain. Two radiologists (1 expert and 1 young) independently and blindly evaluated both unenhanced and enhanced (venous phase) CT scans, and for each of them they were asked to formulate a final diagnosis. These diagnoses were classified into 14 categories and used for calculating intra-observer agreement (Cohen's kappa), diagnostic accuracy, sensitivity, specificity, positive and negative predictive value.
**Results:** Intra-observer agreement between unenhanced and enhanced scans was approximately 0.8 (good/very good) both for the expert and the young radiologist. Diagnostic accuracy increased from 78.8% to 83.6% after contrast injection; however, if sensitivity increased from 78.4% to 90.7%, specificity unexpectedly decreased from 79.4% to 71.8%. Contrast medium incremental diagnostic value, defined as the percentage of cases in which the correct diagnosis was identified only on enhanced scans, was 11.6% for the expert radiologist but only 6.8% for the young. Predictive value stratified on the differential diagnosis was very variable. Diagnoses with the best predictive value (> 90%) were diverticulitis, ileus, cholecystitis, pancreatitis, obstructive nephropathy and muscular hematoma.
**Conclusion:** The level of agreement between unenhanced and enhanced scan diagnoses is high. In selected cases when the diagnosis on unenhanced scan matches the diagnostic suspect, contrast medium injection may not be necessary.

### SS 4.8

### Clinical impact of iterative model reconstruction algorithm in the assessment of abdominal disease in a department of emergency radiology: a large series of patients

A. Pecorelli, C. Maino, C. Talei Franzesi, L. Riva, A. Devito, T. Giandola, S. Sironi, D. Ippolito; Monza/IT

**Purpose:** To compare the radiation dose exposure of low-dose CT examinations using iterative model reconstruction (IMR) algorithm with that of standard CT equipment in an emergency radiology department (ERD).
**Material and methods:** We reviewed more than 7000 CT scan examinations performed with new 256 row CT scan (iCT Elite, Philips) combined with new IMR algorithm, in the abdominal district in patients referred to our ERD with different clinical emergency settings (trauma, abdominal pain, diverticulitis, etc). A control group of 5500 patients underwent standard dose abdominal CT protocol (100 kV; automated mAs) on 256 row CT scanner. For each exam, we analyzed the CT dose index (CTDI, expressed in mGray) and the dose length product (DLP, expressed in mGray.cm) and compared with the dose of our previous CT scan equipment.
**Results:** The mean values of CTDI were lower with IMR compared with our previous CT equipment: abdomen 17.7 vs 6.3 (-64%) and whole body 16.8 vs 12.4 (-26%). In the same way, the total DLP were lower with IMR compared with standard CT: abdomen 911.0 vs 361.5 (-60%) and whole body 1118.0 vs 893.0 (-20%). The subjective image quality of low kV IMR-abdominal study was also higher compared with standard dose CT studies.
**Conclusion:** Low-dose CT (80 kV and automated mAs modulation) abdominal study reconstructed with model-based algorithm represents a feasible protocol for the evaluation of abdominal disease in the emergency setting, achieving high image quality with extremely low noise and a significant dose reduction within adequate reconstruction times (less than 120 seconds).

### SS 4.9

### Endovascular treatment of abdominal visceral artery aneurysms and pseudoaneurysms: extravascular stent graft migration as a possible long-term complication

M.A. Tipaldi, F. Laurino, A. Pisano, G. Orgera, A. Laghi, M. Rossi; Rome/IT

**Purpose:** Immediate- and long-term result analyses of endovascular visceral aneurysm and pseudoaneurysm treatment with stent grafts.
**Material and methods:** From February 2006 to October 2011, we deployed 23 stent grafts in 21 patients (12 males and 9 females, mean age 58.2 years) affected by aneurysm or pseudoaneurysm of the splenic artery (n=13), hepatic artery (n=5), gastroduodenal artery (n=1) and renal artery (n=2). The aneurysmal sack dimensions ranged from 9 to 60 mm. We evaluated the technical success, stent patency, possible migration, breakage and aneurysmal reperfusion. The follow-up, performed with CECT, was repeated at 1, 6 and 12 months, then yearly (range 12-72 months).
**Results:** We achieved complete aneurysmal exclusion in 20/21 patients (95%). In one case, we had incomplete sealing and splenic artery's hilar branch fissuration during the procedure and thus treated with coils and ruled out of the follow-up. We reported complete aneurysmal exclusion in 20/20 patients at follow-up. In 4 cases, we observed stent occlusion, although without organ-related ischemia; in 3 of these 4 cases, stent occlusion was associated with stent graft migration: one inside the gastric antrum at the 36th month during follow-up (confirmed with endoscopy) and two in the lesser omentum at the 12- and 60-month follow-up.
**Conclusion:** Endovascular treatment of visceral artery aneurysms and pseudoaneurysms with stent grafts is a safe and effective technique with good immediate- and long-term results. Extravascular migration of the stent graft after its occlusion is a possible late complication. According to our evidence, we recommend follow-up even in case the stent is occluded.

### SS 4.10

### Endovascular management of hepatic artery thrombosis on the first post-operative day after living donor liver transplantation

O. Abdelaziz, S. Emadeldin; Cairo/EG

**Purpose:** Our aim was to asses the feasibility and potential complications of endovascular intervention for the management of early hepatic artery thrombosis (HAT) on the first post-operative day after living-donor liver transplantation (LDLT).
**Material and methods:** This is a retrospective review of 668 recipients who underwent LDLT between August 2001 and August 2016 at 3 centers. Endovascular interventions were performed using standard catheter techniques. Thrombolysis was performed using tPA or streptokinase, whereas angioplasty and stent placement were performed if there was an underlying stricture.
**Results:** Early HAT within 2 weeks postoperatively occurred in 30 patients (4.5%). Endovascular interventions were performed for 26 patients with initial success in 22/26 (84.6%) and failure in 4/26 (15.4%). Rebound thrombosis developed in 4/22 (18.2%). Complications occurred in 8/26 (30.7%): bleeding (4/26), anastomotic rupture (1/26), and dissection (3/26). The overall definite endovascular treatment success rate of HAT was 18/26 (69.2%), whereas that of definite surgical/endovascular or combined treatment was 26/30 (86.6%). HAT occurred on the first postoperative day in 8/30 patients. Endovascular intervention was attempted in 4 patients and succeeded in 3 patients (75%). Surgery was attempted in 4 patients and succeeded in 3 patients (75%). Endovascular complications occurred in 2/4 patients (50%), bleeding occurred in 1 patient, and anastomotic rupture occurred in 1 patient.
**Conclusion:** Endovascular intervention for the management of HAT on the first postoperative day after LDLT carries a considerable risk of potential complications related to the technique and thrombolytic therapy. However, it is feasible and can be attempted for graft salvage if surgery is considered futile.

**11:00 - 12:30** **Room Milan**

*Scientific Session SS 5*

### Liver interventional radiology and assessment of treatment response

### SS 5.1

### Predictive technetium-99 m macroaggregated albumin-single-photon emission computed tomography/CT dosimetry in patients with locally advanced inoperable HCC treated by selective internal radiotherapy with 90Y resin microspheres

A.-L. Hermann[1], A. Dieudonne[2], M. Ronot[2], M. Sanchez[2], H. Pereira[1], G. Chatellier[1], E. Garin[3], L. Castera[2], R. Lebtahi[2], V. Vilgrain[2]; [1]Paris/FR, [2]Clichy/FR, [3]Rennes/FR

**Purpose:** To predict survival and tumor response in patients with locally advanced inoperable HCC treated by selective internal radiation therapy (SIRT; yttrium-90 [$^{90}$Y] resin microspheres) using tumor-absorbed dose calculated before therapy.

**Material and methods:** Among 184 patients from the SARAH trial who received SIRT, 121 and 109 were included in dose/survival and dose/tumor response analyses, respectively; CT, technetium-99 m macroaggregated albumin -single-photon emission tomography/CT ($^{99m}$Tc-MAA-SPECT/CT) and $^{90}$YSPECT/PET were centralized. Tumor-absorbed dose was computed using $^{99m}$Tc-MAA-SPECT/CT. Visual agreement between CT-MAA-$^{90}$Y (optimal, suboptimal, not optimal), overall survival (OS) and tumor response at 6-month follow-up CT (RECIST 1.1) was assessed.

**Results:** In the dose/survival population, median OS was 9.3 months (95%CI, 6.7–10.7) and median tumor-absorbed dose was 112 Gy (IQR, 67.8–220). Patients who received ≥100 Gy (n=67) had significantly longer survival than patients who received <100 Gy (median 14.1 months; 95%CI, 9.6–18.6 vs 6.1 months 95%CI, 4.9–6.8, respectively; P< .0001), and those with optimal agreement (n=24) had the longest median OS (24.9 months; 95%CI, 9.6–33.9). In the dose/tumor response population, the proportion of treatments leading to objective response (OR) and disease control (DC) was 20.5% and 63.7%, respectively. Tumor-absorbed dose was significantly higher in treatments leading to DC vs no-DC (median 121 Gy; IQR, 86.0–190 vs 85.1 Gy; IQR, 58.4–164, P= .0204), but was not significant with OR vs no-OR (P= .20). The highest DC rate (77.5%) was observed with tumor-absorbed dose ≥100 Gy and optimal agreement (m=40).

**Conclusion:** Tumor-absorbed dose computed on $^{99m}$Tc-MAA-SPECT/CT is significantly associated with OS and DC; the most benefit is observed with both tumor-absorbed dose ≥100 Gy and optimal visual CT-MAA-$^{90}$Y agreement.

### SS 5.2

### Does the tumoral density heterogeneity after selective internal radiation therapy predict local progression in colorectal hepatic metastases?

A. Saifi, M. Lemort, S.-L. Chao, M.A. Bali, M. Vouche; Brussels/BE

**Purpose:** The purpose of the present study is to investigate whether the appearance of tumor density heterogeneity after selective internal radiation therapy (SIRT) on CT can predict local progression of colorectal cancer liver metastases (CRCLM).

**Material and methods:** After approval of the IRB, forty-five CRCLM among nineteen patients treated with $^{90}$Y-radioembolization and imaged with CT at the portal venous phase performed at baseline and 6-8 weeks after the treatment were retrospectively analyzed using TumourMetrics in-house software. The longest tumor diameter, the volume, the mean density, the standard deviation and the kurtosis of the density of the non-enhancing and enhancing portions of the 3 largest lesions were recorded at each time point. Adapted RECIST 1.1 criteria were applied to assess treatment response. Non-parametric tests were used to assess differences between responders and non-responders.

**Results:** 36 lesions showed no progression and 9 showed local tumor progression at first or second follow-up. In lesions showing progression, the decrease of the standard deviation of density in the non-enhancing portion of the tumor was more pronounced compared to the responding lesions (-27% vs -15%) (p.=.0.002). The threshold of -17% allows to obtain a sensitivity of 78% and specificity of 89% with an area under the curve of 83% and a Youden index of 67%. No other significant differences were found.

**Conclusion:** The decrease of standard deviation of density in the non-enhancing tumor portion at CT follow-up after SIRT seems to be able to predict local progression.

### SS 5.3

### Yttrium 90-radioembolization in patients with HCC and portal vein invasion: external validation of the Milan prognostic score

P. Scalise, I. Bargellini, E. Bozzi, O. Perrone, G. Boni, R. Cioni; Pisa/IT

**Purpose:** To retrospectively analyse clinical outcomes of a series of patients with HCC and portal vein tumor thrombosis (PVTT) who underwent yttrium-90 (Y90) radioembolization (TARE) and to validate the recently proposed Milan PVTT prognostic score.

**Material and methods:** We retrospectively reviewed the data of all HCC patients with PVTT who underwent TARE between 2012 and 2018. Laboratory, radiological and clinical data were collected. PVTT extension was graded as PV1 (segmentary), PV2 (secondary order branch), PV3 (first-order branch) and PV4 (main portal trunk). Patients were classified according to the Milan PVTT score in three groups: good, intermediate and dismal prognosis. Overall survival (OS) was estimated by Kaplan-Meier method, and compared by log-rank test.

**Results:** The study included 70 HCC patients (90 male; median age 67 years). According to the Milan PVTT score, 15 (21.4%) patients were in the good prognostic group, 33 (47.1%) in the intermediate group and 22 (31.4%) in the dismal group. Median OS was 11.6 months (95%CI, 9.1- 17.5), and one- and three-year survival rates were 47.9% and 11.2%, respectively. The Milan PVTT score was significantly associated with OS, with median OS ranging from 24.6 months in the favourable group, 13 months in the intermediate group (HR=3.2; 95%CI, 1.2-9.7; p=0.016) and 5.9 months in the dismal group (HR=4.1; 95%CI, 1.4-13.4; p=0.0096).

**Conclusion:** Our data support the use of the Milan PVTT score as an easy tool to select HCC patients with PVTT who may benefit from TARE. This prognostic model should also represent the basis for future trials.

### SS 5.4
### Correlation between the dose of radioembolization and liver hypertrophy of the future remnant liver
M. Vouche, Y. Aboufirass, M. Lemort, P. Flamen; Brussels/BE

**Purpose:** The aim of this study was to analyze the correlation between the dose of $^{90}$Yttrium administered by transarterial radioembolization (TARE) in the non-tumoral right lobe volume (NTRLV) and hypertrophy of the future liver remnant (FLR).
**Material and methods:** This study was approved by the local ethics committee review board. All patients treated at our institution by unilobar TARE with available baseline and/or follow-up CT or MRI and post-treatment $^{90}$Yttrium-PET-CT were included. Volumes at baseline, first (6-8 weeks post-treatment) and last available follow-ups were determined on CT/MRI using a contouring software for 18 patients. The percentage hypertrophy of the FLR (%HT FLR) was calculated. The mean administered dose (Gy) to the non-tumoral liver was estimated on $^{90}$Yttrium-PET-CT images. Statistics included non-parametric tests for continuous (Wilcoxon test) and categorical variables (Fisher's exact test), receiver operating characteristic (ROC) curves and correlation statistics (Pearson and Spearman tests), considering a p value < 0.05 as significant.
**Results:** The median %HT FLR was 19.75 (IQR 11-43 ; p=0.02). The right lobe and its respective tumor load decreased in volume (p=0.02). A correlation was found between time and %HT FLR (p= 0.003). Administered dose was correlated to the atrophy of the non-tumoral right lobe volume (NTRLV) (r=-0.67, $r^2$=0.45, p=0.03) and to the hypertrophy of the contralateral lobe (r=0.68, $r^2$=0.46 and p=0.039).
**Conclusion:** A correlation between the dose of $^{90}$Yttrium in the NTRLV and homolateral lobe atrophy as well as contralateral lobe hypertrophy was demonstrated. A time-dependent hypertrophy of the FLR was demonstrated.

### SS 5.5
### Value of diffusion-weighted MRI for response evaluation of hepatic metastases of primary neuroendocrine tumors undergoing selective internal radiotherapy
M.K. Ingenerf, L. Kaiser, H. Karim, H. Ilhan, J. Ricke, C. Schmid-Tannwald; Munich/DE

**Purpose:** To evaluate the role of diffusion-weighted imaging (DWI) in assessing treatment response in patients with liver metastases of primary neuroendocrine tumors (NETs) following selective internal radiotherapy (SIRT) with 90Yttrium-microspheres.
**Material and methods:** 43 patients with liver metastases of primary NET who underwent abdominal MRI with DWI 40 ± 27 days before and 77 ± 48 days after SIRT were included. Tumor size, intralesional minimal, maximal and mean apparent diffusion coefficient (ADCmin, ADCmax and ADCmean, respectively) were measured for maximal 3 target lesions per patient on baseline and post-interventional DWI. Tumor response to radioembolization was categorized according to Response Evaluation Criteria in Solid Tumors v1.1 (RECIST) on follow-up examination.
**Results:** A total of 120 metastases with a mean diameter of 3.04 ±1.59 cm was analyzed. 27 (22.5%) lesions were categorized as partial response (PR), 87 (72.5%) lesions as stable disease (SD) and 6 (5%) lesions as progressive disease (PD). ADC values (ADCmin, max, mean) increased significantly (p<0.005) after SIRT in the group of PR and SD whereas there was no significant change of ADC values in the group of PD. Between the group of PR and SD, there was a significant difference in percentage change of ADCmean (61% vs. 11%, respectively, p<0,05) pre- and post-interventional.
**Conclusion:** ADC values, especially ADCmean changes on DWI, seem to represent a valuable marker for the evaluation of treatment response after radioembolization of hepatic metastases in patients with primary NET and may help in assessing further therapeutic strategies.

### SS 5.6
### Prediction of treatment response following transarterial chemoembolization in patients with HCC using dual-tracer positron emission tomography
W.H.K. Chiu, H. Yuan, J. Van Lunenburg, V. Vardhanabhuti, D. Tse, E.Y.P. Lee; Hong Kong/HK

**Purpose:** While dual-tracer positron emission tomography (DT-PET) with 18F-FDG and 11C-acetate is highly sensitive in detecting HCC, its ability to prognosticate outcomes has not been previously explored. This study aims to evaluate whether DT-PET can predict treatment response in patients with HCC undergoing transarterial chemoembolization (TACE).
**Material and methods:** Patients with HCC who had undergone DT-PET prior to TACE were retrospectively identified. Patient demographics, radiotracer uptake patterns, treatment response as per modified Response Evaluation Criteria In Solid Tumours (mRECIST), progression-free (PFS) and overall (OS) survival were analysed.
**Results:** Between 2015 and 2018, 28 patients (M:F 22:6, median age 64) with 38 target lesions were included. Ten patients had multifocal while 18 had a solitary HCC (median size 19mm). 18F-FDG and 11C-acetate avidities were seen in 37% and 79% of HCC, respectively. Patients who responded to treatment had significantly longer OS (median 47.3 vs 20.9 months, p=0.022) while FDG uptake was associated with shorter PFS (median 2.7 vs 20.8 months, p=0.013) and OS (median 22.3 vs 44.7 months, p=0.008). Baseline raised AFP and multifocal disease were also associated with poorer OS. Lesion-based analysis showed acetate uptake (defined as tumour-to-liver ratio <2 for low or >2 for high) was predictive of treatment response (78% vs 47%, p=0.045).
**Conclusion:** Our study shows that HCC with high acetate uptake is associated with less favourable response to TACE on a per lesion basis while FDG uptake in HCC is associated with shorter PFS and OS. DT-PET can potentially be a novel prognostic biomarker in the era of precision medicine.

### SS 5.7
### CT texture analyses of colorectal liver metastases before and after thermal ablation can predict local tumour progression
F. Staal[1], M. Taghavi[1], F. Imani[1], F. Gómez Muñoz[1], E. Klompenhouwer[1], D. Meek[2], M. De Boer[1], D. Lambregts[1], R.G.H. Beets-Tan[1], M. Maas[1]; [1]Amsterdam/NL, [2]Capelle aan den IJssel/NL

**Purpose:** To investigate whether CT texture analyses (CTTA) in patients with colorectal liver metastases (CRLM) are able to predict and identify local tumour progression (LTP) after thermal ablation.
**Material and methods:** We retrospectively included patients with up to 5 CRLM (size ≤3cm), who underwent RFA or MWA and had follow-up ≥6 months. An experienced reader manually delineated all the CRLM and ablation zones (AZ) on portal venous CT pre- and 4-8 weeks post-ablation. Texture parameters were extracted from the delineated volumes of interest (VOIs) with Pyradiomics, using different Laplacian of Gaussian (LoG) filters. Subsequently, texture parameters were compared between lesions with and without LTP, and between patients with new metastases and those who remained disease free.
**Results:** 38/168 lesions (in 104 patients) developed LTP and 54 patients developed new metastases. Median follow-up time was 20 (range 6-134). Uniformity of CRLM pre-ablation was significantly higher in patients who developed new metastases than in those who remained disease free (mean 0.043 vs 0.039, p=0.029, unfiltered and mean 0.046 vs 0.042, p=0.066, LoG filter s0.5). Furthermore, mean grey-level intensity of the AZ (post-ablation) was significantly higher in lesions that developed LTP compared to those that did not (mean 1.18 vs 0.92, p=0.037, LoG filter σ0.5).
**Conclusion:** A higher uniformity of CRLM before ablation shows the potential for identifying patients at risk of developing new metastases. Additionally, AZ of lesions that develop LTP post-ablation shows a higher mean grey-level intensity. CTTA of both the CRLM (pre-ablation) and of the AZ (4-8 weeks after ablation) may, therefore, be predictive of the development of LTP and new metastases.

### SS 5.8

### CT texture analysis to predict response to HER2-targeted therapy of hepatic metastases from colorectal cancer

S. Mazzetti[1], V. Giannini[1], G. Cappello[1], A. Vanzulli[2], S. Marsoni[2], D. Regge[1]; [1]Candiolo/IT, [2]Milan/IT

**Purpose:** To use CT texture analysis (CTTA) to identify specific imaging biomarkers of hepatic metastases, able to predict patient's response to therapy and overall survival.
**Material and methods:** We exploited the imaging dataset of HERACLES trial (NCT03225937): 23 patients with amplified HER2 mCRC were included in the study. All had received anti-HER2 treatment, and underwent CT examination every 8 weeks, until disease progression. CT scans were semi-automatically segmented to extract all liver metastases. CTTA was performed on each segmented area, computing for each lesion 34 quantitative parameters. Monoparametric and multi-parametric analyses were assessed to identify features correlated to therapy response. We also performed a correlative survival (OS) analysis, considering subjects with good survival those with OS>9 months.
**Results:** In 23 patients we found 124 metastases, 55 classified as responding and 69 as nonresponding. Nine parameters reached statistical significance in mono-parametric analysis (best AUC=0.67, p=0.001), while in multivariate regression, ten parameters were used in the model, achieving AUC equal to 0.82, sensitivity of 82% and specificity of 72%. For OS analysis, 12 patients were "good" and 11 "poor" survivors. In mono-parametric analysis "cluster prominence" and "sum entropy" predicted OS with AUC equal to 0.78 and 0.83, respectively. The regression model with two variables ("cluster prominence" and "dissimilarity") reached sensitivity of 83% and specificity of 82%.
**Conclusion:** Our study demonstrated CTTA as a potential biomarker to predict response of hepatic metastases to target therapy, possibly saving patients predicted as non-responder from toxicity. Moreover, CTTA could give indications on patients OS.

### SS 5.9

### Portosystemic shunt surgery in the era of transjugular portosystemic shunts: imaging-based procedure planning

U. Fehrenbach, M. De Sousa Mendes, S. Gül, I. Steffen, J. Stern, D. Geisel, G. Puhl, J. Pratschke, T. Denecke; Berlin/DE

**Purpose:** With the upcoming of transjugular portosystemic shunts (TIPS), the once preferred portosystemic shunt surgery is limited to those patients in which TIPS is impossible. This leaves few but more complicated cases with great demands on diagnostic imaging for accurate preoperative planning. The aim of this study was to evaluate whether CT and/or MRI of the visceral veins are reliable for planning of portosystemic shunt surgery in patients unsuitable to be treated by TIPS.
**Material and methods:** A total of 46 consecutive patients treated with portosystemic shunt surgery (2008 to 2013) were retrospectively identified. The patients underwent contrast-enhanced CT (n=34) and/or MRI (n=16) prior to surgery. The analysis of these images (venous anatomy, patency, diameter, intervenous distances and structures) was performed by two independent radiologists (observer 1(O1): 10+ years' experience; observer 2(O2): 4-year experience). The common shunt techniques were selected (i.e. portocaval; mesocaval; distal, proximal or side-to-side splenorenal) and ranked according to their appropriateness and complexity. The ranking was compared with the actual successfully performed shunt procedure.
**Results:** The technique recommended by our observers corresponded to the surgeons' intraoperative choice in O1: 94.1%/75.0% (CT/MRI) and O2: 73.5%/62.5% of the cases, respectively. The predicted technical complexity was confirmed by the required intraoperative tissue resection and necessity of interposition grafts in 85%. Prediction based on CT was superior to MRI in both observers; however, without statistical significance (p=0.074 and 0.471).
**Conclusion:** CT and MRI optimized for abdominal veins are accurate methods for the planning of portosystemic shunt surgery. Selection of an appropriate surgical approach requires experienced readers.

### SS 5.10

### Clinical impact of a new cone beam CT angiography respiratory motion artifact reduction algorithm during hepatic intra-arterial treatment

M. Dioguardi Burgio[1], T. Benseghir[2], V. Roche[1], C. Garcia Alba[1], J.-B. Debry[1], A. Sibert[1], V. Vilgrain[1], M. Ronot[1]; [1]Clichy/FR, [2]Buc/FR

**Purpose:** To assess the impact of a dedicated "respiratory motion correction software" on contrast-enhanced cone beam CT angiography (CBCTa) during intra-arterial liver-directed therapy.
**Material and methods:** From 2015 to 2017, two groups of patients undergoing intra-arterial liver-directed therapy with (breathing, n=30) or without (still, n=30) significant respiratory motion artifacts were retrospectively included. All CBCTa were processed with and without a dedicated motion correction software. For both reconstructions (with and without motion correction), four readers were asked to independently assess: 1) the overall image quality on a 5-point scale, and 2) the presence of per-procedural relevant information on tumor and vasculature (overall vessel geometry; visibility of extrahepatic vessels; target tumor conspicuity; visibility of tumor feeders) on a 3-stage scale (good/intermediate/poor).
**Results:** In the breathing group, motion correction increased the average image quality from 2.0±0.9 to 2.9±1.0 (p<0.01). The visibility of vessel geometry, extrahepatic vessels, and tumor feeders were significantly improved for all 4 readers, and that of tumor conspicuity was improved for 3 readers. In the still group, the average image quality was not significantly different between reconstructions (with and without motion correction) for all readers (4.0±0.6 vs 4.2±0.6; p=0.12). Visibility of vessel geometry, extrahepatic vessels, tumor feeders and tumor conspicuity were not altered for all 4 readers using the correction software.
**Conclusion:** Using dedicated motion correction software increases both the image quality and the visualization of the per-procedural relevant information on tumor and vasculature needed during intra-arterial liver-directed procedures of motion-corrupted CBCTa, while maintaining that of still CBCTa unaltered.

11:00 - 12:30 Room Florence

*Scientific Session SS 6*
### Diagnosis of HCC

### SS 6.1
### Hepatobiliary phase hypointensity in LR-3 and LR-4 observations as a predictor of progression to HCC
F. Vernuccio[1], R. Cannella[1], M. Meyer[2], K.R. Choudhoury[2], M.R. Bashir[2], A. Furlan[3], D. Marin[2]; [1]Palermo/IT, [2]Durham, NC/US, [3]Pittsburgh, PA/US

**Purpose:** To determine imaging predictors of progression to HCC in observations ≥10 mm in size and arterial phase hyperenhancement (APHE) in cirrhotic patients surveilled with gadoxetate-MRI.
**Material and methods:** This retrospective, dual-institution study included patients at risk for HCC who underwent gadoxetic acid liver MR between January 2009 and December 2014, and having one or more liver lesions ≥10 mm in size, with APHE, and not already categorized as LR-5 using LI-RADS. For each observation, the readers documented the presence of (i) "washout", (ii) "capsule", (iii) threshold growth, (iv) hepatobiliary hypointensity and the outcome of the lesion. Progression rate to HCC was calculated. The association of imaging features with progression to HCC was analyzed using univariate and multivariate analyses.
**Results:** Our final study population comprised 128 patients (mean age ± SD, 57 years ± 11) and a total of 186 APHE lesions ≥10 mm in size (mean diameter± SD, 14 mm ± 4 mm), including 160 (67%) LR-3 and 26 (14%) LR-4. Progression rate to HCC was significantly higher for LR-4 compared to LR-3 (81% [21 of 26] vs. 56% [89 of 160], respectively; p = 0.015563). Hepatobiliary phase hypointensity was the only independent predictor at multivariate analysis (OR: 19.3; log-odds: 2.96; P< $10^{-13}$).
**Conclusion:** Progression rate to HCC of LR-3 and LR-4 APHE lesions ≥10 mm is not negligible. Hepatobiliary phase hypointensity is an independent predictor of progression to HCC.

### SS 6.2
### Evaluation of Liver Imaging Reporting and Data System v2018 by MR in ≤ 2 cm US-detected nodules in cirrhotic patients
A. Darnell, J. Rimola, E. Belmonte, E. Ripoll, C. Caparroz, Á. Díaz-González, M.E. Reig, C. Ayuso, J. Bruix, A. Forner; Barcelona/ES

**Purpose:** To evaluate the diagnostic accuracy of Liver Imaging Reporting and Data System (LI-RADS) v2018 when using MRI for hepatic nodules ≤20mm detected during US surveillance in cirrhotic patients.
**Material and methods:** Between November 2003 and February 2017, we included 262 cirrhotic patients with a newly US detected solitary ≤ 20 mm hepatic nodule who were prospectively examined by MRI and fine-needle biopsy (reference standard) and followed up with MRI every 6 months if the initial definitive diagnosis was not achieved. A LI-RADS (LR) category according to v2018 was retrospectively assigned. The diagnostic accuracy for each LR category was described and the main MRI findings associated with HCC diagnosis were analyzed.
**Results:** Final diagnoses were 197 HCC (75.2%), 5 intrahepatic cholangiocarcinoma (1.9%), 2 metastasis (0.8%) and 58 benign lesions (22.1%). 0/15 LR-1 (0%), 6/26 (23.1%) LR-2, 51/74 (68.9%) LR-3, 11/12 (91.7%) LR-4, 126/127 (99.2%) LR-5, and 3/8 (37.5%) OM were HCC. LR-5 category displayed a sensitivity of 64.5% (CI95%: 57.4-71.1), and considering also LR-4 as diagnostic for HCC, the sensitivity slightly increased to 69.5% (CI95%: 62.6-75.9) with minor impact on specificity (96.2%; CI95%: 89.3-99.6). Regarding LR-3 observations, 51 out 74 were HCC, 2 were non-HCC malignancies, and twenty-one out 22 LR-3 nodules >15 mm (95.5%) were finally categorized as HCC.
**Conclusion:** In cirrhotic patients with nodules ≤20 mm detected on US, the distinction between LR-4 and LR-5 according to v2018 has a minor impact. In addition, a relevant proportion of LR-3 observations corresponded to an HCC, justifying an active diagnostic workup.

### SS 6.3
### Comparison of the Performance of European Association for the Study of the Liver 2018 and Liver Imaging Reporting and Data System 2018 for the noninvasive diagnosis of HCC using MRI
S. Lee, M.-J. Kim; Seoul/KR

**Purpose:** We compared the diagnostic performance of European Association for the Study of the Liver (EASL) 2018 and Liver Imaging Reporting and Data System (LI-RADS) 2018 criteria on MRI for the noninvasive diagnosis of HCC at high-risk patients and evaluated the diagnostic value between MRI with extracellular contrast agents (ECA-MRI) and MRI with hepatobiliary agents (HBA-MRI).
**Material and methods:** This retrospective study included 482 surgically confirmed lesions (428 HCCs, 8 benign lesions, and 46 other malignancies) in 413 patients at high risk of HCC who underwent preoperative multiphasic contrast-enhanced MRI from January 2015 to December 2017. Two readers assessed all observations according to EASL 2018 and LI-RADS 2018 criteria and the per-observation diagnostic performances were compared.
**Results:** EASL 2018 had a higher sensitivity (77.1% vs. 70.6%, P = 0.002) but a lower specificity (53.7% vs. 77.8%, P < 0.001) than LR-5 according to LI-RADS 2018. In combining LR-5 and LR-4, LI-RADS 2018 showed better sensitivity (83.4% vs. 77.1%, P = 0.019) and specificity (74.1% vs. 53.7%, P = 0.004) than EASL 2018. The sensitivity and specificity in EASL 2018 did not show statistically significant differences between ECA-MRI and HBA-MRI. HBA-MRI showed a significantly higher specificity than ECA-MRI in LR-5 according to LI-RADS 2018 (88.6% vs. 57.9%, P = 0.016).
**Conclusion:** LR-5 according to LI-RADS 2018 had a lower sensitivity but higher specificity than EASL 2018. The combinations of LR-5 and LR-4 according to LI-RADS 2018 achieved better diagnostic performance than EASL 2018.

### SS 6.4
### Dynamic contrast-enhanced MRI features for identifying macrotrabecular-massive HCC
A. Galletto Pregliasco, S. Mule, G. Amaddeo, L. Baranes, F. Pigneur, R. Kharrat, M. Djabbari, H. Regnault, C. Hezode, J. Caldéraro, A. Luciani; Créteil/FR

**Purpose:** To determine whether preoperative MRI may help identify the recently identified "macrotrabecular-massive" histological subtype of HCC, associated with poor survival, in patients with HCC treated with surgical resection.
**Material and methods:** This institutional-approved retrospective study included 152 patients with HCC (26 of them with "macrotrabecular-massive" subtype) who underwent curative resection after dynamic contrast-enhanced MR imaging in a single institution. Two radiologists evaluated preoperative HCC MR imaging features. Lasso-penalized and multivariate logistic regression analyses were performed to identify features significantly associated with the "macrotrabecular-massive" HCC subtype.
**Results:** The Lasso-penalized logistic regression analysis identified massive central necrosis, arterial rim enhancement, and macrovascular invasion as independent features associated with "macrotrabecular-massive" HCCs. At multivariate logistic regression analysis with backward selection, massive central necrosis (odds ratio (95% CI) = 21.47 (7.33, 62.93), p<0.0001) was confirmed as an independent predictor of the "macrotrabecular-massive" HCC histological subtype. With that imaging feature, 65.4% (17 of 26; 95% CI: 44.3%, 82.8%) of macrotrabecular-massive HCCs were identified with a specificity of 92.9% (117 of 126; 95%CI: 86.9%, 96.7%).
**Conclusion:** In dynamic contrast-enhanced MRI, massive central necrosis may help identify the macrotrabecular-massive HCC subtype.

### SS 6.5

### Modified v2018 Liver Imaging Reporting and Data Systems: a prospective comparative study on gadoxetic acid-enhanced MRI

H. Jiang, B. Song; Chengdu/CN

**Purpose:** We aimed to develop several modified Liver Imaging Reporting and Data Systems (LI-RADS) and to compare their accuracies with the original LI-RADS version 2018 (v2018) for diagnosing HCC.
**Material and methods:** We obtained ethical approval by the institutional review board and informed consent for this prospective study. Between July 2015 and February 2018, high-risk patients with suspected liver lesions were consecutively enrolled and underwent gadoxetic acid-enhanced MRI. We constructed the following modified LI-RADS models: model 1 with "restricted diffusion" upgraded, model 2 with "hepatobiliary phase hypointensity" upgraded and model 3 with both features upgraded as major features. All images were reviewed by two independent radiologists blindly. The diagnostic accuracies were determined on their sensitivity and specificity and were compared with the McNemar test.
**Results:** 122 surgically confirmed nodules (mean size: 5.03±2.88cm, 66 HCCs) in 109 patients (26 female) were included. The sensitivity and specificity in consensus of the v2018 LI-RADS criteria were 77% and 82% for the LR5/LR5-TIV combination, and 95% and 77% for the LR4/LR5/LR5-TIV combination, respectively. These measures were 83% and 79% for model 1, 82% and 80% for model 2, and 83% and 77% for model 3, respectively. The sensitivity of the LR4/LR5/LR5-TIV combination was significantly higher than the remaining models (p=0002 to 0.021), while the specificities among all models did not differ significantly (p=0.482 to 1.000).
**Conclusion:** The modified LI-RADS models were no more accurate than the original v2018 LI-RADS, and the LR4/LR5/LR5-TIV combination showed potential in improving the diagnostic sensitivity with no substantial loss of specificity.

### SS 6.6

### Inter-reader, intra-reader agreement, and correlation with pathology of CT/MRI Liver Imaging Reporting And Data System v2018

A. Borgheresi, A. Agostini, D. Nicolini, G. Conte, E. Dalla Bona, M. Romano, M. Vivarelli, A. Giovagnoni; Ancona/IT

**Purpose:** To evaluate intra- (IRA) and inter-reader agreement (IRE) accuracy and correlation with pathology of CT/MRI Liver Imaging Reporting and Data System (LI-RADS) v2018 in cirrhotic patients resected for HCC.
**Material and methods:** Ninety-five patients (mean age 64; range 56-73) with chronic liver disease and focal observation underwent to surgical resection between June 2015 and July 2018. All the included patients had at least one triphasic contrast-enhanced CT or MRI within a month prior to surgery. Three readers with different experience in abdominal radiology (A=30, B=10; C=5 years) assigned in blind the LI-RADSv2018 categories and features in separate sessions. LI-RADS categories and features were correlated with microvascular invasion (MIV) and Edmonson grade on surgical specimen by Chi-square test. IRE and IRA were calculated with intraclass correlation coefficient (ICC).
**Results:** 115 lesions (median diameter 22 mm) were evaluated. LI-RADS score derived on major features only had moderate IRA and IRE in CT (ICC: 0.597 and 0.608) and in MRI (ICC: 0.572 and 0.584). LI-RADS categories built major and ancillary features had moderate IRA and IRE in CT (ICC: 0.712 and 0.697) and in MRI (ICC: 0.688 and 0.691). Tumor size, Corona enhancement and rim APHE (arterial phase enhancement) were significantly related to the presence of MIV (p<0.05). Targetoid mass and infiltrative appearance significantly correlated with G3 (p<0.05).
**Conclusion:** Application of LI-RADSv2018 score allowed for a moderate IRA and IRE in readers with different experience. Significant correlations between some LI-RADSv2018 features and pathological features were found.

### SS 6.7

### Additional value of contrast-enhanced US on arterial phase non-hyperenhancement observations (≥ 2cm) of CT/MRI for high-risk patients: focusing on the CT/MRI Liver Imaging Reporting and Data System categories LR-3 and LR-4

J.H. Kim, H.-J. Kang, I. Joo, J.K. Han; Seoul/KR

**Purpose:** To determine the incremental value of CEUS on larger than 2cm size arterial phase non-hyperenhancement (APNHE) observation (Liver Imaging Reporting And Data System (LI-RADS) LR-3 and LR-4) on CT/MRI in high-risk patients.
**Material and methods:** Forty-three APNHE observations (≥ 2cm) on CT/MRI in 43 patients were prospectively enrolled in this IRB approval study and underwent CEUS. All lesions were assessed categorized by LI-RADS for CT/MRI and CEUS. The hemodynamic findings were compared with CEUS and CT/MRI. Except nine observation underwent immediate RFA, thirteen observations (38.2%, 13/34) were diagnosed as malignancy (n=12, HCCs; n=1, cholangiocarcinoma). Mean follow-up period was 11.8±2.1 months.
**Results:** Mean size of target lesion were 2.3±0.3 cm. Among 43 APNHE observations, 12 observations (27.9%) further presented arterial phase hyperenhancement (APHE) on CEUS with early (n=1, CEUS LR-M), late (n=10, CEUS LR-5) or no washout (n=1, CEUS LR-4). Sixteen (44.4%) and twenty (55.6%) presented concordant enhancement pattern between CEUS and CT in AP and PVP, respectively. Similarly, thirteen (59.1%), fourteen (63.6%) and ten (45.5%) observations showed concordant enhancement pattern between CEUS and MRI in AP, PVP and HBP, respectively. Of the 34 diagnosed APNHE observations, 6 (17.6%) showed APHE on CEUS with early (n=1, CEUS LR-M) or late (n=5, CEUS LR-5) washout. Thus, sensitivity, specificity and accuracy were further increased (33.3%, 95.4% and 73.5%, respectively) by CEUS application.
**Conclusion:** Applying CEUS on APNHE observations in CT/MRI, it is useful not only for diagnosis of definitely HCC (CEUS LR-5) but also other malignancy (CEUS LR-M) with specificity increment. Discordance of dynamic feature may reflect the different properties of CT/MRI and CEUS.

### SS 6.8

### Texture analysis on preoperative imaging identifies patients with high-risk HCC

R. Cannella[1], G.C. Wilson[2], G. Fiorentini[2], C. Shen[2], A.A. Borhani[2], A. Tsung[2], A. Furlan[2]; [1]Palermo/IT, [2]Pittsburgh, PA/US

**Purpose:** The aim of this study is to determine if radiomics can predict biologic aggressiveness in HCC and identify tumors with microvascular invasion (MVI).
**Material and methods:** Single-center, retrospective review of HCC patients undergoing resection/ablation with curative intent from 2009 to 2017. Preoperative MRIs were analyzed with texture analysis software placing a single region of interest on the slice with largest tumor cross section. Texture analysis parameters were extracted on pre-contrast T1-, T2-weighted, hepatic arterial phase (HAP) and portal venous phase (PVP) images. Multivariate logistic regression analysis evaluated factors associated with MVI. Kaplan-Meier methods were used for survival analysis.
**Results:** MVI was present in 52.2% (n=133) of HCCs. Disease-free survival was worse in MVI-positive HCC (median survival: 14.8mos), compared to MVI-negative HCC (median survival: 28.7mos, p=0.012). Texture analysis parameters associated with MVI included T1 mean (OR=0.98 95%CI 0.96–1.0, p=0.068), T1 skewness (OR=3.4, 95%CI 0.89–13.3, p=0.075), T1 kurtosis (OR=1.75, 95%CI 0.95–3.2, p=0.074), T2 entropy (OR=3.8, 95%CI 1.1–13.0, p=0.034), HAP mean (OR=0.99, 95%CI 0.98–1.00, p=0.128) and PVP entropy (OR=4.3, 95%CI 1.2–15.1, p=0.025). Tumor characteristics associated with MVI included tumor size (OR=1.33, 95%CI 0.98–1.8, p=0.067), tumor grade (OR=3.02, 95%CI 0.83–11.03, p = 0.093), and minimal liver fibrosis (OR=0.19, 95%CI 0.03–1.1, p=0.064). On multivariate analysis, only T1 mean (OR=0.97, 95%CI 0.95–0.99, p=0.043) and PVP entropy (OR=4.7, 95%CI 1.37–16.3, p=0.014) were associated with tumor MVI. Area under ROC curve was 0.83 for this final model.
**Conclusion:** Tumor entropy and mean are both associated with MVI on final tumor pathology.

### SS 6.9

### Assessment of histologic grade and microvascular invasion in HCC with gadobenate dimeglumine-enhanced MRI

D.L. Rong, S.C. Kuang, S.D. Xie, Y. Zhang, B.J. He, Y. Deng, H. Yang, S.M. Chen, J. Wang; Guangzhou/CN

**Purpose:** To evaluate the role of gadobenate dimeglumine (Gd-BOPTA)-enhanced MRI in the assessment of histologic grade and microvascular invasion (MVI) in HCC.

**Material and methods:** This retrospective study was approved by the institutional review board and written informed consent was waived. 51 patients who underwent enhanced MR using Gd-BOPTA were included. The enhancement ratio (ER) of the tumor, contrast-to-noise ratio of tumor to liver ($CNR_{T/L}$) and tumor to muscle ($CNR_{T/M}$) in pre-enhanced T1WI, arterial phase (AP), portal vein phase (PVP), delayed phase (DP) (2min, 3min, 5min, 10min) and 2-hour hepatobiliary phase (HBP) were calculated and compared with histologic grade and MVI using Student's t test, respectively. Diagnostic performance was evaluated using area under ROC curve (AUC) analysis.

**Results:** WD/MD-HCCs showed higher ERs compared to PD-HCCs in $DP_{5min}$ (P<0.05), $DP_{10min}$ (P<0.01), HBP (P<0.01), respectively. The best performance to predict histologic grade was found in HBP, with an AUC of 0.824, followed by $DP_{10min}$ (0.763), $DP_{5min}$ (0.729). The cut-off values in predicting PD HCC were ER<16.86% (sensitivity, 87.8%; specificity, 80%) in HBP, <37.62% (sen, 85.4%; spe, 70%) in $DP_{10min}$ and <55.77% (sen, 63.4%; spe, 80%) in $DP_{5min}$. Only ER in $DP_{10min}$ could predict MVI, with an AUC of 0.674. The cut-off values in predicting MVI in $DP_{10min}$ were ER <21.57%(sen, 86.4%; spe, 45.5%).

**Conclusion:** The ERs in $DP_{5min}$, $DP_{10min}$ and HBP are potential quantitative markers for predicting histologic grade. Only ER in $DP_{10min}$ is effective for predicting MVI of HCC in Gd-BOPTA-enhanced MRI.

### SS 6.10

### Incomplete tumor capsule on preoperative imaging reveals microvascular invasion in HCC: a systematic review and meta-analysis

F. Zhu, F. Yang, J. Li, W. Chen, W. Yang; Chengdu/CN

**Purpose:** Microvascular invasion (MVI), which is difficult to diagnose before surgery, is a major factor affecting postoperative recurrence in patients with HCC. The relationship between the radiological tumor capsule and MVI is controversial. This study aimed to evaluate the association between the tumor capsule and MVI.

**Material and methods:** We searched Medline (by PubMed) and Embase (by OvidSP). Two review authors independently screened titles and abstracts, selected studies for inclusion, assessed the methodological quality of included studies, and collected data. Summary results were presented as the diagnostic odds ratio (DOR), sensitivity, specificity, and 95% confidence interval.

**Results:** Fifteen studies were included; fourteen studies with no significant heterogeneity indicated no statistically significant relationship between a present or absent tumor capsule and MVI [DOR = 0.90 (0.64, 1.26)]. Six studies with no significant heterogeneity showed an incomplete capsule could be used to predict MVI of HCC preoperatively [DOR = 1.85 (1.13, 3.04)]. The overall sensitivity and specificity estimates were 0.50 (0.37, 0.64) and 0.64 (0.53, 0.74), respectively. Eight studies with highly significant heterogeneity revealed that complete capsule could be a protective factor for MVI [DOR = 1.97 (1.01, 3.86)].

**Conclusion:** For microvascular invasion of HCC, the tumor capsule may be both a protective factor and a risk factor. An incomplete tumor capsule is a risk factor, while a complete tumor capsule may be a protective factor. Further research on the integrity of the capsule and MVI is needed to help predict MVI.

**11:00 - 12:30** **Room Pisa**

*Scientific Session SS 7*

### Machine learning and radiomics in abdominal imaging

### SS 7.1

### Machine learning-based analysis of CT radiomics model for the prediction of colorectal metachronous liver metastases

M. Taghavi[1], S. Trebeschi[1], D. Meek[1], D. Lambregts[1], R.C.J. Beckers[2], R. Dijkhoff[2], C. Verhoef[3], J.B. Houwers[2], U. Van Der Heide[1], R.G.H. Beets-Tan[1], M. Maas[1]; [1]Amsterdam/NL, [2]Maastricht/NL, [3]Rotterdam/NL

**Purpose:** To develop and validate a CT-based radiomics model for the prediction of metachronous colorectal liver metastases (CRLM) at primary staging with a machine learning algorithm.

**Material and methods:** 91 colorectal cancer (CRC) patients from three centers were included. Two groups were assessed: patients who had no evidence of CRLM at primary staging or during follow-up of ≥ 24 months (n=67) and patients who had no evidence of liver metastases at primary staging but developed metachronous CRLM < 24 months (n=24). After liver parenchyma segmentation (excluding vessels and benign liver lesions), 7,344 radiomics features were extracted from the primary staging CT per patient with PyRadiomics package. Patients were divided into a training set (n=54) and an independent validation set (n=37). Two predictive models were built based on only radiomics features and with the combination of clinical information, these models were compared regarding predictive value. Bayesian-optimized random forest with wrapper feature selection (5-fold cross-validation) was trained on the training set and optimized with sequential model-based optimization.

**Results:** The area under the curve (AUC) of the machine learning model based on CT radiomics feature performance in the training cohort was 0.88(95%CI: 0.85-0.92), and in the validation cohort it was 0.87(95%CI: 0.82-0.91) with sensitivity 0.88(95%CI 0.80-0.95) and specificity 0.72(95%CI 0.63-0.82). Adding clinical features to the radiomics model did not improve diagnostic performance.

**Conclusion:** A machine learning-based radiomics analysis of routine clinical CT imaging at diagnosis could provide valuable biomarkers to predict patients at risk for developing colorectal liver metastases.

### SS 7.2

### Zero-click liver proton density fat fraction and R2* quantification by deeply supervised convolutional neural networks applied to multi-echo chemical sift-encoded MR

A. Jimenez-Pastor, A. Alberich-Bayarri, R. Lopez-Gonzalez, M. Garcia-Junco, L. Marti-Bonmati, D. Marti-Aguado; Valencia/ES

**Purpose:** Diffuse liver diseases such as non-alcoholic fatty liver disease or non-alcoholic steatohepatitis may progress to liver fibrosis if not detected at early stages. Multi-echo chemical shift-encoded (MECSE) MR sequences allow the simultaneous quantification of proton density fat fraction (PDFF) and R2*. However, their volumetric calculation requires liver segmentation, which is a time-consuming task that can take hours. The main purpose of our work was to develop an automated analysis pipeline including 3D segmentation of liver parenchyma in MECSE-MRI using deeply supervised convolutional neural networks (CNN).
**Material and methods:** A total of 134 MECSE-MR studies were retrospectively collected and manually segmented using ITK-SNAP. 80% of the dataset was used for CNN model training and the remaining 20% for test. The developed architecture was an encoder-decoder CNN, where deep-supervision layers were used to obtain a faster convergence on the training process and to increase the accuracy in delineation of liver boundaries. The CNN architecture generalization was evaluated by a 5-fold cross validation. To analyze the model robustness, the test over 22 previously unseen studies was performed by calculating the DICE coefficient (DC).
**Results:** In the 5-fold cross-validation, a mean DC of 95.16±0.18% was obtained, proving the CNN architecture generalization capability. In the test images, a mean DC of 93.15±2.79% was obtained, demonstrating the final trained-model robustness.
**Conclusion:** Radiologists' workflow in the assessment of diffuse liver diseases can be improved by the automatic segmentation of the liver on MECSE-MR images. This can be addressed by deeply supervised convolutional neural networks, automating the quantification of imaging biomarkers, such as the PDFF or the R2*.

### SS 7.3

### CT texture analysis of liver metastases in pancreatic neuroendocrine tumors versus non-pancreatic neuroendocrine tumors: correlation with histopathological findings

I. Martini, M. Polici, M. Zerunian, F. Landolfi, F. Panzuto, M. Rinzivillo, A. Laghi, E. Iannicelli; Rome/IT

**Purpose:** To compare CT -texture analysis and CT features of liver metastases in pancreatic neuroendocrine tumours (PNETs) and in non-pancreatic neuroendocrine tumours (NPNETs) according to tumour grading.
**Material and methods:** Contrast-enhanced CT images of liver metastases in 23 patients with PNETs and in 25 patients with NPNETs were analysed with 3D CT texture analysis (parameters evaluated: mean attenuation, standard deviation, skewness, kurtosis, entropy, mean of positive pixels and Tx_sigma) in arterial and portal phase; delta enhancement of the lesions was also calculated. The CT exams were performed before the beginning of any medical treatment. All patients presented a well-differentiated tumour according to WHO classification (G1 and G2). Data were analysed with Mann-Whitney and Chi-squared tests.
**Results:** Among CT texture analysis, in a comprehensive comparison between PNETs and NPNETs, the parameter "Skewness" was significantly higher in NPNETs (p value<0.05). These data were confirmed in subgroup comparisons evaluated in portal phase (NPNETs G1 vs PNETs G1, NPNETs G2 vs PNETs G2, and NPNETs G1+G2 vs PNETs G1+ G2). The parameter "Mean" was significantly higher in PNETs in comparison to NPNETs (p value=0,0066). Among CT features the "delta enhancement" was significantly higher (p value<0.05) in PNETs in two comparisons: PNETs G1 Vs NPNETs G1 and PNET Vs NPNETs.
**Conclusion:** These results demonstrate significant differences between CT texture parameters of liver metastases in PNETs and NPNETs, and highlight the diversity of the two groups of NETs. These findings, in future, could be used as an "imaging biomarker" to predict therapy response.

### SS 7.4

### Histogram and texture features in baseline diffusion-weighted MRI predict tumor subtype, therapy response and patient survival in pancreatic ductal adenocarcinoma

G. Kaissis[1], S. Ziegelmayer[1], F. Lohöfer[1], I. Heid[1], J. Siveke[2], W. Weichert[1], E.J. Rummeny[1], R. Braren[1]; [1]Munich/DE, [2]Essen/DE

**Purpose:** To evaluate the apparent diffusion coefficient (ADC) map histogram-derived quantitative imaging parameter ADC entropy as a biomarker in pancreatic ductal adenocarcinoma (PDAC).
**Material and methods:** We retrospectively analyzed 39 cases of resected and 21 cases of metastatic PDAC who underwent clinical 1.5T-MRI assessment. Whole-tumor volumes were segmented and quantitative imaging parameters were derived using LifeX. Disease-free (DFS) and overall survival (OS) were compared between patients with ADC entropy above vs. below the median of 6.0. Histopathological heterogeneity and immunohistochemical subtype of tumor samples were determined. The effect of gemcitabine treatment on survival was compared between ADC entropy groups.
**Results:** High ADC entropy led to worse median DFS (8.7 vs. 21.6 months) and OS (12.7 vs. 27.5 months) (N=39, p<0.0001) in the surgical patient cohort. Patients in the high-ADC entropy group had a 90% increased risk of high morphological tumor heterogeneity (p=0.09) and all patients with a quasi-mesenchymal molecular tumor subtype (n=9) belonged to this group. Patients with high ADC entropy who underwent gemcitabine treatment (N=25) had significantly improved DFS (5.5 to 9.5 months, p=0.04) and OS (5.5 to 14.6, p=0.01) while gemcitabine had no significant effect on DFS or OS in the low-ADC entropy group (N=14). All metastatic PDAC cases belonged to the high-ADC entropy group. Gemcitabine-based treatment provided a slight DFS benefit to these patients compared to FOLFIRINOX (11.6 vs. 7.6 months, p=0.15).
**Conclusion:** High ADC entropy predicts worse survival, improved gemcitabine-based chemotherapy response and is associated with increased tumor heterogeneity and the quasi-mesenchymal molecular subtype in PDAC.

### SS 7.5

### Synergistic analysis of micro-positron emission tomography/MRI data using deep learning for automatic detection of HCC

H.C. Chang[1], T.Y. Huang[2], E.S. Hui[1], W.H.K. Chiu[1]; [1]Hong Kong/HK, [2]Taipei/TW

**Purpose:** The purpose of this study is to investigate the benefit of synergistic analysis of positron emission tomography (PET) and MRI data using deep learning for automatic detection and segmentation of HCC.
**Material and methods:** The micro-PET/MRI data were retrospectively collected from an animal study with orthotropic HCC tumor model conducted in our institution. Totally thirty-eight sets of coronal F18-FDG and corresponding T2WI images were selected for preliminary test. The labeled images were generated by drawing the ROI of tumors on T2WI images. Afterward, 28 and 10 sets of images were, respectively, used for network training and validation. The SegNet network architecture was selected for this implementation with a multi-channel data input and was pre-trained with BRATS data (brain data with 600,000 steps). For micro-PET/MRI data, the training steps were 10,000 with 6 images for each step. Three types of inputs (both F18-FDG and T2WI, F18-FDG only, and T2WI only) were tested for the efficacy of tumor detection using SegNet.
**Results:** The combination of PET and MRI information (F18-FDG and T2WI) provided a best mean dice coefficient (0.69), compared to either using PET data (0.47) or MRI data (0.58) for tumor detection and segmentation.
**Conclusion:** This preliminary study shows that the synergistic analysis of PET and MRI data using deep learning is feasible for automatic detection and segmentation of HCC, and provides better performance than using either individual PET or MRI data. Additionally, the transfer learning can ensure a proper training of the network, even though with limited amount of training data.

### SS 7.6

### Assessing hepatic steatosis on US imaging using deep learning

J.S.N. Tang, J.C.Y. Seah, D. Mitchell, T. Lovell, M.W. McCusker, W. Wang, R.N. Gibson, F. Gaillard; Melbourne, VIC/AU

**Purpose:** To develop a deep learning algorithm which can assess hepatic steatosis.
**Material and methods:** All abdominal ultrasounds from a tertiary centre from January 2013 to November 2017 were reviewed. Studies with known focal hepatobiliary pathology were excluded. Of the 1509 candidate studies, 505 were considered suitable for inclusion. From each study, four ultrasound images of the right lobe of the liver were chosen, two transverse and two longitudinal. Two radiologists and one radiology fellow reviewed each panel of four images and assigned a number of 1 (normal) - 7(severe steatosis). Subsequently, a consensus was agreed upon by all three radiologists, which was used as the gold standard. 2020 images were divided by patients into training, validation and testing sets in a ratio of 8:1:1. Images were scaled to 256 x 256. A modified Densenet model pre-trained upon ImageNet was trained and validated. Quadratic weighted Cohen's Kappa was chosen as the evaluation metric.
**Results:** The most common category was two (normal-mild steatosis), with 159 (32%) studies. 17 (3%) were in the least common category which was category seven (severe steatosis). The radiologists achieved a quadratic weighted Cohen's Kappa of 0.82–0.95 and the Densenet model achieved a quadratic weighted Cohen's Kappa of 0.87. The most commonly misclassified category amongst the radiologists was three (mild) whilst the most commonly misclassified categories for the Densenet model were tied at three and four (mild, mild-moderate).
**Conclusion:** The deep learning algorithm performs similar to subspecialty trained radiologists in the assessment of hepatic steatosis on ultrasound.

### SS 7.7

### Radiomic analysis in HCC to predict clinical outcome after liver transplantation: a preliminary study

R. Faletti, M. Gatti, A. Di Chio, F. Guarasci, F. Tandoi, R. Romagnoli, P. Fonio; Turin/IT

**Purpose:** To evaluate the role of radiomics in preoperative triple-phase CT to predict recurrence in patients undergone orthotopic liver transplantation (OLT) for HCC.
**Material and methods:** Out of 202 patients who underwent OLT for HCC in our centre between 2011 and 2015, we retrospectively selected 8(4%) patients (8 males, mean age 63y, 20 lesions) who had HCC recurrence after OLT and underwent three-phase CT within 3 months prior to treatment. Eleven age- and sex-matched patients (9 males, mean age 63y, 22 lesions) with at least 2-year disease-free follow-up served as control. IBEX® software was used to analyze 152 texture parameters for each CT phase (total of 456 features). LASSO logistic regression was used to select features and develop three radiomic signatures with different values of lambda and different analyzed features. The predictive performance of each signature was evaluated via receiver operating curve (ROC).
**Results:** Three different radiomic signatures were developed, respectively, based on 15 (λ=λmin), 9 (λ=1 SE) and 6 (λ=1.SE, venous phase only and no shape features) radiomic features. The signatures showed good discrimination between case and control patients with AUCs=0.96(0.85-1.00), p<0.001, SE=85%, SP=100% for the first signature; AUC=0.94(0.80-1.00), p<0.001, SE=80%, SP=100% for the second signature and AUC=0.89(0.73-0.95), p<0.001, SE=80%, SP=86% for the third signature. This last signature has slightly lower diagnostic performances, but it is more easily reproducible since it is only based on the venous phase.
**Conclusion:** Although the small sample number, our study suggests that a radiomic approach could be a significant predictor for recurrence in patients who had undergone OLT for HCC.

### SS 7.8

### CT texture analysis and RECIST 1.1 criteria: assessment of response to chemotherapy in colorectal liver metastases

F. Landolfi[1], D. Caruso[1], V. Forte[1], N. Panvini[2], L. De Maria[1], B. D'Arrigo[1], G. Guido[1], A. Laghi[1]; [1]Rome/IT, [2]Genoa/IT

**Purpose:** To evaluate CT texture parameters and their correlation with response evaluation criteria in solid tumors (RECIST) in patients with colorectal liver metastases, to predict response to chemotherapy.
**Material and methods:** Sixteen patients with colorectal liver metastases were prospectively enrolled. All patients underwent contrast-enhanced CT before and after chemotherapy. Response to therapy was assessed with a dedicated software (Mint Lesion) which automatically extrapolated the following texture parameters from target lesions: Skewness, kurtosis, mean value of positive pixel (MPP), uniformity of distribution of positive pixel (UPP), entropy, and uniformity. Changes in texture parameters over time were then correlated with response to therapy. P values < 0.05 were considered statistically significant.
**Results:** Six patients (37.5%) had partial response (PR), 10 patients (62.5%) had progressive disease. Mean follow-up time was 62.6 ± 7.8 days. Kurtosis and skewness decreased in patients with PR-partial responders (kurtosis: 3.17 ± 0.26 vs 2.95 ± 0.18; skewness: -0.02 ± 0.12 vs -0.12 ± 0.18, all P<0.05) while increased in patients with PD-progressive disease (kurtosis: 3.03 ± 0.96 vs 3.33 ± 0.05; skewness: -0.07 ± 0.08 vs -0.28 ± 0.35, all P<0.05). MPP, UPP, uniformity and entropy did not show significant differences before and after chemotherapy (all P > 0.05).
**Conclusion:** Kurtosis and skewness correlate with RECIST in patients with colorectal liver metastases. Such texture parameters may play an important role in the prediction of response to chemotherapy.

### SS 7.9

### Machine learning-based automated image registration improves reader confidence and lesion colocalization in cross-sectional liver studies

K.A. Hasenstab[1], G. Moura Cunha[1], T.I. Delgado[1], K. Wang[1], A. Higaki[1], S. Ichikawa[1], A. Schlein[1], R.L. Brunsing[2], A. Schwartzman[1], A. Hsiao[1], C.B. Sirlin[1], K. Fowler[1]; [1]La Jolla, CA/US, [2]Stanford, CA/US

**Purpose:** Variability in liver morphology, patient positioning and motion can impact lesion colocalization across different series or studies. We applied a fully automated registration algorithm to liver imaging studies to determine its impact on reader confidence and lesion colocalization.
**Material and methods:** This is a retrospective, cross-sectional study. From surveillance gadoxetate-enhanced liver MRIs, we randomly selected 100 within-patient inter-exam pairs (hepatobiliary phase series; baseline and follow-up), and independently applied manual image registration performed by expert readers and a fully automated algorithm, comprising both a machine learning-based liver segmentation and a 3D affine transformation network. Reader confidence on image feature similarities was analyzed using summary statistics. Colocalization was assessed through the distance of lesion centers on overlapping baseline and follow-up images using percentiles and paired t tests.
**Results:** Reader confidence improved across 49 pairs for reader 1 and 32 pairs for reader 2 after applying the automated registration. 84 and 73 pairs were classified with the highest confidence score when registered using the automated algorithm, compared to 40 and 46 using manual registration. Lesion colocalization significantly improved in comparison to manual registrations. Compared to reader 1 manual registration, mean lesion distance reduced by 6.47 mm (95% CI 3.87, 9.06; p<0.001), whereas for reader 2 mean reduction was 7.60 mm (95% CI 4.86, 10.34; p<0.001). Readers were not significantly different from each other across performance metrics.
**Conclusion:** Automated liver focused image registration improves reader confidence and lesion colocalization in comparison to manual registration, potentially improving clinical care when visual comparison is the standard as in surveillance or treatment response assessment.

### SS 7.10

*Withdrawn by the authors*

11:00 - 12:30 Room Bologna

*Scientific Session SS 8*

### Pancreatic neoplasms

### SS 8.1

### Quantitative CT-based radiomics of pancreatic ductal adenocarcinoma: a valuable tool for radiological staging?

M.H.A. Janse[1], G. Litjens[1], S. Zinger[2], P.H.N. de With[2], M. Prokop[1], J.J. Hermans[1]; [1]Nijmegen/NL, [2]Eindhoven/NL

**Purpose:** To investigate the value of quantitative radiomics to predict local resectability and presence of metastatic disease of pancreatic ductal adenocarcinoma (PDAC) on routine contrast-enhanced abdominal CT.

**Material and methods:** 89 patients diagnosed with stage I-IV PDAC in the head were included (m:f=47:42; range 45-84 yrs). First-order intensity, texture and shape features were calculated for manually 3D-segmented tumors. Segmentation of local vessels was not performed. Student's t test or the Mann-Whitney test were used to compare both locally advanced (n=36, 40%) and/or metastatic tumors (n=18, 20%) to resected tumors (n=42, 47%). A feature was considered significant with $p<0.05$. Using these features, two support vector machines (SVM) were trained: one to predict local resectability and one to predict the presence of distant metastases.

**Results:** Following an inter-observer analysis in eleven cases, 84/105 features (ICC >0.75) were selected for further analysis. Univariate analysis resulted in 24 features associated with locally advanced disease, and 10 features associated with metastatic disease. These features were used to predict local resectability and distant metastases using a SVM. For local resectability, this resulted in a sensitivity and specificity of 75% and 67%, with an area under curve (AUC) of 0.71, comparable to experts sensitivity and specificity of 60-96% and 22-76%. The prediction of metastatic disease resulted in a sensitivity and specificity of 40% and 60%, with an AUC of 0.51.

**Conclusion:** Quantitative CECT-based radiomics, based on tumor features only, is comparable to expert performance in predicting local resectability. Prediction of distant metastases is more challenging.

### SS 8.2

### Preoperative nomogram predicting malignancy in the patients with intraductal papillary mucinous neoplasm of the pancreas: focused on imaging features

J.A. Hwang[1], S.-Y. Choi[2], J.E. Lee[2], H.J. Park[3], J.H. Min[4], S. Lee[3], J.Y. Moon[3]; [1]Cheonan/KR, [2]Bucheon/KR, [3]Seoul/KR, [4]Daejeon/KR

**Purpose:** To identify the preoperative imaging and clinical features differentiating malignant from benign intraductal papillary mucinous neoplasm (IPMN) of the pancreas and develop a nomogram for estimating the individualized risk of malignant IPMN.

**Material and methods:** Seventy-four patients with benign IPMNs and 52 patients with malignant IPMNs who underwent contrast-enhanced CT or MR imaging were retrospectively evaluated in two tertiary institutions. Significant findings for malignancy were identified at univariate and multivariate analyses. Based on the significant variables in multivariate analysis, we developed a nomogram to predict malignancy in IPMNs and the performance of constructed nomogram was also evaluated.

**Results:** Multivariate analysis revealed that enhancing mural nodule (odds ratio [OR]=14.88, $p<0.001$), increased serum CA19-9 (OR=3.25, p=0.033), abrupt change in main pancreatic duct (MPD) caliber with distal pancreatic atrophy (OR=3.23, p=0.050), MPD diameter ≥ 5mm (OR=3.12, p=0.034) were independent significant findings to predict malignant IPMN. Among them, enhancing mural nodule showed a highest OR and a highest predictor point on the nomogram. The area under the receiver operating characteristic curve for the nomogram was 0.892. When all four parameters were satisfied, specificity for malignancy prediction was maximized up to 98.7% (73/74, 95% confidence interval: 92.7%, 100%).

**Conclusion:** Constructed nomogram using preoperative CT and MR imaging could be useful in the estimation of individualized risk for malignancy in patients with IPMN. Enhancing mural nodule, increased serum CA19-9, abrupt change in MPD caliber with distal pancreatic atrophy, dilated MPD, and their combinations may be helpful to predict malignant potential of IPMN.

### SS 8.3

### Differential diagnosis of focal pancreatic masses: autoimmune pancreatitis or adenocarcinoma? Diagnostic accuracy of CT and post-processing CT texture analysis

G. Cardano, A. Fighera, L. Frulloni, G.A. Zamboni, G. Mansueto; Verona/IT

**Purpose:** To identify useful morphological and texture analysis CT features for the differential diagnosis between pancreatic ductal adenocarcinoma (PDAC) and focal autoimmune pancreatitis (AIP).

**Material and methods:** Were reviewed the MDCTs performed to characterize solid pancreatic masses in 60 patients (30 focal AIP and 30 PDAC). Were evaluated lesion size and margins, main pancreatic duct (MPD) intralesional stenosis and upstream dilatation, upstream chronic pancreatitis and biliary dilatation. Attenuation was measured in the lesion and the unaffected parenchyma in the baseline, late-arterial, venous and delayed phases. Texture-analysis was performed using LIFEX software. Statistical analysis (t test, univariate and multivariate regression) was performed using SPSS.

**Results:** Significant differences were observed between AIP and PDAC ($p<0,05$) in lesion attenuation in the unenhanced, late-arterial and venous phase, in normal pancreas unenhanced attenuation, in biliary dilatation and in texture entropy in the delayed phase. In univariate logistic regression analysis, lesion attenuation in the unenhanced, late-arterial and venous phases was significant ($p<0.05$). "AIP risk features", namely upstream MPD dilatation, biliary dilatation and chronic pancreatitis were significant "PDAC risk features". In multivariate regression analysis, upstream chronic pancreatitis was a statistically significant predictor of PDAC. The whole statistic model showed a significant predictive value for the diagnosis of PDAC, with a COX-SELL $R2=0.422$; receiver operating characteristic (ROC) curve confirmed its accuracy (AUC=0.918, p=0.037).

**Conclusion:** We have identified several features that can improve the differential diagnosis between AIP and PDAC: higher lesion attenuation in the baseline, arterial and venous phases increase the probability of AIP, while upstream MPD dilatation, biliary dilatation and chronic pancreatitis increase the probability of PDAC.

### SS 8.4
### Histogram analysis of MR images: assessment of intra-tumoral heterogeneity and correlation with the biological behavior of pancreatic ductal adenocarcinoma

R. De Robertis[1], N. Cardobi[2], R. Negrelli[1], M. D'Onofrio[1], S. Montemezzi[1]; [1]Verona/IT, [2]Peschiera del Garda/IT

**Purpose:** To evaluate MR-derived whole-tumor histogram analysis parameters in predicting the aggressiveness of pancreatic ductal adenocarcinoma (PDAC).
**Material and methods:** Pre-operative MR images of 127 consecutive patients with PDAC were retrospectively analyzed. T1-/T2-weighted images and apparent diffusion coefficient (ADC) maps were analyzed. Histogram-derived parameters were compared to several pathological features (grade, vascular infiltration, and nodal metastases) using Mann-Whitney U test. Diagnostic accuracy was assessed by receiver operating characteristic area under curve (ROC-AUC) analysis; sensitivity and specificity were assessed for each histogram parameter.
**Results:** No significant differences were found among histogram parameters for the prediction of tumor grade. $ADC_{entropy}$ was significantly higher in PDACs with vascular involvement (p=.022; AUC=.641), with specificity of 92.2%. $ADC_{skewness}$ was significantly higher in PDACs with nodal metastases (p=.027; AUC=.642), with 72% specificity. No significant differences between groups were found for other histogram-derived parameters (p >.05).
**Conclusion:** Whole-tumor histogram analysis of ADC values is a valuable tool for predicting the aggressiveness of PDACs. Our results indicate that histogram metrics related to intra-tumor heterogeneity are the most accurate parameters for the identification of PDACs with higher biological aggressiveness.

### SS 8.5
### Prediction of malignant potential in intraductal papillary mucinous neoplasm of the pancreas: comparison between contrast-enhanced CT and MRI using Revised 2017 International Consensus Guideline

J.E. Lee[1], S.-Y. Choi[1], J.A. Hwang[2], J.H. Kim[3]; [1]Bucheon/KR, [2]Cheonan/KR, [3]Seoul/KR

**Purpose:** To evaluate the imaging and clinical features proposed by the Revised 2017 International Consensus Guideline for predicting malignant potential of pancreatic intraductal papillary mucinous neoplasms (IPMNs), and to compare the diagnostic performance and intermodality agreement between CT and MRI.
**Material and methods:** In this retrospectively study, two radiologists analyzed the preoperative CT and MRI of 86 patients with surgically resected pancreatic IPMNs (benign = 58, malignant = 28), according to the revised 2017 international consensus guideline. Based on the number of high-risk stigmata and worrisome features, a scoring system predicting the malignant potential of pancreatic IPMNs was developed. The diagnostic performance of CT and MRI for the prediction of malignant IPMNs were analyzed using ROC analysis. Intermodality agreement was also assessed.
**Results:** On both CT and MRI, enhancing mural nodule, main pancreatic duct (MPD) size, abrupt MPD caliber change with distal pancreatic atrophy, lymphadenopathy, and cyst growth rate were significantly larger and more common in malignant IPMNs than benign ($p < 0.05$). As for the clinical variables, elevated serum CA 19-9 level and pancreatitis were more common in malignant IPMNs than benign ($p < 0.05$). Diagnostic performance of CT (AUC = 0.832) and MRI (AUC = 0.857) for predicting malignant IPMNs was comparable without significant difference (p = 0.433). The intermodality agreement was good (k = 0.696) for the total score.
**Conclusion:** The Revised 2017 International Consensus Guideline was useful in identifying malignant IPMNs. The diagnostic performance for the prediction of malignant IPMNs was comparable between CT and MRI, and showed good intermodality agreement.

### SS 8.6
### Prediction of high-grade pancreatic neuroendocrine neoplasms differentiation with imaging

A. Azoulay, J. Cros, M.-P. Vullierme, V. Vilgrain, M. Ronot; Clichy/FR

**Purpose:** To evaluate the value of imaging including texture analysis for the assessment of tumor differentiation in high-grade pancreatic neuroendocrine neoplasms (G3pNEN).
**Material and methods:** Pretreatment CT and MRI examinations of patients diagnosed with G3pNENs between 2006 and 2017 were evaluated. Tumor differentiation was assessed following the 2017 World Health Organization (WHO) classification, separating well (G3pNETs) from poorly differentiated (G3pNECs) lesions. CT and MRI features including size, type of enhancement, inner bleeding, the presence of sub-diaphragmatic metastasis or lymph node were recorded. Additional computerized texture analysis (TexRad software) of lesions on arterial and portal phase CT was also performed. Chi-square or Fisher's exact tests, and Student's t or Mann-Whitnet tests were used.
**Results:** Thirty-seven G3pNENs (mean 60±46mm) were evaluated in 37 patients (21 men (57%), 16 women (43%), mean age 56±13). Twenty-three (62%) were G3pNECs and fourteen (38%) were G3pNETs. G3pNETs had a significantly smaller size (mean 42±24mm vs. 70±51mm, p=0.039), higher CT attenuation on pre-contrast (37±6HU vs. 30±4HU, p=0,002), and on portal venous phase (92±19HU vs. 75±18HU, p=0.014). The mean ADC value was lower in G3pNECs (1.1±0,1 vs. 1.4±0,2 $10^{-3}$ mm2/s, p=0.005). Computerized texture analysis showed that G3pNECs were more heterogeneous on portal venous phase (Ent 0: 4.7 ±0.2 vs. 4.5±0.4, p=0.023).
**Conclusion:** CT and MRI characteristics are helpful to assess the degree of differentiation of G3pNEN. The added value of computerized texture analysis remains uncertain but appears promising.

### SS 8.7
### CT texture analysis and quantitative imaging for the detection of local tumor recurrence in patients resected for pancreatic adenocarcinoma

A. Mazzaro, G.A. Zamboni, M. Chincarini, M.C. Ambrosetti, G. Mansueto; Verona/IT

**Purpose:** To compare the CT texture analysis and quantitative imaging features of local tumor recurrence and postoperative fibrosis in patients who underwent resection of pancreatic adenocarcinoma.
**Material and methods:** We selected 80 consecutive patients resected for pancreatic adenocarcinoma with an early detection of solid hypodense tissue in the surgical bed at the after-surgery follow-up CT. For all patients, further follow-up CTs were available. Two readers in consensus measured the solid tissue attenuation drawing a region of interest (ROI) in the late-arterial and venous phases. The absolute (HUven-HUart) and ratio of enhancement (HUven-HUart/HUart) were calculated. First-order statistics CT texture data (variance, skewness and kurtosis) were extracted in the venous phase ROI using Mazda software. Unpaired T test was used for statistical analysis.
**Results:** The further follow-up CTs revealed in 38/80 patients the development of local tumor recurrence and in 42/80 a post-surgical scar tissue stable in size and features. Mean absolute enhancement from arterial to venous phase at first CT was significantly lower in patients with recurrence compared to patients with fibrosis (9.7±0.5 HU vs 19.9±6.5 HU, p<0.0001). Similar results were obtained with enhancement ratio (recurrence: +22.8% vs fibrosis: +62.5%, p<0.0001). Mean kurtosis was significantly different between the two groups (recurrence: 0.459 vs fibrosis: 0.126, p<0,0001). No significant difference was found for variance (0.096 vs -0.057, p=0.67) and skewness (315.13 vs 296.70, p=0.55).
**Conclusion:** Local recurrence appeared to have poorer progressive enhancement than post-surgical fibrosis. Between CT texture analysis first-order statistical data, kurtosis seems to be an effective parameter to differentiate between recurrence and fibrosis.

### SS 8.8

### Impact of structured report on the quality of preoperative CT staging of pancreatic ductal adenocarcinoma

M. Dimarco, R. Cannella, S. Pellegrino, F. Allegra, D.G. Castiglione, F. Vernuccio, G. Brancatelli; Palermo/IT

**Purpose:** To test whether a structured report improves the quality of preoperative CT staging of pancreatic ductal adenocarcinoma (PDAC) compared to conventional report.

**Material and methods:** This retrospective single-center study included 27 patients (12 males, 15 females, mean age 64±11 years) with pathologically proven locally advanced PDAC operated between 2015 and 2018. All patients were imaged with contrast-enhanced CT with pancreatic staging protocol. Two readers (R1: advanced abdominal radiologist and R2: trainee in diagnostic radiology), blinded to the pathological diagnosis, independently evaluated CT images using a conventional free-text unstructured report and structured report in two different reading sessions four weeks apart. Readers scored the presence of morphologic tumor features (location, size, vascularity), as well as the vascular tumor contact and distant metastases. Chi-square exact test was used to compare the frequency of reported features between conventional and structured reports. The Cohen κ test was used to assess the intra- and inter-reader agreements. A $p<0.05$ was considered statistically significant.

**Results:** A total of 104 (54 conventional and 54 structured) reports were analyzed. Structured report showed significantly higher reporting of arterial and venous structure ($p\leq0.012$), suspicious hepatic ($p<0.001$), peritoneal ($p<0.001$) and lymph node ($p<0.001$) lesions. Involvement of the superior mesenteric artery was scored to be present in 37% of cases with structured report as opposed to 32% with conventional report ($p<0.001$). Intra-reader agreement ranged from 0.727 to 0.919.

**Conclusion:** The use of structure report may improve the quality of preoperative CT staging of PDAC compared to standard free-text unstructured report.

### SS 8.9

### Adenosquamous carcinoma vs. ductal adenocarcinoma of the pancreas: evaluation of CT and MRI features

K. Schawkat[1], A. Brook[1], A. Descovich-Garces[1], A.J. Moser[1], C. Decicco[1], T. Singer[1], J. Glickman[1], M. Manning[2], K.J. Mortele[1]; [1]Boston, MA/US, [2]Washington, DC/US

**Purpose:** To analyze key distinguishing features of pancreatic adenosquamous carcinoma (ASqC) and pancreatic ductal adenocarcinoma (PDAC) and compare the presence of ring enhancement on CT and MRI.

**Material and methods:** This retrospective study was approved by our institutional review board. Eight patients with treatment-naïve and biopsy-proven ASqC (six women, mean age: 62.9, range: 40-75) and 16 patients with PDAC (eight women, mean age: 67.4, range: 47-83), who underwent both contrast-enhanced CT and MRI were included. The presence of imaging features of ASqC and PDAC was evaluated on CT and MR. The confidence in describing the ring enhancement feature was rated on a 5-point Likert scale.

**Results:** In ASqC, the readers were significantly more confident in observing the key differentiating feature of ring enhancement using MRI than CT (confidence 1.71±0.49 vs. 0.88±0.35, p=0.017,) with an area under the ROC curve of 0.982 for MRI vs. 0.945 for CT. Significant differences between ASqC and PDAC were seen in tumor size, location, vascular infiltration, T2 signal intensity and presence of focal areas of necrosis on MRI ($p<0.001$, p=.037, p=.014, p=.028 and p=.0023, respectively).

**Conclusion:** MR depicts the key distinguishing feature ring enhancement in ASqC with a higher readers' confidence. MRI is also able to demonstrate other key features of ASqC (T2 signal intensity and presence of focal areas of necrosis) which could be additionally useful cues for diagnosing ASqC.

### SS 8.10

### Solid pseudopapillary neoplasms of the pancreas: clinicopathological and radiological features according to size, twenty-year experience from a high-volume center

A. Grecchi, R. De Robertis, A. Beleù, G. Rizzo, M. Catania, M.C. Ambrosetti, M. D'Onofrio; Verona/IT

**Purpose:** To analyze and correlate clinicopathological and radiological features of resected solid pseudopapillary neoplasms of the pancreas according to their size.

**Material and methods:** Clinicopathological and radiological features of resected solid pseudopapillary neoplasms (SPN) of the pancreas over a twenty-year period were retrospectively analyzed. For the purpose of statistical analysis, tumors were divided into three groups according to their size (≤30 mm, 31-50 mm, and >50 mm). Clinicopathological and radiological features were compared among groups using Kruskal-Wallis' and Fisher's exact tests.

**Results:** Between January 1997 and December 2017, the study population consisted of 106 patients, with a median age at diagnosis of 31 years (range 7–68). Patients with small tumors (≤30 mm) had a significantly higher age at diagnosis compared with the other groups of patients (p=.038). Large tumors (31-50 and >51 mm) were more frequently located in the pancreatic body/tail compared with tumors ≤30 mm (p=.008). No other significant differences were found between the clinicopathological features of the three groups of patients (all p>.05). Most tumors presented a mixed solid and cystic appearance (54.7%), with well-defined margins (87.7%). Tumors ≤30 mm were significantly more frequently entirely solid (53.8%) compared with larger tumors (p=.028). The rate of incorrect preoperative diagnosis was higher in tumors ≤30 mm compared with larger tumors, albeit without significant differences between groups (p=.561).

**Conclusion:** Malignancy in solid pseudopapillary neoplasms is not correlated with tumor size; tumors ≤30 mm may present atypical imaging features, which may overlap with those of other solid tumors of the pancreas.

**11:00 - 12:30** **Room Amalfi**

*Scientific Session SS 9*

### Colorectal diseases: CT colonography and beyond

### SS 9.1

### Locally advanced rectal cancer: can diffusion-weighted imaging and apparent diffusion coefficient predict complete response to chemo-radiotherapy?

F. Landolfi, F. Coi, I. Martini, E. Pilozzi, M.F. Osti, M. Ferri, A. Laghi, E. Iannicelli; Rome/IT

**Purpose:** To evaluate both qualitative and quantitative performance of diffusion-weighted magnetic resonance imaging (DWMRI) in response assessment of locally advanced rectal cancer (LARC) after neoadjuvant chemo-radiotherapy (CRT), to distinguish responders and non-responder patients and identify pathologic complete responders (CR).
**Material and methods:** 68 patients with LARC underwent MRI prior to and after CRT. For qualitative analysis, DWMR images were evaluated assessing MR-tumor regression grade (TRG); histopathological TRG was the reference standard. For quantitative analysis, tumor's apparent diffusion coefficient (ADC) values were measured pre-CRT and post-CRT. ΔADC (ADC post-ADC pre) was also calculated. The cut-off ADC values of the receiver-operating characteristics (ROC) curve were employed to establish the diagnostic performance of ADC to assess tumor response.
**Results:** A significant correlation between the TRG defined on DWMRI after CRT and pathological TRG was found (Spearman's rank correlation test: p value <0.001). The mean post-CRT ADC and ΔADC in responder patients were significantly higher compared to non-responder ones (Student's T test: p value <0.001). By qualitative analysis, responders were correctly identified in 85% of cases. CR was identified in 56% of cases; integrating quantitative analysis using a cut-off value of ADC post-CRT of $1.23 \times 10^{-3}$ $mm^2/s$ sensitivity and specificity were 65% and 80%, respectively.
**Conclusion:** Through both qualitative and quantitative analyses of DWMRI, MRI enables LARC response assessment after CRT, also resulting in a valid tool in CR prediction.

### SS 9.2

### Polyp detection rate as a quality measure in CT colonography: analysis of the performance of a CT colonography service using a reduced bowel preparation without dietary restriction

S. Vicini[1], M. Rengo[1], F. Tiberia[1], D. Bellini[1], G. Trionfera[2], I. Carbone[1], A. Laghi[3]; [1]Latina/IT, [2]Valmontone/IT, [3]Rome/IT

**Purpose:** To evaluate the performances of a CT colonography (CTC) service using polyp detection rate (PDR) as a quality measurement. To stratify results according to patient's age, gender and symptoms.
**Material and methods:** We retrospectively analysed 1446 consecutive patients who underwent CTC from July 2015 to September 2018. In all patients, a reduced bowel preparation (100 g of Macrogol and 60 ml of hyperosmolar iodinated contrast media for fluid tagging) and no dietary restriction were administered the day before the examination. PDR was calculated, considering only polyps ≥ 6 mm, for the entire population and after the stratification in sub-groups according to age (< 65 vs ≥ 65 yy), gender (male vs female) and symptoms (asymptomatic vs symptomatic). All positive patients at CTC were subjected to endoscopy to confirm the presence of polyps. Polyps were scored according to the location, dimension and quality of the bowel preparation. Differences between sub-groups were evaluated with Chi-square test.
**Results:** In total, 1.446 patients (627/819 M/F, mean age 62.45 ± 14.22 years) were analysed. Bowel preparation was optimal in 1392 patients (96.3%). PDR of total population was 9.19% (133/1446). PDR was significantly higher in older (10.93% vs 7.54%, P = 0.025) and male patients (11.32% vs 7.57%, P = 0.014). PDR was not significantly different between symptomatic and asymptomatic patients (11.29% vs 8.16%, P = 0.052).
**Conclusion:** CTC without diet restriction for bowel preparation is effective in detecting colorectal polyps, with PDR comparable to those previously reported in the literature.

### SS 9.3

### Detection rate for advanced neoplasia of single-round CTC vs. three fecal immunochemical test rounds in population screening of colorectal cancer: the SAVE randomized trial

L. Sali, L. Ventura, M. Mascalchi, P. Mantellini, M. Zappa, G. Grazzini; Florence/IT

**Purpose:** To compare a single-round of CTC versus three biennial rounds of fecal immunochemical test (FIT) for colorectal cancer (CRC) screening.
**Material and methods:** In this pragmatic randomized trial, 14981 subjects aged 54 to 65 years, living in a district of Florence, Italy, were randomized (1:2) and invited by mail to one of the two screening interventions: 1) single-round CTC; 2) three biennial rounds of FIT. Main outcome was detection rate for advanced neoplasia, defined as the proportion of invitees to screening intervention with screen-detected cancer or advanced adenoma over total number of invitees. Secondary outcome was positive predictive value (PPV) for advanced neoplasia.
**Results:** Participants to single CTC were 1286/4825 (26.7%) subjects whereas participants to first, second and third FIT rounds were 4677/9288 (50.4%), 4709/8676 (54.3%) and 4215/7914 (53.3%) subjects. Detection rate for advanced neoplasia of CTC (1.4%; 95%CI 1.1-1.8) was lower than the cumulative detection rate of three FIT rounds (2.0%; 95%CI 1.7-2.3) [p=0.009]. However, PPV of single CTC (53.2%; 95%CI 44.1-62.1) was higher than that of three FIT rounds (32.3%; 95%CI 28.5-36.3) [p<0.001].
**Conclusion:** For population screening of CRC, a strategy based on single CTC yields a lower detection rate per-invitee than three biennial FIT rounds, but is associated with less unnecessary diagnostic colonoscopies.

### SS 9.4

### Role of CTC in differentiating sigmoid cancer versus chronic diverticular disease

R. Valletta[1], N. Faccioli[1], M. Tagliamonte[1], M. Bonatti[2], E. Santi[1], F. Lombardo[2], G. Mansueto[1]; [1]Verona/IT, [2]Bolzano/IT

**Purpose:** To retrospectively evaluate morphological findings of chronic diverticular disease and sigmoid carcinoma in CTC to differentiate them.
**Material and methods:** We included in our IRB-approved retrospective study 133 consecutive patients with histologically proven chronic diverticular disease (n = 77) or sigmoid carcinoma (n = 56). Two radiologists retrospectively analyzed CTC studies without awareness of the histological diagnosis. One reader scored each exam according to the presence or absence of potential discriminators (length, wall thickness, shouldering phenomenon, thickening type, growth pattern, diverticula, fascia thickening, fat tissue edema, loco-regional lymph nodes, mucosal pattern).
**Results:** The findings that suggest sigmoid carcinoma diagnosis were absence of diverticula in the affected segment (sensitivity 87.9%; specificity 90.5%); straight growth pattern (sensitivity 71.4%; specificity 90.9%); shouldering phenomenon (sensitivity 90.5%; specificity 81.8%); complete distortion of mucosal folds (sensitivity 95.2%; specificity 75.8%). Considering mass-like lesions, growth pattern and mucosal folds distortion lose their diagnostic value. The only morphological finding with higher diagnostic value is the absence of diverticula in the examined segment; its combination with shouldering phenomenon increases carcinoma diagnosis specificity.
**Conclusion:** Carcinoma is best differentiated from chronic diverticular disease in CTC by the absence of diverticula in the affected segment and the presence of shoulder phenomenon.

### SS 9.5

### Effectiveness of bowel preparation without diet restriction for optimal quality CTC: a validation study

F. Tiberia[1], M. Rengo[1], S. Vicini[1], D. Bellini[1], G. Trionfera[2], I. Carbone[1], A. Laghi[3]; [1]Latina/IT, [2]Valmontone/IT, [3]Rome/IT

**Purpose:** The aim of our study was to investigate whether bowel preparation without diet restriction for CTC is sufficient to reach the proposed minimum standard rate of adequate bowel preparation (>90%) and to identify any influencing factors in our population.
**Material and methods:** In our retrospective study, we enrolled 1446 patients (mean age 62.45+- 14.22 years, 627 men) who underwent CTC after bowel preparation with 100 g of macrogol, 60 ml of sodium diatrizoate and meglumine diatrizoate solution, for fluid tagging, the day before the examination. No dietary restrictions were applied. Quality of bowel preparation was assessed with a previously validated 4-point Likert scale evaluating, on a per segment basis, the quantity of tagging and degree of distention. We performed a subgroup analysis to examine the impact of the following factors on bowel preparation: age (>65 vs <65 years) and diverticular disease.
**Results:** Optimal tagging and distention were rated in 1392 (96.26 %) and 1428 (98.75% ) patients, respectively, resulting in optimal quality of preparation for 1409 (97.44%) patients. Diverticular disease was associated with lower incomplete distention rates (0.7% vs 1.98%, p=0.03) and lower quality of fluid tagging (4.6% vs 2.4%, p=0.03). Patient's age did not affect both tagging (3.5% vs 3.9%) and distention (0.8% vs 1.7%) (p=0.63 and p=0.12, respectively).
**Conclusion:** Our proposed preparation without dietary restriction is effective in providing optimal fluid tagging and bowel distention for optimal quality CTCs. Diverticula disease negatively affects the quality of fluid tagging and degree of bowel distension.

### SS 9.6

### Patient preferences for whole-body MRI or conventional staging pathways for colorectal cancer: a discrete choice experiment

S.A. Taylor, A. Miles, R. Evans, S. Morris, S. Halligan; London/UK

**Purpose:** To determine the importance placed by patients on attributes associated with whole-body MRI (WB-MRI) and standard cancer staging pathways, and ascertain drivers of preference.
**Material and methods:** Patients recruited to two multi-centre diagnostic accuracy trials comparing WB-MRI with standard staging pathways in lung and colorectal cancer were invited to complete a discrete choice experiment (DCE), choosing between a series of alternate pathways in which 6 attributes (accuracy, time to diagnosis, scan duration, whole body enclosure, radiation exposure, total scan number) were varied systematically. Data were analysed using a conditional logit regression model and marginal rates of substitution computed. The relative importance of each attribute and probabilities of choosing WB-MRI-based pathways were estimated.
**Results:** 138 patients (mean age 65, 61% male, lung n=72, colorectal n=66) participated (May 2015 to September 2016) in this study. Colon cancer patients valued accuracy most highly, followed by time to diagnosis, radiation exposure and number of scans. Patients were willing to 0.45 weeks for a 1% increase in pathway accuracy. Patients preferred WB-MRI-based pathways (probability 0.66 if they were equivalent in accuracy, total scan number, and time to diagnosis compared to a standard staging pathway).
**Conclusion:** Staging pathways based on first-line WB-MRI are preferred by the majority of patients if they at least match standard pathways for diagnostic accuracy, time to diagnosis and total scan number.

### SS 9.7

*Withdrawn by the authors*

### SS 9.8

### Diagnostic accuracy of MDCT in the assessment of mesorectal fascia invasion: comparison with MRI

C. Maino, D. Ippolito, C. Talei Franzesi, S.G. Drago, A. Pecorelli, S. Sironi; Monza/IT

**Purpose:** To assess the diagnostic accuracy of MDCT, compared with conventional MRI, in identifying mesorectal fascia (MRF) invasion in rectal cancer patients.
**Material and methods:** We retrospectively evaluated 120 patients with biopsy-proven rectal cancer who underwent a whole-body CT staging and pelvic MRI examination. The contrast-enhanced MDCT scans were performed on a 256-row scanner (iCT Elite, Philips) and images were reviewed as axial and as multiplanar reconstructions along the rectal tumor axis. MRI studies were performed on 1.5 T scanner (Achieva, Philips) with dedicated phased-array, including multiplanar T2 and axial T1 sequences and diffusion-weighted images (DWI). Axial and MPR CT images independently were compared to MRI to evaluate the involvement of MRF. Data were analyzed to compare the diagnostic performance of MDCT and MRI.
**Results:** According to MRI, the MRF was involved in 67 patients and not involved in 53 patients. The number of patients correctly staged by the native axial CT images was 100 out of 120 (61 with involved MRF; 29 with not involved MRF), while using the MPR, 112 patients were correctly staged (66 with involved MRF; 46 with not involved MRF). Local tumor staging suggested by MDCT agreed with those of MRI, obtaining for CT axial images an accuracy 78%, while MPR images showed higher diagnostic accuracy (90%), in terms of MRF involvement.
**Conclusion:** New-generation CT, using high-resolution MPR images, represents a diagnostic tool in the assessment of loco-regional and whole-body staging of advanced rectal cancer, especially in patients with MRI contraindications.

### SS 9.9

### Analysis of anatomic variants of superior mesenteric artery and vein using MDCT

S. Pashapoor[1], K. Atasoy[2]; [1]Bursa/TR, [2]Ankara/TR

**Purpose:** To give a preoperative description of the arterial and venous anatomy of the colon on MDCT.
**Material and methods:** Colic branches of the superior mesenteric artery (SMA) and vein (SMV) were studied in 100 patients who underwent abdominal MDCT for various clinical reasons. To reduce radiation burden, a single CT scanning was employed in each patient which allowed opacification of both the arterial and venous structures owing to a peculiar technique where the contrast medium was injected in two separate boluses.
**Results:** The incidence of colic arteries arising from the SMA was ileocolic artery, 100%; right colic artery, 25%; middle colic artery, 97%; and accessory middle colic artery, 20%. All patients had a single ileocolic vein, which drained into the SMV in 95 cases, into the gastroomento-pancreaticoduodeno-colic trunk (GPCT) in 3 cases, into ileal trunk in 1 case and into accessory MCV in 1 case. The GPCT was detected in 57 cases with several forms of the origin of the respective branches: the gastroomento-pancreaticoduodenal trunk (GPT) was detected in 34 cases, and the classic GCT was in 8 cases. IMV joined to the splenic vein in 50% of patients.
**Conclusion:** Although the vascular anatomy of the colon is variable and complex, preoperative 3D-CT is informative and very helpful for surgeons in colonic resections. Both the arteries and veins of the colon can be opacified with a single CT scanning via injecting contrast medium in two separate boluses, without increasing patient radiation.

### SS 9.10

### Hydrostatic reduction of intussusception in pediatric age group: can failure be predicted?

B. Agridag Ucpinar, B. Keles, S. Durmaz, E. Camurcuoglu, Z. Kazci, E. Ozmen, S.M. Erturk; Istanbul/TR

**Purpose:** To correlate factors including patient sex, age and presence of an organic cause with the success rate of US-guided hydrostatic reduction in pediatric patients with intussusception.
**Material and methods:** A total of 64 patients (42 male, 22 female; age: 30.9±27.6 months) underwent hydrostatic reduction. Patients were grouped as those in whom the reduction was successful (success group) or failed (failure group). Percentages were given with 95% confidence intervals (CIs). Regarding the patient age, the groups were compared using Student's t test. Regarding the patient sex and the presence of an organic cause, the groups were compared using Fisher's exact test. p values smaller than 0,05 were considered statistically significant.
**Results:** Regarding the patient age, there was no statistically significant difference between the success (n=51; 35.8±8.6 months) and failure groups (n=13; 29.7±3.8 months; p=0.521). Regarding the gender, although there was no statistically significant difference, the female ratio was higher in the failure group (success: 36 male,15 female; failure: 6 male, 7 female; p=0.114). In six patients, there was an organic cause (Meckel diverticulum (n=3), appendicitis (n=2), Burkitt lymphoma (n=1)). The reduction failed in all six patients with an organic cause (6/6; 100% [95%CI=60.9%-100%]) and in seven of 58 patients without (7/58; 12,1% [95%CI=5.9%-22.8%]; p<0.00001).
**Conclusion:** In hydrostatic reduction of intussusception in pediatric patients, presence of an organic cause is an important factor that negatively affects the outcome. Thus, a detailed pre-reduction ultrasound examination is needed to rule out it. Although it was statistically not significant in this study, female sex may contribute to the failure rate.

**11:00 - 12:30** **Room Milan**

*Scientific Session SS 10*

### Modern imaging of gastro-pancreatic diseases

### SS 10.1

### Short MRI follow-up protocol in the evaluation of hepatic metastases from gastroenteropancreatic neuroendocrine tumors

Z. Aleksander-Markuszewska[1], L. Funicelli[2], V. Giannetta[2], R. Labruna[2], N. Fazio[2], M. Bellomi[2]; [1]Warsaw/PL, [2]Milan/IT

**Purpose:** To assess the value of hepatobiliary phase (HBP) gadoxetic acid-enhanced magnetic resonance imaging (MRI) as a single-phase protocol in the follow-up evaluation of hepatic metastases of gastroenteropancreatic neuroendocrine tumors (GEP-NETs).
**Material and methods:** We retrospectively reviewed our institution's medical records of 338 patients diagnosed with liver metastases of GEP-NETs, from 2000 to 2018 identifying 43 patients with pancreatic (n = 22) and ileal (n =21) origin who underwent at least two gadolinium ethoxybenzyl diethylenetriamine pentaacetic acid (Gd-EOB-DTPA) enhanced MR examinations that included 20-min delayed hepatobiliary phase imaging. Two radiologists independently evaluated two sets of MRI exam of each of the patient-basic and follow-up exam using RECIST 1.1 categorization and classified them into one of the three categories: response, stable and progressive disease. For the follow-up exam, one radiologist reviewed the complete exam, the second-one disposed only of Gd-EOB-DTPA-enhanced 20-min hepatobiliary phase images. We compared RECIST evaluation of the two readers.
**Results:** There was a good agreement between the two reviewers (97%) with only 1 discordance in RECIST categorization (stable vs. progressive disease). Of the 43 patients included in the study, 5 were excluded because of the incomplete examination. Of the 38 evaluated patients, 15/16 were categorized as progressive, 22/21 as stable disease and 1 as a partial response.
**Conclusion:** In conclusion, hepatobiliary phase images obtained after gadoxetic acid-enhanced dynamic MRI have a good diagnostic accuracy in assessment of GEP-NETs' hepatic metastases. Gd-EOB-DTPA hepatobiliary phase has a potential value as a single- phase short MRI protocol for the mid-term follow-up in this group of patients.

### SS 10.2

### The value of diffusion-weighted imaging in dynamic monitoring to chemotherapy effectiveness of advanced gastric carcinoma

J. Xu; Xi'an/CN

**Purpose:** To evaluate the value of diffusion-weighted imaging (DWI) and apparent diffusion coefficient (ADC) value in monitoring the chemotherapy effectiveness of advanced gastric carcinoma dynamically.
**Material and methods:** 42 advanced gastric carcinoma patients who were histopathologically confirmed underwent T2WI and DWI examinations at pre-chemotherapy, post-chemotherapy 3 d, 7 d, 30 d and 60 d, respectively. The longest diameters of tumor pre-chemotherapy and post-chemotherapy 60 d were measured on axial T2WI. Meanwhile, the ADC values at different time points were calculated. The mean ADC value among pre- and post-chemotherapy of each group (PR and SD) was compared.
**Results:** The ADC value of PR group increased gradually. The mean ADC value before therapy was statistically lower than those at different time points post-chemotherapy (P < 0.05). The ADC value of SD group increased gradually from pre-chemotherapy to post-chemotherapy 30 d, and then the ADC value decreased at post-chemotherapy 60 d. The differences in the mean ADC values at different time points were statistically significant (P < 0.05).
**Conclusion:** DWI and ADC value can dynamically, quantitatively and early detect and monitor the chemotherapy response of advanced gastric carcinoma.

Scientific Sessions

Authors' Index

### SS 10.3

### Predictive value of metastatic CT findings at initial diagnosis for overall survival in stage IV gastric cancer

L. Soydan, A.A. Demir, B.B. Oven Ustaalioglu; Istanbul/TR

**Purpose:** Metastatic gastric cancer (GC) is associated with poor prognosis. This study examined the size, countable number and extent of nodal and distant metastatic sites on CT images obtained at initial diagnosis in stage IV GC patients and analyzed if and how they can predict overall survival.
**Material and methods:** This retrospective study included 55 subjects diagnosed with stage IV GC. In addition to the retrieval of demographical, clinical and up-to-date survival data, baseline CT images obtained at the time of initial admission were reviewed for the extent of metastatic disease and prognostic factors on survival were analyzed by Cox proportional hazard models.
**Results:** None of the patient or tumor characteristics including age, gender, histological type, differentiation, location, and C-erbB status was found to significantly influence overall survival (p>0.05 for all). Multivariate analysis identified the number of perigastric and para-aortocaval metastatic lymph nodes as significant independent predictors of poor overall survival. Presence of metastasis in 7 to 14 perigastric lymph nodes (OR 4.0; 95% CI: 1.5-11.1; p=0.007) and presence of more than two metastatic para-aortocaval lymph nodes (OR 2.4; 95% CI: 1.1-5.5; p=0.034) were associated with significantly higher mortality risk.
**Conclusion:** The number of enlarged perigastric and paraaortic lymph nodes at initial CT examination seems to predict overall survival in patients with metastatic GC. However, large-scale studies are needed to generalize such a conclusion, which may provide the basis for alternative therapies other than palliation for those with a better outcome expectancy.

### SS 10.4

### Risk assessment for pancreatic fistula after pancreaticoduodenectomy with preoperative CT

R. Menghini, G.A. Zamboni, A. Cybulski, R. Valletta, G. Mansueto; Verona/IT

**Purpose:** To evaluate the predictive value of preoperative CT features for the risk of postoperative pancreatic fistula.
**Material and methods:** We included 74 patients who underwent preoperative MDCT and pancreaticoduodenectomy in our institution. The patients were divided into 2 groups according to clinical data: 37 patients with postoperative pancreatic fistula (POPF) (group A) and 37 patients without POPF (group B). One reader reviewed the CTs and measured at the planned resection plane the main pancreatic duct (MPD) diameter, the density of the parenchyma in the different enhancement phases and the parenchymal thickness. The difference in attenuation between the venous and arterial phase was calculated as a surrogate for parenchymal fibrosis. Body composition was analysed by calculating visceral adipose tissue area (VAT), subcutaneous adipose tissue area (SAT), and skeletal muscle area at the L2 level using ImageJ software. Retrorenal fat thickness and psoas density were also measured. Fisher's exact test was used for categorical variables and Student's t test for continuous variables.
**Results:** MPD diameter was 2.8±2.9 mm in group A and 6.5±3.2 mm in group B (P<0,0001). The mean attenuation difference between venous and arterial phase was -15.30 hounsfield units (HU) in group B and 0.70 HU in group A (P=0.0015). SAT was 17497.5 $mm^2$ in group A and 13292 $mm^2$ in group B (P=0.046). No significant difference was observed for the other parameters.
**Conclusion:** Fibrosis, expressed by increasing enhancement of the normal pancreatic parenchyma at the planned resection plane, MPD diameter and increased SAT may express increased risk for pancreatic fistula after pancreaticoduodenectomy.

### SS 10.5

### A 12-year experience of benign pancreatic hyperenzymemia: spectrum of secretin-MRCP findings

C. Gullì[1], A. Marrazzo[1], G. Restaino[2], M. Missere[2], E. Cucci[2], G. Sallustio[2]; [1]Rome/IT, [2]Campobasso/IT

**Purpose:** Benign pancreatic hyperenzymemia (BPH) is a condition characterized by a long-term increase of serum pancreatic enzymes without clinical evidence of organic disease. The aim of this study was to investigate the frequency of findings at MRCP with secretin stimulation (s-MRCP) in patients with BPH.
**Material and methods:** 252 patients (mean age: 51 ± 14 y/o; M:F= 133:119) who underwent s-MRCP at our Institution with clinical indication of BPH without previous diagnosis of pancreatic disease, from February 2007 to November 2018.
**Results:** In all, 172 (68%) subjects had an abnormal increase of lipase and amylase, 57 (22%) of amylase and 23 (9%) of lipase. A normal s-MRCP was observed in 60 patients (23%). Chronic pancreatitis was detected in 168 (65.7%) subjects, of whom 108 were mild and 60 moderate according to the Cambridge classification. Pancreas divisum was identified in 24 subjects (9.5%). Sphincter of Oddi dysfunction (SOD) and acinar filling were observed in 80 (31.7%) and 24 (9.5%) subjects, respectively; pancreatic exocrine function was normal in 96.7% of patients and mildly impaired in 3.3%. Cysts < 5 mm and > 5 mm were identified in 10 (4%) and 14 (5.5%) subjects, respectively; 14 patients (5.5%) needed follow-up for branch-duct intraductal papillary mucinous neoplasm.
**Conclusion:** The commonest s-MRCP finding in patients with BPH was mild/moderate chronic pancreatitis (65.7%); the other most frequent anomalies were SOD (31.7%) and acinar filling (9.5%). Based on our experience, s-MRCP is to be recommended in the diagnostic workup of patients with BPH.

### SS 10.6

### Evaluation of pancreatic fibrosis with gadolinium ethoxybenzyl diethylenetriaminepentaacetic acid-enhanced MRI

D. Yunaiyama, H. Yamaguchi, Y. Nagakawa, T.L. Harada, T. Nagao, K. Saito; Tokyo/JP

**Purpose:** To evaluate radiological-pathological correlation in pancreatic fibrosis between gadolinium ethoxybenzyl diethylenetriaminepentaacetic acid (Gd-EOB-DTPA)-enhanced MRI and resected non-tumorous tissue.
**Material and methods:** From January 1,2011 to April 30, 2018, those who underwent pancreatic surgery and Gd-EOB-DTPA-enhanced MRI in our hospital were analyzed. Poor study because of breath-hold discipline, discrepancy of TR/TE between each dynamic study phase, severe susceptibility artifacts and patients who suffered from pancreatitis at the examination were excluded. Patient's age, sex, with or without diabetes mellitus were analyzed. The value of apparent diffusion coefficient (ADC) and intensity of pre-dynamic study, portal phase, venous phase, hepatobiliary phase and each ratio with pre-dynamic study were analyzed by 2 diagnostic radiologists trained 8 years and 6 years, respectively. The degree of pancreatic fibrosis was categorized into 4 grades: none, mild, moderate and severe by a well-trained pathologist.
**Results:** Totally, 142 patients who underwent pancreatic surgery were analyzed in this study. The median age was 69 years (range: 40-88). Ninety-six patients were male and 46 were female, respectively. The degree of pancreatic fibrosis was associated with the intensity of pre-contrast (p=0.037), portal pre-contrast ratio (p=0.001) and venous pre-contrast ratio (p=0.001) with Kruskal-Wallis test; however, was not associated with the value of ADC (p=0.09). The degree of pancreatic fibrosis was prone to be associated with the necessity of percutaneous drainage; however, there was no statistical significance.
**Conclusion:** Dynamic MRI using Gd-EOB-DTPA can predict the degree of pancreatic fibrosis with the intensity of pre-contrast, portal pre-contrast ratio and venous pre-contrast ratio.

### SS 10.7

*Withdrawn by the authors*

### SS 10.8

**Diffusion-weighted imaging and dynamic MRI in the study of intraductal papillary mucinous neoplasms: pathological comparison with tumor grade correlation**

G. Giannotti, G. Tedesco, A. Beleù, G. Rizzo, N. Cardobi, R. De Robertis, M. D'Onofrio; Verona/IT

**Purpose:** Evaluation of mural nodules (MNs) and septa of intraductal papillary mucinous neoplasms (IPMNs) through diffusion-weighted imaging (DWI) and contrast-enhanced magnetic resonance (CE-MR) for the characterization of tumor grade.

**Material and methods:** 91 patients with pancreatic IPMNs were included. All patients had histological diagnosis after surgical resection and a pre-operative MR exam. There were 57 patients with IPMN with low-grade dysplasia and 34 patients with IPMN with high-grade dysplasia. MR exams were retrospectively reviewed by two radiologists. A third experienced radiologist directed conflicting analyses. The presence, number, size of solid nodules, enhancement, signal restriction in DWI sequences, size of the lesion and caliber of Wirsung duct were evaluated. Qualitative imaging characteristics have been compared to histological results. Cohen K coefficient was calculated to evaluate interobserver agreement.

**Results:** Statistically significant difference ($p<0.0001$) was between nodules >5mm with enhancement and signal restriction in DWI and tumor dysplasia, between size and number of nodules detected by MRI and the grade of dysplasia and between dilatation of the Wirsung duct and grade of dysplasia. ADC map showed statistically significant results ($p<0.0001$) in the correlation between the entropy parameter and the grade of dysplasia. No statistically significant difference was between nodules <5mm detected by MRI and the pathological analysis and between nodular dimensions and tumor dysplasia.

**Conclusion:** MRI with dynamic study and DWI sequences is an accurate method in the identification of solid nodule with cut-off of 5 mm. DWI sequences and dynamic study with contrast media can be useful in the identification of lesional malignancy. Entropy could be used as a predictive parameter of lesional malignancy.

### SS 10.9

**Preoperative imaging evaluation after downstaging of pancreatic ductal adenocarcinoma: a multi-center study**

A. Beleù, G. Rizzo, N. Bellini, A. Grecchi, I. Testa, G. Giannotti, N. Cardobi, R. De Robertis, M. D'Onofrio; Verona/IT

**Purpose:** Evaluation of pancreatic ductal adenocarcinoma (PDAC) after chemoradiotherapy downstaging is challenging due to CT overestimation of tumor extension and residual vascular involvement. With this study, we wanted to assess which radiological findings are most reliable at pre-operative imaging to achieve complete resection.

**Material and methods:** We retrospectively enrolled 71 patients with locally advanced and borderline-resectable PDAC who underwent neoadjuvant chemoradiotherapy. Pre-operative CT or MR has been evaluated by three radiologists to assess major qualitative and quantitative parameters of lesions. Accuracy, sensibility and specificity compared to anatomopathological results were evaluated. Cohen's K coefficient has been calculated to evaluate inter-observer agreement (IOA). Different dimensional cut-offs were tested and compared to anatomopathological diameter, tumor persistence and margin infiltration.

**Results:** 25 mm cut-off was 67% sensitive, 90% specific and 77% accurate in assessing real tumor dimension. Sensitivity and specificity for 25 mm cut-off were, respectively, 23% and 94% for margin infiltration, and 51% and 67% for tumor persistence. The imaging presence of perivascular cuff reported a low accuracy in determining tumor persistence and margin infiltrations. Lesion enhancement and pattern homogeneity were not accurate in determining tumor persistence. IOA was generally poor to fair, except for 25 mm cut-off classification where IOA was moderate. Diagnostic accuracy is superior in consensus lecture rather than single lecture.

**Conclusion:** Imaging methods tend to underestimate PDAC resectability after neoadjuvant-CRT. IOA is poor to fair in evaluating most of the qualitative parameters of downstaged pancreatic adenocarcinoma. Surgery should be considered for downstaged borderline resectable PDACs, independently of perivascular cuff presence, especially for tumors smaller than 25 mm.

### SS 10.10

**CT texture variables for the prediction of pancreatic fistula after pancreaticoduodenectomy**

R. Valletta[1], G.A. Zamboni[1], M.C. Ambrosetti[1], M. Bonatti[2], F. Lombardo[2], G. Mansueto[1]; [1]Verona/IT, [2]Bolzano/IT

**Purpose:** To investigate the CT texture variables for the prediction of onset of clinically relevant postoperative pancreatic fistulas (CR-POPF) after pancreaticoduodenectomy.

**Material and methods:** We included in our IRB-approved retrospective study 56 consecutive patients with histologically proven pancreatic adenocarcinoma, pancreatic neuroendocrine tumors (pNET) or pancreatic cystic neoplasm who underwent pre-operative contrast-enhanced computed tomography (CECT) before pancreaticoduodenectomy. 28 patients developed a pancreatic fistula after surgery and 28 had no post-surgery complications. CT texture analysis was used to quantify the heterogeneity of the pancreatic parenchyma on pre-operative CECT of both groups and the results were compared with the Mann-Whitney U test.

**Results:** Several texture features were significantly different between patients who developed CR-POPF and those who did not: kurtosis ($p = 0.011$), entropy $\log_2$ ($p = 0.019$) and energy ($p = 0.038$) as for the first-order features, all the gray-level co-occurrence matrix (GLCM) features (homogeneity, energy, contrast, correlation, entropy and dissimilarity) ($p<0.05$), several of the gray-level run length matrix (GLRLM) (SRE, LRE, HGRE, SRHGE, LRHGE, RP) and of the gray-level zone length matrix (GLZLM) features (SZE, HGZE, SZHGE, LZLGE, GLNU, ZLNU, ZP) ($p<0.05$).

**Conclusion:** Texture analysis of the pancreatic parenchyma on preoperative CECT was able to predict the onset of a post-operative pancreatic fistula. If confirmed on larger series, texture analysis could provide an additional means of preoperative risk stratification and possibly modify the management of these patients.

**11:00 - 12:30** **Room Florence**

*Scientific Session SS 11*

### GI tract: inflammatory disease and beyond

### SS 11.1

### Comparison of a simplified MR index of activity with and without gadolinium for assessing luminal disease and therapeutic response in patients with Crohn's disease

J. Rimola, N. Capozzi, A. Fernandez-Clotet, I. Alfaro, M. Masamunt, I. Ordás, J. Panes; Barcelona/ES

**Purpose:** To compare the diagnostic accuracy of a simplified MR index with and without the use of gadolinium-enhanced sequences for detecting activity and severe lesions and the therapeutic response in Crohn's disease (CD).
**Material and methods:** This study involves the prospective inclusion of patients with CD with at least one intestinal segment presenting severe inflammation at endoscopy (Crohn's Disease Endoscopic Index of Severity (CDEIS)>8.5 or presence of ulcers), requiring treatment with a biological drug. The simplified Magnetic Resonance Index of Activity MaRIA (sMaRIA), which includes wall thickness>3mm, edema, ulcers and fat stranding, was used to score the inflammation in each segment of the colon and terminal ileum. The sMaRIA was read first using T2-weighted sequences (T2w), and re-read after 1 month using the same sequences plus gadolinium-enhanced sequences (T2w+Gd). The accuracy of the different reads for assessing activity, severity and therapeutic response using endoscopy as the gold standard was compared using McNemar test.
**Results:** Data from 46 patients (254 intestinal segments; 57 with ulcers) at baseline and from 40/46 patients (209 segments) at treatment week 46 were available. For detecting active disease (sMaRIA ≥1), T2w had similar sensitivity than T2w+Gd (88% vs. 82% p=0.68) but was superior to T2w+Gd for specificity (94 vs. 85%, p=0.005) and overall accuracy (91% vs. 86%; p=0.02). For detecting severe inflammation at segment level (sMaRIA≥2) and for detecting therapeutic response, the sensitivity, specificity and overall accuracy of T2w and T2w+Gd were similar (p>0.05).
**Conclusion:** We have not observed any benefit on adding gadolinium-enhanced sequences on the use of sMaRIA for assessing luminal disease and therapeutic response in the patients with CD.

### SS 11.2

### Simultaneous T1 and T2 mapping for bowel wall imaging in Crohn's disease with MR fingerprinting

V. Obmann[1], N. Seyfried[2], A. Panda[3], W.-C. Lo[2], Y. Jiang[2], K. Wright[2], P. Sinh[2], M. Dave[2], J. Katz[2], K. Ropella-Panagis[2], V. Gulani[2]; [1]Bern/CH, [2]Cleveland, OH/US, [3]Rochester, MN/US

**Purpose:** MR fingerprinting (MRF) provides quantitative mapping of $T_1$ and $T_2$ relaxation times of a tissue from a single acquisition. The purpose of this study was to evaluate the feasibility and utility of MRF-based relaxometry in the bowel for quantitative evaluation of disease activity in Crohn's disease.
**Material and methods:** 52 patients (27:25 M:F) undergoing MR enterography exams at 1.5T were included in this IRB-approved study. 3 axial and 3 coronal 2D-MRF slices were acquired through the bowel using a single breath-hold MRF-fast imaging with steady-state precession sequence. Regions of interest (ROIs) were drawn in the wall of small bowel and colon to assess $T_1$ and $T_2$ relaxation times of unaffected, active (wall edema, stratified or layered enhancement, adjacent mesenteric edema) and/or chronic inflamed segments (fibrotic wall thickening ± fatty infiltration, fat wrapping), for each patient. Information from weighted clinical images and endoscopy was used to identify affected segments. Mann-Whitney U and Kruskal-Wallis test with Dunn-Bonferroni post hoc tests were used to assess differences in $T_1$ and $T_2$ values between unaffected, acutely, and chronically inflamed bowel.
**Results:** There were 17 segments with chronic disease and 20 with acute inflammation. $T_1$ relaxation times were significantly longer in unaffected segments (1428±327 ms, n=76) versus inflamed segments (active 1309±322 ms and chronic 12138±301 ms), p = 0.004. $T_2$ relaxation times were longer in segments with active inflammation (67±20 ms) versus chronic fibrotic segments (44±19 ms), p = 0.003.
**Conclusion:** Initial application of MRF in bowel imaging is presented, with promising results for quantitative differentiation of unaffected, actively inflamed and chronically diseased bowel.

### SS 11.3

### Evaluation of a new CT enterography score for inflammatory activity in Crohn's disease: correlation with laboratory and endoscopic findings, and inter-reader agreement

A. Agostini, A. Borgheresi, M. Antonarelli, D. Campioni, L. Ottaviani, S. Gemini, A. Pisani, P. Mosca, A. Giovagnoni; Ancona/IT

**Purpose:** To test laboratory and endoscopic correlation, and inter-reader agreement (IRA) of a new semiquantitative score for the evaluation of Crohn's disease (CD) activity at CT enterography (CTE).
**Material and methods:** Inclusion criteria: patients with endoscopic or pathological diagnosis of ileocolic CD who underwent a CTE between June 2017 and August 2018 with endoscopic and laboratory data [fecal calprotectin (FC) and C-reactive protein (CRP)] within 2 weeks. CTE was performed with a LightSpeed VCT (GE Healthcare) after administration of contrast material (Iopamidol 370 mg I/mL, Bracco, Milan). A 5-point score based on CTE findings within intestinal wall and mesentery was set from the literature review (0 = not involved segment; 4 = severe inflammation). Two radiologists with different experience in abdominal imaging reviewed the images, in consensus for clinical correlations. The CTE score was correlated with endoscopic findings (score SES-CD score), CRP and FC with non-parametric tests and ROC curves. IRA was calculated with intraclass correlation coefficient (ICC).
**Results:** 48 patients (28M/20F; median age 49 years) and 63 segments were included. The CTE score stratified CRP and FC values (Kruskal-Wallis p<0.05), with a sensitivity of 93% and a specificity of 56% for endoscopic activity. IRA had ICC of 0.372-0.922 for different findings and score.
**Conclusion:** The CTE score provided a good correlation with laboratory and endoscopic findings, and a variable IRA for the different findings.

### SS 11.4

### Which diffusion-weighted imaging - Short-TI Inversion Recovery or Spectral Presaturation with Inversion Recovery-based - is better for the assessment of quantification of Crohn's disease inflammation?

I. Apine, R. Pitura; Riga/LV

**Purpose:** To assess the relationship between apparent diffusion coefficients (ADC) of free-breathing Short-TI Inversion Recovery (STIR) based diffusion-weighted imaging with background body signal suppression (DWIBS) and magnetic resonance activity index (MaRIA) score, and to compare it with correlation between ADC of the "conventional" respiratory-triggered Spectral Presaturation with Inversion Recovery (SPIR) based DWI and MaRIA score in patients with active Crohn's disease.

**Material and methods:** 10 children and 5 adults with active Crohn's disease of terminal ileum were included in the study. In each patient, the diseased bowel was divided into approximately 5-cm-long segments. The total number of segments in children was 25 and in adult 31. Three measurements of ADC-DWI and ADC-DWIBS of region of interest 2-3 $mm^2$ in the site of highest SI in b=800 images were performed in each of the segments. Three relative contrast enhancement measurements were performed in the same locations of each segment from which MaRIA score was calculated. Correlation between mean values of ADC-DWI, ADC-DWIBS and MaRIA score was assessed in each segment by calculation of Pearson correlation coefficient. p value of <0.05 was chosen as the significance level.

**Results:** There was weak negative correlation between ADC-DWIBS and MaRIA (r=-0.378). The correlation between ADC-DWI and MaRIA was negative moderate (r=-0.505).

**Conclusion:** Correlation between ADC-DWIBS and MaRIA was weaker compared to correlation between ADC-DWI and MaRIA. This suggests that ADC of "conventional" DWI might be superior over ADC of DWIBS in the calculation of DWI-based MR activity index of Crohn's disease.

### SS 11.5

### Structured report on small bowel Crohn's Disease: how we developed it

P. Rudenko[1], A. Batista Domenech[1], A. Perez Girbes[1], P. Stajgis[2], J.P. Azorin Vicente[1], A. Torregrosa Andrés[1], L. Marti- Bonmati[1]; [1]Valencia/ES, [2]Poznan/PL

**Purpose:** To describe the process of elaborating a structured report on Crohn's disease and to propose MR-enterography-based structured report in patients with Crohn's disease.

**Material and methods:** After a comprehensive literature review, a multidisciplinary committee consisting of radiologists, gastroenterologists, surgeons and pathologists, all of them specialists in Inflammatory bowel disease (IBD), was assembled. After several weeks of discussion, a consensus was reached on 43 items to be described. The biomedical engineers from our research group developed a web-based platform using Angular* with Mango Database. All data were stored in our server.

**Results:** The structured report was based on HTML format and includes mainly customized checkbox for each item, most of them being dropdown lists. The following sections were included: 1. clinical data (purpose of exam, Harvey-Bradshaw Activity Index, laboratory results, Montreal classification, current treatment state, year of established diagnosis, previous surgical procedures). 2. Technical MRI aspects (MR field, UKCA applied and dose, previous oral contrast and patient preparation, quality of the study). 3. Lesion description, subclassified as 3A) main lesion considered as the longest one (localization, length, maximum wall thickness, edema, ulceration, hyperenhancement, fibro-fatty proliferation, fat stranding, lymph nodes, free fluid, magnetic resonance index of activity (MaRIA) score, stenosis, enhancement curve and extraluminal complications; 3B) other complicated lesions (stenosis or extraluminal complications); and C) secondary lesions. 4. Incidental findings.

**Conclusion:** The proposed structured report for Crohn's disease aims standardisation, comparability and completeness in the quality and accessibility of the radiologic information, providing integration and combination of radiology data elements with other key clinical parameters in a structured database for further exploitation.

### SS 11.6

### MR enterography for the assessment of postoperative GI function: technique and patient tolerability

S.J. Chapman[1], J.A. Helliwell[1], A. Menys[2], D.G. Jayne[1], D. Tolan[1]; [1]Leeds/UK, [2]London/UK

**Purpose:** Ileus is common after colorectal surgery, but its diagnosis is an unmet clinical challenge. Traditional measures of gut function (such as passage of flatus and stool) are limited by patient and assessor bias. We explored the feasibility of magnetic resonance enterography (MRE) performed three days after surgery for the assessment of GI function.

**Material and methods:** A technical description of the MRE protocol and patient tolerability are described in a study population of patients undergoing laparoscopic colorectal resection and receiving MRE within an ongoing, double-blinded, randomised controlled trial. The MRE protocol was devised and iteratively refined prior to study initiation by a multi-disciplinary group of GI radiologists, radiographers, colorectal surgeons, and patient representatives.

**Results:** MRE was performed on the third postoperative day in 18 patients. An enteral challenge of 2 glasses of water was administered 30 minutes prior to the scan. A series of 60-second, coronal, balanced gradient-echo motility scans were performed (20-second breath-hold and 40-second free-breathing). Scan parameters included 2cm slice gap, 1cm slice thickness, and 1 image-per-second temporal resolution (total scan time 20-30 minutes). Global small bowel motility was quantified using dedicated software. Seventeen (n=17/18; 94.4%) patients completed the MRE protocol. One patient terminated the scan early because of abdominal discomfort. There were no adverse events attributed to the MRE protocol.

**Conclusion:** A comprehensive MRE protocol assessing postoperative GI function was feasible and well tolerated on postoperative day 3 after laparoscopic colorectal resection. Further evaluation is required to determine the clinical applicability of this form of assessment.

### SS 11.7

### Qualitative and quantitative analyses of virtual non-contrast images in CT enterography with a 3rd-generation dual-source dual-energy CT

A. Agostini, A. Borgheresi, M. Antonarelli, F. Cela, L. Ottaviani, F. Terilli, A. Giovagnoni; Ancona/IT

**Purpose:** To assess image quality and differences in attenuation values of virtual non-contrast (VNC) images of CT enterography (CTE) in Crohn's disease (CD) with 3rd-generation dual-source dual-energy CT (dsDECT).

**Material and methods:** Inclusion criteria: CTE performed with Somatom Force (Siemens Healthineers) in patients >18 years old with known CD between February and December 2018. Scan protocol: pre-contrast at 120 kV, enterographic phase at 100/Sn150 kV after contrast material (Iopamidol 370 mg I/mL; Bracco) and water solution of polietilenglicole (MacroP, Sigmar), modulated mA, slice thickness 2 mm. VNC images were obtained with liver VNC (Syngo.via Frontier, Siemens Healthineers). Attenuation values in VNC and TNC were measured with regions of interest on bowel wall, lumen, and perivisceral fat. Image quality was evaluated on a 5-point scale; signal- and contrast-to-noise ratios (SNR and CNR) were calculated. Non-parametric tests were used and differences in attenuations were calculated with Bland-Altman test.

**Results:** 31 CT examinations were included. The qualitative analysis reported a median score of 4.3/5 for TNC and 3.4/5 for VNC (Friedman p=0.038). SNR and CNR were significantly higher in TNC (Wilcoxon p<0.05). Higher and significant differences in densities (>5HU) were recorded for jejunal and jejuno-ileal wall, in jejunal, jejuno-ileal lumen, and in perivisceral fat of ileal and involved segment and in Wilcoxon p<0.05.

**Conclusion:** CTE studies with 3rd-generation dsDECT provided VNC images with acceptable quality and variable differences in HU.

### SS 11.8

### MR fistulography with percutaneous instillation of US gel and its role in preoperative mapping

G. Fontanella, M. Mancinelli, A. Festa, S. De Lucia, C.A.T. Manganiello, R. Villanacci; Benevento/IT

**Purpose:** The aim of this study is to show the role of MR fistulography (MRF), performed after percutaneous instillation of US gel, in the preoperative mapping of perianal fistula, underlying its various advantages.
**Material and methods:** 68 patients were selected from September 2017 until September 2018 for pre-operative fistular mapping among those with viable percutaneous orifices. After a basal scan (22 min), we instilled 3-10 cc of US gel per orifice, then re-scanned using a T2w, fsT2w and DWI protocol. Each case was reviewed through standard reporting by experienced radiologists, comparing either standard MR or MRF reports with the surgical findings. Surgeons in our institution are currently operating on the basis of MRF reports.
**Results:** MRF has shown to be a well-tolerated, faster technique (mean scan time 16 mins), superior to standard MR, allowing the detection of 94 tracts (versus 82 of basal MR). MRF is highly congruous with surgical findings, with specificity and sensitivity in the definition of fistular grade close to 100%, thus resulting in a significant drop of fistular relapses in one year: 2.89% vs 22.43% (from Sept 2016 to Sept 2017). Specificity, sensitivity and accuracy of detection of internal openings range between 90 and 97.5%, data comparable to basal MR.
**Conclusion:** MRF with percutaneous instillation of US gel is a more accurate, well-tolerated, faster technique for pre-operative mapping, allowing better depiction of lesser tracts and, in our institution, significant drop in fistular relapses.

### SS 11.9

### Cine-MRI for the quantification of antral motility: comparison between obese and normal weight people

S. Picchia[1], M. Rengo[1], M.A. Bali[2], D. Bellini[1], S. Badia[1], I. Carbone[1], A. Laghi[3]; [1]Latina/IT, [2]Brussels/BE, [3]Rome/IT

**Purpose:** To quantitatively compare the gastric motility between obese and normal weight people with cine-MRI.
**Material and methods:** In this non-randomized prospective single-center study, obese patients candidated for sleeve gastrectomy (OB) and normal weight volunteers (NW) were included. Cine-MRI (1.5T) was performed using 2D-TRUEfisp on a plane parallel to the long axis of the antrum. Images were acquired before (T0), immediately after the end of a liquid meal intake (T1) and every 20 minutes (T2-T5) for a total exam time of 100 minutes. Each sequence lasted 60 seconds, with 120 images acquired. Two radiologists evaluated images on a dedicated software. Antral diameters (AD) and antral contraction waves' width (ACWw) and amplitude (ACWa) were assessed on each image of the entire sequence. The results obtained in OB and NW were compared, using a non-parametric test. The inter-reader and intra-reader agreement were evaluated.
**Results:** Our final population consisted of 50 patients, 25 for each group. Cine-MRI showed that, both during fasting and postprandial period, in OB all parameters (AD/ACWs width/amplitude) were significantly lower (p value=0.002/0.003/0.001, respectively). A good inter-reader and a very good intra-reader agreement were observed.
**Conclusion:** The cine-MRI showed a significantly more frequent altered motility in OB compared to NW. The obesity-related cause of the impaired antral motility, whatever it is, could become a new potential therapeutic target.

### SS 11.10

### Is there a relationship between the number of middle colic artery and transverse colon length? A study of radiological anatomy with 3D CT

S. Pashapoor; Bursa/TR

**Purpose:** Preoperative knowledge of the normal pattern and variations of the mesenteric arteries is substantially important in the successful surgery. In this study, we identified the relationship between middle colic artery (MCA) variations and the transverse colon length using 3D multidetector CT.
**Material and methods:** We included 293 randomizing patients in the study who underwent abdominal multidetector CT for various clinical reasons in Ankara university hospital between Jul 2014 and May 2016.
**Results:** From the total 293 patients, 168 (57.3%) were male and 125 female (42.7%) and the median age was 53. The median length of transverse colon was measured to be 439 mm in the whole patients. There were 80 patients with double and 213 patients with one MCA. The median length of transverse colon in patients with one MCA was 425 mm where it was 465 mm in those with double MCAs ($p<0.001$).
**Conclusion:** Preoperative 3D-CT is informative and very helpful for surgeons in colonic resections. In this study, the transverse colon was longest in patients with double MCA in comparison with the patients with one MCA.

**11:00 - 12:30** **Room Pisa**

*Scientific Session SS 12*

### Advanced MRI in liver imaging

### *SS 12.1*

### Agreement between MR elastography liver stiffness estimates obtained from fully automated convolutional neural network-based and manually drawn regions-of-interest

T.I. Delgado[1], G. Moura Cunha[1], K.A. Hasenstab[1], D.N. Batakis[1], K. Wang[1], A. Mamidiapalli[1], A.S. Boehringer[1], W. Henderson[1], C. Chung[2], S. Djedjos[2], R.P. Myers[2], M. Middleton[1], C.B. Sirlin[1]; [1]San Diego, CA/US, [2]Foster City, CA/US

**Purpose:** Current clinical liver stiffness estimation from magnetic resonance elastography (MRE) requires manual placement of regions-of-interest (ROIs). Manual ROI placement is laborious and subjective, introducing variability within and between readers. We developed a fully automated convolutional neural network (CNN)-based method to generate ROIs on liver MRE images, and examined agreement in liver stiffness estimation between automated and manual ROI placement.
**Material and methods:** This was a cross-sectional, secondary analysis of a multi-site cohort of 138 adults with nonalcoholic steatohepatitis who underwent 2D liver MRE (1.5T Siemens n=52, 3T Siemens n=81, 1.5T GE n=4, 3T Philips n=1) in two drug development clinical trials (NCT02854605 and NCT02466516). An automated algorithm generated MRE ROIs in two steps: first, a CNN segmented the liver on MRE magnitude images; second, pixels within one centimeter of liver edges or with unreliable stiffness values (goodness-of-fit < 0.95) were excluded. Independently, image analysts manually placed ROIs using standardized procedures. Agreement between stiffness values derived from automated versus manually generated ROIs was assessed with intraclass-correlation and Bland-Altman analyses.
**Results:** Automated and manually estimated liver stiffness means, standard deviations, and ranges were 3.9 kPa; 1.5 kPa; 2.0-8.7 kPa and 3.8 kPa; 1.7 kPa; 1.9-9.6 kPa, respectively. Inter-method intraclass correlation was 0.949 (95% confidence interval: 0.930, 0.963). Bland-Altman bias was -0.11 kPa (95% limits of agreement: -1.10, 0.88 kPa).
**Conclusion:** Liver stiffness estimation using fully automated CNN-based MRE ROI placement agrees with values obtained using manual ROI placement. With further development, such a method could be used to help automatically analyze MRE in clinical trials and in clinical care.

### *SS 12.2*

### Enhancement patterns and immunohistochemical features of gadoxetic acid-enhanced MRI in patients with non-alcoholic fatty liver disease: new results

D. Feier[1], N. Bastati-Huber[2], A. Beer[2], S. Pötter-Lang[2], R. Fragner[2], A. Ba-Ssalamah[2]; [1]Cluj-Napoca/RO, [2]Vienna/AT

**Purpose:** To quantify enhancement features of gadoxetic acid-enhanced MRI in patients with non-alcoholic fatty liver disease (NAFLD) and correlate them with the immunohistochemical expression of the hepatocyte transporters (OATPB2/8, MRP2, MRP3) and blood biomarkers.
**Material and methods:** The local institutional review committee approved this study and waived written informed consent. This was a retrospective study of gadoxetic acid-enhanced 3T MRI performed in 46 consecutive patients with NAFLD (mean age (SD), 49.46 (15.52) years). The MR images were analyzed using the relative enhancement (RLE). Univariate and multiple regression analyses were applied to identify variables associated with OATPB2/8, MRP2, MRP3 expression.
**Results:** A statistically significantly higher number of patients with NASH had increased lobular inflammation (p<0.0001), ballooning (p<0.0001), liver steatosis (p=0.003) and liver fibrosis (p=0.002) compared to those with simple steatosis. RLE, NAFLD fibrosis score and FIB-4 were independent predictors of NASH patients. Together they were able to differentiate NASH from simple steatosis patients with a probability of 87% [AUROC=0.87 (95%CI 0.74-0.94)], a sensitivity of 85% (95%CI 70.2-94.3), and a specificity of 75% (95%CI 42.8-94.2). Relative enhancement was positively correlated with the expression of OATP2/8 (r=0.41, p=0.002) and blood biomarkers positively correlated with the expression of MRP2 (r=-0.37, p=0.007).
**Conclusion:** Imaging parameters derived from the hepatobiliary phase of gadoxetic acid-enhanced MRI enable us to discriminate between patients with NASH and simple steatosis and correlated well with blood biomarkers and immunohistochemical results.

### *SS 12.3*

### Comparison of diagnostic performance of 'short' and 'long' MRI protocols for the detection of colorectal liver metastases

C. Ghorra[1], R. Pommier[1], S. Terraz[2], V. Vilgrain[1], M. Ronot[1]; [1]Clichy/FR, [2]Geneva/CH

**Purpose:** To compare the diagnostic performance of 'short' and 'long' MRI protocols for the detection of colorectal liver metastases.
**Material and methods:** From 2006 to 2015, 67 patients (44 men (66%) and 23 women (34%), mean 65±11 y-o) undergoing liver gadoxetic acid-enhanced MRI during the initial workup of colorectal cancer were retrospectively included. MR images were independently reviewed by two readers blinded to clinical data in 2 separated reading sessions: 1) including all acquired sequences ('long' protocol) and 2) including only T2-w, diffusion-w and hepatobiliary phase images ('short' protocol). For each reading, readers were asked to characterize all detected lesions using a 5-point scale (1: certainly benign to 5: certainly malignant). A lesion was considered a metastasis when score $^{3}$3. Standard of reference was pathology (when resected) or >12 months follow-up. Chi$^{2}$ and Student's t test were used.
**Results:** A total of 486 lesions including 331 metastases (68%) were analyzed. The metastasis detection rate was 86.1% (IC95[82-89.4])–86.7% (IC95[82.6-90]) and 85.8% (IC95[81.6-89.2])–87% (IC95[82.9-90.2]) with the 'short' and 'long' protocols, respectively (p>0.99). Among detected lesions, 92.1% (IC95 [89.1-94.4])–94.8% (IC95 [92.2-96.6]) and 84.6% (IC95 [80.8-87.7])–88.8% (IC95 [85.4-91.5]) of lesions were correctly classified with the 'short' and 'long' protocols, respectively (p=0.13 and p=0.10). Results remained unchanged when only score$^{3}$4 was used for the diagnosis of CLM.
**Conclusion:** A 'short' gadoxetic-enhanced MR protocol including T2-w, diffusion-w and hepatobiliary phase shows similar diagnostic performances to that of a 'long' protocol including dynamic phase. It may be useful to shorten exam duration and facilitate patient's workup.

### SS 12.4

### Accuracy of gadolinium ethoxybenzyl diethylenetriamine pentaacetic acid-enhanced MRI for the diagnosis of liver metastases from neuroendocrine tumors

R.H. Hayoz, C. Dromain, N. Vietti Violi, J.-F. Knebel, R. Duran; Lausanne/CH

**Purpose:** To compare the diagnostic accuracy of dynamic phases, hepatobiliary phase (HBP), after gadolinium ethoxybenzyl diethylenetriamine pentaacetic acid (Gd-EOB-DTPA)-enhanced MRI and diffusion-weighted imaging (DWI) for the detection of liver metastases from neuroendocrine tumors.
**Material and methods:** After obtaining approval from the ethical committee of research, 67 patients (mean age: 60 years, range: 18-84 years) were included in this retrospective study. All patients underwent MRI with injection of Gd-EOB-DTPA for suspected liver metastases. Four imaging sets were compared (DWI, HBP, combined DWI+HBP and T2W MRI) by three experienced radiologists with different levels of experience. Lesions were evaluated on a 3-point scale. Sensitivity and specificity were calculated for each imaging set. The gold standard was based on histopathological results when available or the interpretation of previous follow-up imaging. Interreader agreement was evaluated by interclass correlation (ICC). Univariate logistic regression was performed to evaluate lesion criteria such as ADC value, lesion size and enhancing pattern on dynamic sequences.
**Results:** 618 lesions (545 metastases, 73 benign lesions) were identified. The combined reading of DWI and Gd-EOB-DTPA-enhanced MRI led to a significantly higher detection rate of lesion compared to the three other imaging sets (sensibility: 86%, specificity: 94%). Interreader agreement was highest in HBP enhanced (0.96 (0.94-0.97)(CI: 95%), followed by DWI and HBP. High ADC, increasing lesion size and hypervascular enhancing pattern lower the risk of misinterpretation of malignant lesion.
**Conclusion:** Associating DWI with HBP after Gd-EOB-DTPA-enhanced MRI increases diagnostic accuracy in patients with neuroendocrine liver metastases suggesting the potential use of fast MR protocol with pre-injection of Gd-EOB-DTPA.

### SS 12.5

### Iso- or hyperintensity of hepatocellular adenomas on hepatobiliary phase does not always correspond to hepatospecific contrast agent uptake

E. Reizine[1], M. Ronot[2], F. Pigneur[1], Y. Purcell[2], S. Mule[1], M. Dioguardi Burgio[2], V. Vilgrain[2], A. Luciani[1]; [1]Créteil/FR, [2]Clichy/FR

**Purpose:** To evaluate if iso- or hyperintensity of hepatocellular adenomas (HCAs) on hepatobiliary phase (HBP) is systematically related to high uptake of hepatospecific contrast agent, using a quantitative approach.
**Material and methods:** This bi-centric retrospective study included all patients with histologically confirmed and subtyped HCA from 2009 to 2017 who underwent MRI with HBP after gadobenate disodium (Gd-BOPTA) injection and who showed iso- or hyperintensity on HBP. The signal intensity of tumors on pre- and post-contrast images, and the presence of hepatic steatosis were noted. Contrast uptake on HBP was quantified using the liver-to-lesion-contrast-enhancement ratio (LLCER).
**Results:** 24 HCA showed iso- or hyperintensity on HBP, specifically 17 inflammatory (IHCA) and 7 ß-catenin HCAs (BHCA). Eighteen HCAs (75%) [17 IHCA and 1 BHCA] had a LLCER<0% (median -13.6% %, group 1), of which 94% were hyperintense on pre-contrast T1-w images, with background hepatic steatosis. Six HCAs (25%) had LLCER≥0% (median 2.9%, group 2), all were BHCA. A LLCER ≥1.6% was associated with the diagnosis of BHCA with a sensitivity of 86% and a specificity of 100%.
**Conclusion:** Iso- or hyperintensity of HCAs on HBP does not necessarily correspond to a higher hepatospecific contrast agent uptake when compared to that of the surrounding liver. In inflammatory HCA, spontaneous tumor hyperintensity on pre-contrast images and underlying steatosis likely explain the iso- or hyperintensity of these tumors, which do show reduced HBP contrast agent uptake. On the other hand, marked contrast uptake can be observed, especially in BHCA. This emphasizes the importance of quantitative analysis of contrast uptake for tumor subtyping.

### SS 12.6

### Hepatic localization of extramedullary hematopoiesis in beta-thalassemia patients: diagnostic accuracy of T2*, apparent diffusion coefficient and gadolinium-enhanced dynamic MRI

P.P. Arcuri[1], A.K. Sikora[1], S. Roccia[1], V. Aiello[1], G. Fodero[1], C. Bertucci[2], M.C. Galati[1], D. Laganà[1]; [1]Catanzaro/IT, [2]Buckingham/UK

**Purpose:** The aim of our study was to evaluate the diagnostic significance of T2*, apparent diffusion coefficient (ADC) values and gadolinium-enhanced dynamic sequences, in the MRI study of the hepatic masses of extramedullary hematopoiesis (EMH), expressed in terms of sensitivity, specificity and diagnostic accuracy.
**Material and methods:** We retrospectively evaluated, using MRI with T2* sequence, ADC and gadolinium-enhanced dynamic sequences, thirty-eight hepatic heteroplastic lesions, found in fifteen patients, shown at biopsy, EMH localizations in ß-thalassemia patients. We calculated, by ROI, T2*lesion/liver values, ADC lesion/liver values and contrast enhancement features. Data were expressed in terms of median value ± range. Kruskal-Wallis non-parametric test was performed. Sensitivity, specificity and accuracy were assessed. Fisher's exact test was used to evaluate statistical significance. A p value<0.05 was considered statistically significant. The biopsy of the masses was the gold standard reference.
**Results:** The EMH hepatic lesions showed median T2* lesion/liver =0.92 (values between 0.78 and 1.02), ADC lesion/liver median value=1.02 (values between 0.64 and 1.25). T2* lesion/liver showed sensitivity=93%, specificity=97%, accuracy=94%. ADC lesion/liver showed sensitivity=81%, specificity=73%, accuracy=71%. After the administration of 0.5 mmol/kg gadolinium (Gd-BOPTA), heterogeneous mild enhancement was detectable in the arterial phase (87%), homogeneous and intense enhancement on the arterial phase (13%). Enhanced persistently until five minutes after Gd administration (81%), the lesions were washed out to hypointensity in the portal venous and 5-minute delayed phases (19%). Gadolinium-enhanced dynamic sequences showed sensitivity=83%, specificity=81% and accuracy=87%.
**Conclusion:** In the MRI diagnosis of hepatic mass of EMH, highest accuracy diagnosis was obtained by T2*mass/liver (94%, p=0.001) versus 83% of gadolinium-enhanced dynamic sequences and 71% of ADC mass/liver. It is, therefore, appropriate to include these sequences in the MRI study protocol in ß-thalassemic patients with suspicion of EMH hepatic lesions.

### SS 12.7

### Hepatocellular adenomas: is there additional value of using gadolinium-ethoxybenzyl-diethylenetriamine penta-acetic acid in subtype differentiation?

U. Fehrenbach[1], T.A. Auer[1], C. Grieser[1], N. Raschzok[1], H. Bläker[1], D. Geisel[1], D. Seehofer[2], T. Müller[1], M. Schmelzle[1], T. Denecke[1]; [1]Berlin/DE, [2]Leipzig/DE

**Purpose:** Morphologic and quantitative characteristics to differentiate among hepatocellular adenoma (HCA) subtypes evaluated with gadolinium-ethoxybenzyl-diethylenetriamine penta-acetic acid (Gd-EOB-DTPA) enhanced MRI.
**Material and methods:** Fifty-six patients with 77 histopathologically proven HCA examined with gadoxetic acid-enhanced MRI were retrospectively enrolled (standard of reference: surgical resection, n=71; biopsy, n=6). Three radiologists evaluated all MR images regarding morphological features in consensus and hepatocyte-specific enhancement was quantified. Histopathological subgroup analysis was based on the Bordeaux Classification (including steatotic (H-HCA), inflammatory (I-HCA), b-catenin (b-HCA) and unclassified (U-HCA) adenomas).
**Results:** Overall, 22 H-HCA (29%), 32 I-HCA (41%), 5 b-HCA (6%) and 18 U-HCA (24%) were present. For differentiation of HCA subtypes, presence of intralesional fat (H-HCA, n=21; I-HCA, n=4, b-HCA, n=1; U-HCA, n=2), atoll sign (I-HCA, n=19) and a central scar (b-HCA) were significant (p<0.001). For hepatobiliary phase, most HCA were visually found being hypointense (overall: 71%; H-HCA = 96.5%; I-HCA = 53.5%; b-HCA = 80%, U-HCA = 66.6%). I-HCA showed higher intensities in HBP than the other subtypes, but the findings were not statistically significant.
**Conclusion:** Following the Bordeaux classification, typical morphologic MR appearances of the different HCA subtypes is present and reliable. On hepatobiliary phase, most HCA show hypointensity; however, iso- to hyperintensity may be present in all HCA subgroups while I-HCA seems to be most heterogenous. Combining Gd-EOB-DTPA behavior, the qualitative MR characteristics and predisposing risk factors are recommendable for a more accurate diagnosis.

### SS 12.8

### The effect of steatosis, iron overload, and renal function on the uptake and excretion of gadoxetic acid-enhanced MRI

S. Pötter-Lang, L. Beer, N. Bastati-Huber, M. Mandorfer, M.C. Elmer, T. Reiberger, A. Ba-Ssalamah; Vienna/AT

**Purpose:** To explore the effect of steatosis, hepatic iron overload, and renal function on gadoxetic-acid (GA) uptake in hepatobiliary phase (HBP) images in patients with diffuse chronic liver disease (CLD).
**Material and methods:** We included 265 patients (mean age, 54 ±16 years) with CLD who had undergone GA-enhanced MRI of the liver. Demographic and clinical data were obtained from our patients' medical history and included age, sex, body mass index, creatinine, and estimated glomerular filtration rate (eGFR). Relative liver enhancement (RLE) was calculated by two radiologists using unenhanced and GA-enhanced HPB images, 20 minutes after the administration of GA. Hepatic fat fraction (%) was measured on dual-echo, gradient-recalled echo sequences. Hepatic siderosis was semiquantitatively assessed on T2-weighted, fat-suppressed images (Grade 1-3). Spearman´s correlation coefficients were used to assess the correlation between RLE and laboratory parameters. ANOVA with a Bonferroni post hoc test was used to compare different groups.
**Results:** There was a significant correlation between RLE and eGFR (R=0.225, p<0.001) and siderosis grade (R=-0.210, p=0.001), while hepatic fat fraction did not correlate with RLE (R=0.098, P=0.116). RLE in patients with highly impaired renal function (eGFR<30 ml/min/1.73 m²) was significantly lower than in patients with slightly impaired (eGFR 30-90) or normal kidney function (eGFR >90) (RLE: 46% vs. 82% vs. 89%, p<0.001). In addition, RLE was significantly lower in patients with severe siderosis compared to those with no siderosis (RLE: 46% vs. 86%; p=0.011).
**Conclusion:** RLE significantly correlates with renal function; however, it is independent of the hepatic fat fraction in patients with diffuse CLD.

### SS 12.9

### Findings of sinusoidal obstruction syndrome on gadoxetic acid-enhanced MRI in patients with chemotherapy for colorectal liver metastases are poorly reproducible between radiologists

F. Staal[1], J.B. Houwers[2], B. Heeres[1], D. Van Dorth[1], D. Lambregts[1], R.G.H. Beets-Tan[1], M. Maas[1]; [1]Amsterdam/NL, [2]Maastricht/NL

**Purpose:** Neoadjuvant chemotherapy in patients with colorectal liver metastases (CRLM) may cause sinusoidal obstruction syndrome (SOS), potentially leading to increased morbidity after resection and decreased chemotherapy effect. Therefore, it is important to identify SOS. Studies have suggested to use gadoxetic acid-enhanced MRI (EOB-MRI) for the diagnosis of SOS. The purpose of our study was to assess the reproducibility of EOB-MRI to determine the presence and severity of SOS in patients treated with chemotherapy for CRLM.
**Material and methods:** 32 patients treated with chemotherapy for CRLM (either oxaliplatin-based, oxaliplatin-based+bevacizumab, or non-oxaliplatin-based treatment), with available EOB-MRI scans in the hepatobiliary phase, were retrospectively included. The presence and severity of SOS were independently scored by three radiologists with varying experience (10-20 years of experience in EOB-MRI), who were blinded to the clinical data, using a 5-point scale (SOS score: 0=definitely not present to 4=definitely present). The interobserver agreement between readers was assessed with quadratic weighted kappa statistics.
**Results:** The mean time between chemotherapy completion and EOB-MRI scan was 1.1 (+/-1.4) months. Interobserver agreement was poor, with kappas ranging from 0.25 (95%CI 0.01-0.48) to 0.33 (95%CI 0.08-0.58). Furthermore, analysis of the SOS scores revealed that less experienced radiologists tended to score more equivocal scores (6.3% and 43.8% SOS score = 2), compared to a more experienced radiologist (3.1% SOS score=2).
**Conclusion:** The assessment of SOS on hepatobiliary phase EOB-MRI in patients treated with chemotherapy for CRLM shows a poor inter-observer agreement for the diagnosis of SOS. This questions the clinical value of hepatobiliary phase EOB-MRI to detect SOS.

### SS 12.10

### Inter-observer reproducibility of liver stiffness measurement using MR elastography

V. Phou, P.-A. Lampson, A. Fourquet, M. Dubois, P.-H. Jouve De Guibert, A. Ayed, A. Prenois, L. Le Deunf, J. Barcellitti, O. Lucidarme, M. Wagner; Paris/FR

**Purpose:** To assess the inter-observer reproducibility of liver stiffness measurement using magnetic resonance elastography (MRE) in a large panel of observers, including technicians.
**Material and methods:** Fifty-five consecutive patients with liver MRE acquisition and pathological analysis of the liver parenchyma were included in this retrospective study (F0-2: N=26/F3-F4: N=29). MRE was acquired on a 1.5T Siemens system, using a gradient-recalled echo (GRE) MRE sequence. Eight observers (5 physicians and 3 technicians) were first asked to draw in the liver 2 elliptical regions of interest (ROI) (250-500mm²) and then one free-hand ROI, as large as possible based on the confidence map and the anatomy. Inter-observer reproducibility was assessed using the intra-class correlation coefficient (ICC) and Bland-Altman analysis. Inter-observer reproducibility of the MRE-METAVIR classification (threshold [F0-F2] vs [F3-F4]=4kPa according to Dyvorne et al.) was assessed using the Cohen's kappa coefficient.
**Results:** Inter-observer reproducibilities of 2 types of ROI were excellent with ICC>0.98, a mean bias equal to 0.11 kPa ([0.002-0.2]/[0.004-0.34]), and limits of agreement at 1.76 [1.21-2.85] for elliptical ROIs and 1.47 [0.78-2.22] for the free-hand ROI. The bias was lower in the sub-group of physicians (0.06/0.09 kPa). The MRE-METAVIR classification had a sensitivity and a specificity of 55.2-65.5% and 80.8-92.3%. Its reproducibility was substantial-excellent (K>0.620/P<0.0001) (except for one pair of readers) and tended to be better between physicians (K>0.733/P<0.0001).
**Conclusion:** Inter-observer reproducibility of liver stiffness measurement using MRE is substantial to excellent, even when performed by technicians.

11:00 - 12:30 Room Bologna

*Scientific Session SS 13*

### Diffuse liver diseases: fibrosis, cirrhosis and portal hypertension

### SS 13.1

### Performance of morphologic criteria for the diagnosis of cirrhosis in patients with different etiologies of chronic liver disease

R. Cannella[1], N. Dasyman[2], A. Furlan[2], A.A. Borhani[2]; [1]Palermo/IT, [2]Pittsburgh, PA/US

**Purpose:** To investigate the sensitivity of morphologic criteria for the detection of cirrhosis in alcoholic liver disease (ALD), hepatitis C (HCV), and non-alcoholic steatohepatitis (NASH).

**Material and methods:** This is a retrospective study of 100 patients (53M, 48F) with different etiologies of chronic liver disease (CLD) including NASH (n=41), HCV (n=39), and ALD (n=20) with different degrees of fibrosis on liver biopsy. Two readers (R1:3 years' experience in advanced liver imaging; R2: junior radiology resident) independently analyzed the CT exams performed within 6 months of biopsy for the presence of morphologic changes of cirrhosis (surface nodularity, fissural/periportal widening, increased caudate-right lobe ratio) and portal hypertension (splenomegaly, varices, ascites). Each reader assigned an overall score for cirrhosis and portal hypertension. Sensitivity/specificity of morphologic criteria in each etiology group was calculated for each reader and compared using Chi-squared test. Frequencies of different morphological changes in each group were compared.

**Results:** Using morphologic criteria, sensitivity for the diagnosis of cirrhosis was significantly lower in NASH (R1: 81%; R2: 63%) compared to ALD (R1: 95%; R2: 90%) and HCV (R1: 85%; R2: 77%) for both readers (p<0.001). The reader with advanced training outperformed the other reader in NASH (p<0.001). Surface nodularity was less common in NASH cirrhosis (p<0.001). Sensitivity for the diagnosis of NASH cirrhosis significantly decreased when only patients without portal hypertension were included (58%, p=0.003).

**Conclusion:** Use of morphologic criteria for the diagnosis of cirrhosis had significantly lower performance in patients with NASH. Liver morphology changes in NASH cirrhosis are more subtle and degree of fibrosis could be underestimated when traditional morphologic criteria are used.

### SS 13.2

### Performance of liver surface nodularity quantification for the detection of portal hypertension in cirrhotic patients: comparison between gadobenate disodium-MRI and CT

N. De Vos, R. Sartoris, F. Cauchy, P.-E. Rautou, V. Vilgrain, M. Ronot; Clichy/FR

**Purpose:** To assess and compare the performance of liver surface nodularity (LSN) quantification on gadobenate disodium (Gd-BOPTA)-enhanced MRI and contrast-enhanced CT for the detection of clinically significant portal hypertension (CSPH) in cirrhotic patients.

**Material and methods:** This retrospective study included patients with compensated cirrhosis who underwent hepatic venous pressure gradient (HVPG) measurement, contrast-enhanced CT and Gd-BOPTA-MRI within 30 days during pre-surgery workup for HCC between January 2015 and August 2018. LSN score was derived either from CT (portal phase) or MRI (T2-w, T1-w portal phase and hepatobiliary phase [HBP]). Accuracy for the detection CSPH was evaluated for each image set with ROC curve analysis. Reproducibility was assessed with intraclass correlation coefficient (ICC) and coefficients of variability (CV).

**Results:** 30 patients were analyzed (23 men [77%], mean 60±11 y-o), including 13 (43%) with CSPH. MRI and CT-derived LSN quantification correlated with HVPG (T2-w: r=0.51, p=0.007, AUROC 0.76±0.10; T1-w portal phase: r=0.43, p=0.028, AUROC 0.78±0.10; HBP: r=0.50, p=0.012, AUROC 0.84±0.09; CT portal phase: r=0.63, p=0.001, AUROC 0.88±0.07). AUROC pairwise comparisons showed no significant difference (p=0.06-0.82). LSN derived from CT, HBP, T1-w portal and T2-w correctly classified 85%, 83%, 81%, and 73% of the patients. Inter-image set comparisons showed excellent reproducibility (ICC from 0.84 to 0.96 and CV from 8.3% to 14.2%).

**Conclusion:** LSN quantification-derived Gd-BOPTA-MRI showed comparable diagnostic performance than LSN derived from portal phase CT for the detection of CSPH in cirrhotic patients.

### SS 13.3

### Liver vein to cava attenuation improves prediction of clinically significant fibrosis when combined with liver volumetry and caudate-right lobe ratio on portal venous CT scans

C. Marx, V. Obmann, J. Hrycyk, L. Ebner, A. Berzigotti, J. Heverhagen, A. Christe, A. Huber; Bern/CH

**Purpose:** This study hypothesized that the liver vein to cava attenuation (LVCA) on portal venous abdominal CT scans is a helpful add-on to liver volumetry and the caudate-right lobe ratio (crl-r) to detect clinically significant liver fibrosis.

**Material and methods:** Fifty consecutive patients with portal venous phase abdominal CT scans and gradient-echo-based MR elastography within 3 months without portal vein thrombosis or prior liver surgery were included. One patient was excluded because of insufficient MR elastography quality. Thirty-six patients had a liver stiffness ≤3.5kPa, while twelve patients had a stiffness >3.5kPa, consistent with clinically significant liver fibrosis (corresponding to a fibrosis stage ≥f2). Liver segmental volume ratio (LSVR), defined as Couinaud segments I-III to segments IV to VIII, as well as LVCA (1-3: liver vein attenuation higher, equal and lower than vena cava, 4: liver veins not contrasted) were calculated. LSVR-A was defined as LSVR*LVCA, while LIMA-FS (liver imaging morphology and attenuation-based fibrosis score) was defined as crl-r*LVCA.

**Results:** In accordance with earlier publications, LSVR correlated well with MR elastography measurements (r=0.63, p<0.001). However, LSVR-A showed an even better correlation (r=0.71, p<0.001). Caudate-right lobe ratio was not very useful (r=0.18, p=0.214), while LIMA-FS was just slightly inferior than volumetry (r=0.52, p<0.001).

**Conclusion:** LVCA is a helpful add-on to volumetry on portal venous abdominal CT scans. LIMA-FS, a combination of LVCA and crl-r, allows a just slightly inferior prediction of clinically significant liver fibrosis than volumetry without time-consuming image post-processing.

### *SS 13.4*

### Evaluation of the risk of esophageal varices bleeding in cirrhotic patients with multi-slice CT portography

S. Wan, B. Song; Chengdu/CN

**Purpose:** To investigate the value of multi-slice CT portography (MSCTP) in predicting cirrhotic esophageal varices bleeding (EVB) and to find new indicators for EVB prediction on MSCTP.
**Material and methods:** A total of 149 patients with esophageal varices (EV) caused by liver cirrhosis were enrolled in this study in the last 2 years of our hospital, all patients underwent MSCTP and endoscopy within 4 weeks, the patients were divided into 68 hemorrhage group and 81 non-hemorrhage group according to whether they were bleeding evaluated by clinical manifestation and endoscopy. Test MSCTP left gastric vein (LGV) diameter, esophageal varices (EV) diameter and the vascular area, calculating sensitivity, specificity, ROC and AUC. All patients were followed up for 6 months, 30 patients were re-admitted due to EVB, and the predictive value of new indicators was verified on their last MSCT appearance.
**Results:** The diameter of LGV, EV and the vascular area of the bleeding group were, respectively (7.81±6.72)mm, (6.78±2.90)mm, (1.72±1.30)$cm^2$, and the non-bleeding group were (6.22±4.61)mm, (5.92±3.05)mm, (1.05±1.26)$cm^2$, the critical points were 5.90mm, 5.15mm, 1.04$cm^2$, AUCs were 0.75, 0.69, 0.80, respectively. In the last CT findings of 30 patients readmitted with EBV, logistic regression analysis showed that the diameter of LGV >5.90mm (OR=2.136, P=0.007), and the vascular area>1.04$cm^2$ (OR=2.338, P=0.013), which were independent risk factors for hemorrhage.
**Conclusion:** MSCTP can be used as an alternative to predict EVB in cirrhotic patients, the LGV diameter and the vascular area on MSCTP can be used as new effective indicators to predict the occurrence of EVB in patients with liver cirrhosis.

### *SS 13.5*

### Acute alcoholic hepatitis, towards a radiological diagnosis?

F. Grillet, P. Calame, J.P. Cervoni, E. Delabrousse; Besançon/FR

**Purpose:** To study the radiological signs of acute alcoholic hepatitis (AAH), and their regression.
**Material and methods:** For this monocentric observational study, we retrospectively established a list of 104 consecutive patients with a histological diagnosis of AAH, between January 2008 and June 2018. We included 57 patients who had undergone an injected cross-sectional imaging up to 30 days before the liver sampling. The radiological signs we analysed were based on the histological pattern of AAH: the presence of heterogeneous steatosis, hepatic perfusion disorders, and signs of liver dysmorphia. We evaluated the regression of radiological signs observed on the control cross-sectional imaging when performed. We created two control groups of patients with histologically proven cirrhosis (respectively, alcohol-induced without AAH and metabolic induced) and a prior cross-sectional imaging. We then paired patients between the AAH group and each control groups.
**Results:** In the 57 included patients, 54 (94.7%) had a heterogeneous steatosis, 39 (78%) hepatic perfusion disorders and 48 (84.1%) signs of liver dysmorphia. When analysing the paired patients, the association of those 3 signs showed a 100% specificity and positive predictive value towards the AAH diagnosis. For the 30 patients who underwent a follow-up imaging, a regression of both steatosis and perfusion disorders was observed in 21 of them (70%) and the liver's volume decreased by a mean 38%.
**Conclusion:** In our study, the association of heterogeneous steatosis, hepatic perfusion disorders and signs of liver dysmorphia was a specific radiological pattern for the diagnosis of acute alcoholic hepatitis.

### *SS 13.6*

### Predicting different stages of liver fibrosis with 2D shear-wave elastography: histopathological correlation study

M. Aksakal, S. Özhan Oktar, H. Şendur, G. Esendağlı Yılmaz, S. Özenirler, M. Cindoruk, K. Hızel, F.N. Baran Aksakal, C. Yücel; Ankara/TR

**Purpose:** In this study, we investigated the accuracy of 2D shear-wave elastography (2D SWE) in detecting liver fibrosis using histopathological analysis as the reference method.
**Material and methods:** Our single-center prospective study included 80 consecutive adult patients who had liver biopsy within 14 months of elastographic examination. A real-time SD SWE evaluation was performed using LOGIQ E9 system (GE Medical Systems, Wisconsin, USA). The median values of 10 valid liver stiffness measurements in kPa for each patient were obtained and were compared with the METAVIR scores obtained from the liver biopsy, using Kendall's rank correlation test. The diagnostic performance of real-time 2D SWE was assessed and cut-off values were set by ROC curve analysis.
**Results:** A significant correlation was found between liver stiffness kPa values and degree of liver fibrosis (Kendall's tau=0.56, p=0.0001). Our cut-off values for different stages of fibrosis were (F1-4); 5.92 kPa (sensitivity 75%, specificity 70%) and 6.68 kPa (sensitivity 95%, specificity 85%), 9.20 kPa (sensitivity 90%, specificity 96%) and 12.8 kPa (sensitivity 80%, specificity 96%), respectively.
**Conclusion:** Our findings suggest that 2D-SWE can be used for the assessment of liver fibrosis. It should be noted the best sensitivity value (%95) obtained in our study for identifying patients with no or minimum/mild fibrosis and those with severe fibrosis and cirrhosis (F0-1 versus F2-3-4). This is an important finding since patients with severe fibrosis need to be closely supervised. Studies on larger groups of biopsied patients are required to establish the most appropriate cut-off values for each particular stage of liver fibrosis.

### *SS 13.7*

*Withdrawn by the authors*

### *SS 13.8*

### Revisiting the role of US surveillance for HCC in chronic liver disease

J.H. Kim, S.J. Ahn, J.K. Han; Seoul/KR

**Purpose:** We investigated the role of US surveillance for HCC in chronic liver disease and analyzed the factors which affect US detection of HCC
**Material and methods:** Among 16986 patients with chronic liver disease who underwent surveillance US, 1901 patients who underwent both surveillance US and CT within 6 months were included in this study. We retrospective evaluated patient characteristics, US findings, and laboratory assessment. We also assessed the factor which affects the development of HCC within 2 years.
**Results:** 158 patients (8.3%) with US surveillance were confirmed with HCC. For 158 HCCs, US detected 81 HCC (51%). The sensitivity and specificity were 51.3% and 90.8%, respectively. Tumor size, echo texture, surface nodularity, and grade of fibrosis on US showed significantly different factors for the detection of HCC on surveillance US (p<0.005). In multivariate analysis, surface nodularity (OR, 0.314; p= .003) on US was the only significant factor. On follow-up of 1743 patients without HCC, HCC developed in 110 patients within 2 years (mean time: 13 months). According to multivariable analysis, numerous indeterminate nodules<1cm on US (OR, 2.15; p< .01), age (OR, 1.05; p< .01), ALT (OR, 1.01; p= .02), and platelet count (OR, 0.99; p< .01) were significantly related to the development of HCC.
**Conclusion:** US surveillance for HCC plays an important role in the detection of HCC in chronic liver disease. However, US findings including echo texture, surface nodularity, and grade of fibrosis affect the detection of HCC. Numerous indeterminate nodules<1cm on US increased the risk of development of HCC within 2 years.

### *SS 13.9*

### Resona 7 speed of sound index diagnostic performance in B-mode US using liver biopsy as 'Gold Standard' for non-alcoholic fatty liver disease and non-alcoholic steatohepatitis assessment

I. Gatos[1], P. Drazinos[1], S.D. Yarmenitis[2], E. Manessis[2], P.S. Zoumpoulis[1]; [1]Kifissia/GR, [2]Athens/GR

**Purpose:** Non-alcoholic fatty liver disease (NAFLD) may lead to non-alcoholic steatohepatitis (NASH) and, if not opposed, to cirrhosis and liver failure. NAFLD conventional diagnosis using B-mode US is performed by visual inspection of liver parenchyma compared to right kidney cortex brightness level. Recent studies have indicated differences in speed of sound (SoS) propagation on liver tissue and fat. Resona 7 US device provides the feature to measure optimum SoS for a small area on B-mode. The purpose of this study is to evaluate SSI feature for quantification of liver steatosis using liver biopsy (LB) as 'Gold Standard'.
**Material and methods:** 179 consecutive subjects (74 normal (S0) and 105 (S1-S3) NAFLD or NASH) with LB classified according to Kleiner score were included in the study. A B-mode ultrasound examination was performed on the right lobe of each patient using Resona 7 US device calculating SSI from a liver parenchyma area. Receiver operating characteristic (ROC) analysis was followed to establish an SSI threshold providing maximum differentiation accuracy for all steatosis grades.
**Results:** ROC analysis showed an AUC of 0.7996 for S≥S1, 0.8477 for S≥S2 and 0.8463 for S=S3. SSI best cutoff values for each class were 1522 m/s for S≥S1, 1516 m/s for S≥S2 and 1508 m/s for S=S3.
**Conclusion:** This study shows that SSI parameter of Resona 7 is correlated with steatosis grade and can be measured in an objective way. Also, it accurately differentiates patients with significant steatosis (S2) and severe steatosis (S3) but is less accurate in differentiating healthy subjects (S0).

### *SS 13.10*

### Liver surface nodularity quantification on preoperative CT: a predictive measurement of the operative risk after liver resection for resectable HCC

C. Hobeika, F. Cauchy, R. Sartoris, V. Vilgrain, O. Soubrane, M. Ronot; Clichy/FR

**Purpose:** To assess the ability of liver surface nodularity (LSN) quantification on CT for the anticipation of post-operative morbidity after liver resection for hepatocellular carcinoma (HCC).
**Material and methods:** This is a retrospective analysis of patients undergoing liver resection for HCC between 2012 and 2017. LSN was assessed at the surface of the left lobe on preoperative CT (<6 weeks before surgery) and defined as the mean of 10 measurements. Primary outcome was the association of LSN and severe (Dindo-Clavien 3-5) postoperative complications. Secondary outcomes included the association between LSN and severe postoperative complications in patients with and without severe (F3-F4) fibrosis, and between LSN and liver failure. ROC curve analysis was performed to determine LSN cut-off values. Risk factors for severe postoperative complications were identified with multivariate analysis.
**Results:** Among 239 patients undergoing resection for HCC, LSN measurements were invalid (<5 measurements) in 23 (10.9%) of the 210 patients eligible for LSN quantification. Mean LSN was 2.42±0.34. The AUROC of LSN for predicting major postoperative complications was 0.692 (p=0.001, cut-off 2.63). On multivariate analysis, LSN was significantly associated with the occurrence of major complications (HR: 5.23; 95%CI: 1.69-16.17; p=0.001). It was also associated with post-operative liver failure (AUROC: 0.653; p=0.014) and major complications in patients with (AUROC: 0.654, p=0.014) and without (AUROC: 0.698, p=0.012) severe fibrosis.
**Conclusion:** LSN measurement is feasible and reliable in the preoperative workup of patients with resectable HCC. It may represent a valuable tool in the anticipation of major postoperative complications in patients with and without severe fibrosis.

**11:00 - 12:30** **Room Amalfi**

*Scientific Session SS 14*

### Technical advances in abdominal imaging

### *SS 14.1*

### Prognostic value of preoperative diffusion-weighted MRI in colorectal peritoneal carcinomatosis patients using leading prediction models

M. Engbersen, I. Van't Sant, J. Velzing, D. Lambregts, H. Van Eden, R.G.H. Beets-Tan, A. Aalbers, N. Kok, M. Lahaye; Amsterdam/NL

**Purpose:** The purpose of the study is to compare the performance of diffusion-weighted (DW) MRI to predict complete cytoreductive surgery (CRS), disease-free survival and overall survival using 5 different prediction models for hyperthermic intraperitoneal chemotherapy (HIPEC) candidates with colorectal cancer.
**Material and methods:** Between February 2016 and October 2017, patients with colorectal peritoneal carcinomatosis considered for CRS/HIPEC who underwent DW-MRI for preoperative staging were included. DW-MRI images were evaluated, retrospectively, to determine the PCI. Relevant clinical parameters were obtained from patient files. Five scoring models were used; the Peritoneal Surface Disease Severity Score (PSDSS), Region Count, Simplified Peritoneal Cancer Index (SPCI), Peritoneal Cancer Index (PCI), and the Colorectal Peritoneal Metastases Prognostic Surgical Score (COMPASS). The performance of the prediction models was assessed by receiver operator characteristics (ROC) analysis and Cox-proportional analysis.
**Results:** Ninety-one patients were included. The mean age was 62.30 (±10.93) and 49% (45/92) of the patients were female. Fifty-three (58%) patients underwent successful CRS/HIPEC. For 38 patients, CRS/HIPEC was deemed infeasible, mostly due to extensive peritoneal carcinomatosis (23/38). ROC curve analysis for predicting a complete cytoreduction showed AUCs for PSDSS, region count, SPCI, PCI and COMPASS of 0.79, 0.81, 0.82, 0.85 and 0.86, respectively. PSDSS, region count, SPCI, PCI, and COMPASS showed significant hazard ratios for overall survival of 1.19, 1.69, 1.22, 1.12, and 1.02. None were found for disease-free survival.
**Conclusion:** DW-MRI seems to be able to select patients with colorectal peritoneal carcinomatosis eligible for CRS/HIPEC. All five predictive models are predictive of complete cytoreduction and overall survival, not of disease-free survival.

### SS 14.2

### Optimization of contrast medium volume in abdominal CT: a prospective comparison between fixed and lean body weight-adapted injection protocols

E. Rosati[1], N. Panvini[2], G. Moltoni[1], E. Lucertini[1], B. Bracci[1], D. Caruso[1], A. Laghi[1]; [1]Rome/IT, [2]Latina/IT

**Purpose:** To compare the performance of fixed and LBW-adapted contrast media (CM) administration, in terms of image quality and parenchymal enhancement.

**Material and methods:** Patients undergoing multiphasic 128-row abdominal CT were prospectively enrolled and randomly divided into two groups: patients in Group A received a fixed dose of 120 mL of CM while in Group B the amount of CM was computed according to the patient's lean body weight (LBW). Patient's characteristics, CM volume, contrast-to-noise ratio (CNR) and signal-to-noise ratio (SNR) of liver, pancreas, aorta and portal vein, and contrast enhancement index of liver (CEI-L) and pancreas (CEI-P) were compared between the two groups.

**Results:** The final population consisted of 40 patients (Group A: 20 patients, mean BMI 25.68±2.32 kg/m$^2$; group B: 20 patients, mean BMI 24.33±4.10 kg/m$^2$; all p≥0.380). Patients in group B received a significantly lower amount of CM (100.60±14.93 mL vs 120.00±0.00 mL; (p<0.001). Arterial liver and portal vein CNR were significantly higher in Group B (all p≤0.009). CEI-L showed no significant difference in both arterial and portal venous phases (all p≥0.050). Group A and Group B returned comparable liver CNR and SNR, pancreas CNR, aortic and portal vein CNR on portal venous phase and liver SNR, pancreatic CNR and SNR, aortic CNR and SNR, portal vein SNR, and CEI-P on arterial phase (all p≥0.922).

**Conclusion:** LBW-adapted CM administration for abdominal CT reduces the volume of injected CM without affecting image quality and parenchymal enhancement.

### SS 14.3

### Breath-hold compressed-sensing 3D MRCP compared to free-breathing 3D MRCP: prospective study of image quality and diagnostic performance in pancreatic disorders

I. Mannes[1], A. Dallongeville[2], N. Badat[2], H. Beaussier[2], G. Chatellier[2], M. Zins[2]; [1]Le Kremlin-Bicêtre/FR, [2]Paris/FR

**Purpose:** To compare 3T image quality and diagnostic performance in free-breathing standard and compressed-sensing (CS) 3D MRCP with breath-hold (BH) CS 3D MRCP, in patients with suspected pancreatic diseases.

**Material and methods:** Informed consent was obtained. We performed 57 MRI on 56 prospectively included patients (29 men, median age 59 years). Three protocols were randomly performed: CS-BH, CS and standard with free-breathing. Acquisition time was recorded. Quality image parameters (overall image quality, degree of artifacts, background suppression, bilio-pancreatic duct visualization) and diagnostic performances (anatomical variants, ductal abnormalities, cystic lesions) were evaluated on a 5-point scale by two radiologists blinded to the protocols.

**Results:** Acquisition time was 279s, 176s and 22s for standard, CS and CS-BH, respectively, reducing scan time by 37% and 93%. There was no significant difference in overall image quality, degree of artifacts and background suppression between standard and CS-BH. In the CS-BH group there were fewer non-diagnostic scans (3% in CS-BH versus 19% in standard and 21% in CS, p<0.05), higher quality scans (78% in CS-BH versus 66% in standard and 58% in CS, p<0.05) and less severe artifacts (2% in CS versus 18% in standard and 16% in CS, p<0.05). The main pancreatic duct was better visualized with CS-BH compared to standard (p=0.015) or CS (p<0.001). Diagnostic performance did not differ between all three protocols. There were fewer indeterminate exams in the CS-BH group.

**Conclusion:** CS-BH 3D MRCP is reliable, time-saving, with no degradation in image quality nor diagnostic performance and can be recommended at 3T.

### SS 14.4

### Evaluation of liver MR elastography analyzability criteria using a simulation method based on successively decreasing the size of the selected region-of-interest: a proof-of-concept study

M. Middleton[1], W. Henderson[1], C.K. Potu[1], T.I. Delgado[2], C. Chung[3], S. Djedjos[3], R.P. Myers[3], J. Chen[4], C.B. Sirlin[5]; [1]San Diego, CA/US, [2]Encinitas, CA/US, [3]Foster City, CA/US, [4]Rochester, MN/US, [5]La Jolla, CA/US

**Purpose:** In multi-institutional clinical trials, liver MR elastography (MRE) is considered analyzable if there are > 2,000 acceptable pixels across the four acquired slices, marginally analyzable if there are < 2,000 pixels but > 500-700 pixels, and not analyzable if there are < 500-700 pixels. However, little objective data supports these cutoffs. We performed a simulation study to analyze these cutoffs using data from a multi-center drug development clinical trial of adults with nonalcoholic steatohepatitis (NCT02854605).

**Material and methods:** Twenty-five cases were randomly selected from the aforementioned clinical trial. Liver stiffness values for every pixel in every subject were recorded. Average liver stiffness values were iteratively recalculated by randomly removing ten pixels at a time, and repeating each iteration 100 times. The range of liver stiffness values, across all 100 simulations, at 500, 700, and 2,000 pixels was captured, and means and standard deviations (SDs) of these ranges were calculated.

**Results:** Using all pixels, the mean(SD; range) liver stiffness value and ROI area were 3.44(1.54; 1.99-8.55) kPa and 4,272(2,276; 1,115-11,163) pixels, respectively. The mean(SD) of the range of simulated liver stiffness values for cutoffs of 500, 700, and 2,000 pixels were 0.19(0.14), 0.16(0.11), and 0.07(0.07) kPa, respectively.

**Conclusion:** The ranges of simulated liver stiffnesses for all three pixel cutoffs are small, and for most contexts of use are likely acceptable for drug development clinical trials, and probably also for clinical care. Hence, at a proof-of-concept level, subject to validation in larger cohorts, these MRE liver stiffness analyzability cutoffs are reasonable.

### SS 14.5

### Spleen MR elastography: what are the predictive factors of successful and usable data on a liver MR elastography acquisition?

P.-A. Lampson[1], R.H. Kim[2], O. Lucidarme[1], M. Wagner[1]; [1]Paris/FR, [2]Seoul/KR

**Purpose:** To determine if magnetic resonance elastography (MRE) data of the spleen are usable in case of liver MRE acquisition and to assess the determinants of non-usable spleen MRE data.

**Material and methods:** Eighty-six consecutive MRE examinations (gradient-recalled-echo), acquired on a 1.5T system, were retrospectively evaluated. Clinical data were recorded: chronic liver disease, gender, age, weight, BMI. Imaging data were recorded by one observer: degree of ascites, liver and spleen T2*, spleen largest diameter, body diameters (transverse and anteroposterior), stomach status (collapsed/half-filled/distended), liver MRE failure, liver MRE quality score based on the coverage on the confidence map. Spleen MRE non-usable data were defined as no pixel with confidence index higher than 95% and/or no apparent shear waves imaged and were assessed by 2 observers and a 3rd one in case of disagreement. Logistic regression analysis was performed to assess the link between spleen MRE success and potential predictive factors of failure.

**Results:** Spleen data were not usable in 20 cases (33%). On univariate analysis, higher BMI, larger transverse and anteroposterior body diameters, lower liver and spleen T2*, and lower liver MRE quality score were all significantly associated with non-usable spleen MRE data (P<0.016); while on multivariable analysis, only higher BMI, lower spleen T2* and lower liver MRE quality score were significantly associated with non-usable spleen MRE data (P<0.023).

**Conclusion:** Spleen MRE data on a liver acquisition are usable in 2/3 of the cases. Successful liver MRE acquisition, lower BMI and lower spleen iron content (higher T2*) are predictive factors of usable spleen MRE data.

### SS 14.6

### Probe pressure for shear wave elastography liver exams?

M. Byenfeldt; Umeå/SE

**Purpose:** Previous research has suggested that a large distance between the skin and the liver capsule (SCD), as is sometimes encountered in patients with obesity, has a negative impact on the reliability of ultrasound-based shear wave elastography (SWE) measurements. Although it has been postulated that increased intercostal probe pressure during SWE of the liver might lead to a false increase in the SWE result, this maneuver might be better suited to larger SCDs and might increase the number of technically successful measurements. This study examined the influence of increased intercostal probe pressure on SWE.
**Material and methods:** In this prospective clinical study, livers were measured with comb-push 2D technology. Maximum probe pressure was applied intercostally to reduce the SCD, in an effort to stabilize the SWE signal. Measurements obtained with normal pressure were compared to those obtained with maximum probe pressure.
**Results:** In this study, 2240 liver measurements were completed in 112 patients: 10 measurements with maximum probe pressure and 10 with normal pressure were performed per patient. Compared to normal pressure, maximum probe pressure significantly reduced the SCD (P<0.001) and significantly increased the number of technically successful measurements from 981 to 1098 (P<0.001). SWE results with normal and maximum probe pressures were 5.96 kPa (inter-quartile range: 2.41) and 5.45 kPa (inter-quartile range: 1.96), respectively (P <0.001).
**Conclusion:** In patients with obesity, large SCD poses a diagnostic challenge for ultrasound SWE. This clinical study showed that using maximum intercostal probe pressure could reduce the SCD without falsely increasing the SWE result.

### SS 14.7

*Withdrawn by the authors*

### SS 14.8

### Influence of injection rate in determining the development of artifact during acquisition of dynamic arterial phase in gadoxetic acid MRI studies

C. Maino, A. Pecorelli, C. Talei Franzesi, L. Riva, E.B. Orsini, S. Sironi, D. Ippolito; Monza/IT

**Purpose:** To assess whether modifications of injection rate of gadoxetic acid (Gd-EOB-DTPA) could influence the development of artifacts during arterial phase of liver MRI studies.
**Material and methods:** All the Gd-EOB-DTPA MRI studies performed between 2016 and 218 were retrospectively evaluated. Each MRI study was acquired on 1.5 T scanner (Achieva, Philips) with the following protocol: T1, T2, DWI, 3D-T1 fat-sat pre- and post (arterial, venous, delayed and hepatobiliary phase)-i.v. injection of CM (Gd-EOB-DTPA) with different flow injection rate (1 and 1.5 mL/sec). For each MRI, a radiologist recorded the presence of artifacts during different phases, respectively: all the examination, only during the arterial phase, only during portal venous phase, both arterial and portal venous phases.
**Results:** Of 748 MRI, 229 demonstrated the presence of artifacts in the entire examination. Of the remaining, 312 were obtained using a flow rate of 1 ml/s and 207 using a flow rate of 1.5 ml/s. We observed that 14 exams showed artifacts using a flow rate of 1 ml/min while 84 with a flow rate of 1.5 ml/s (4% vs 40%) only during the arterial phase, while 15 vs 21, respectively, only during portal venous phase (5% vs 10%) and 38 vs 52 in both arterial and portal venous phase (12% vs 25%). We found that the reduction of injection rate allows to reduce artifacts during arterial phase (p<0.0001).
**Conclusion:** The presence of motion artifacts of Gd-EOB-DTPA liver-MR studies can be related to flow injection rate, and this reduction may help in the better assessment and acquisition of the arterial phase.

### SS 14.9

### Role of dedicated subspecialized radiologists in multidisciplinary team recommendations for gastric neoplasms

S. Jang, S.H. Kim, H.-K. Yang, H.-J. Lee, S.-H. Kong, S.-A. Im, D.-Y. Oh, H.S. Chung, J.K. Han; Seoul/KR

**Purpose:** To identify discrepancies between radiologic reports generated by non-dedicated radiologists and a subspecialized dedicated GI radiologist for patients with gastric neoplasms and to assess if such discrepancies impact on patient management through a multidisciplinary team (MDT) approach.
**Material and methods:** Between August 2017 and August 2018, a dedicated GI radiologist prospectively reviewed all radiologic studies for 114 patients with biopsy-proven gastric neoplasms for MDT conference. The MDT reporting of radiologic studies were compared with the 'pre-MDT' formal report of the studies provided by various independent radiologists. The frequency and significance of any reporting variance were examined.
**Results:** In 16 MDT meetings, 114 patients were presented. Reasons for presentation were questions regarding treatment (44 %), radiologic findings (33%), pathology (24%), diagnosis (23 %), and diagnostic workup (4%). Of 114 patients, radiologic findings were changed in 31 (27%) cases through MDT conferences. Of them, 26 cases also involved changes in the treatment plan. In seven patients, unnecessary futile gastric surgery, laparoscopic staging biopsy, or chemotherapy was avoided. Conversely, eight patients could undergo curative surgery instead of palliative treatment. Among 83 cases without radiological reporting variance, 17 patients also had modification in diagnostic workup as a result of dedicated radiologist's second opinion. There was 93% (79/85) compliance with the conference management recommendations.
**Conclusion:** There is a considerable discrepancy for radiologic interpretations between non-dedicated and dedicated radiologists. A second-opinion provided by a subspecialized GI radiologist during MDT conferences improves an accuracy for staging and treatment plan recommendation and hugely affects patients' management strategy.

### SS 14.10

### Expert-reader validation of optimised super-resolution reconstruction for upper abdominal MRI

M.D. Chouhan, M. Ebner, P. Patel, D. Atkinson, L. Firmin, Z. Amin, P.D. Coppi, S. Ourselin, T. Vercauteren, S.A. Taylor; London/UK

**Purpose:** To validate optimised super-resolution reconstruction (SRR), a novel method for overcoming interslice motion artefact and suboptimal through-plane resolution on standard single-shot T2-weighted (SST2W) imaging, against standard axial and coronal SST2W imaging using expert readers.
**Material and methods:** Eight healthy normal volunteers underwent standard (axial and coronal) upper SST2W imaging, with additional sagittal and three oblique upper abdominal SST2W volumes. SRRs were generated using rigid outlier-robust motion correction, with optimisation around a pre-specified volume-of-interest centred around the common bile duct (CBD). Four radiologists, three with hepato-pancreatico-biliary subspecialisation and all with >7-year experience in abdominal imaging validated SRRs by direct comparison with standard SST2W imaging, scoring 9 anatomical sites for (i) preservation of information (not, poorly or clearly visualised, 0-2); (ii) SRR preference (worst, equal to or better than SST2W,-1 to +1); and (iii) presence of SRR artefacts (lots, minimal, none, or less than SST2W, 0-3).
**Results:** Bland-Altman analysis of summed preservation of information scores (maximum score 18) demonstrated a median bias of +1, with -2 and +3 limits-of-agreement. Median SRR preference averaged across 9 sites for each scan was 0.17, above and significantly different from SST2W equivalence score zero (p<0.05). Median presence of SRR artefacts averaged across 9 sites for each scan was 1.39 (between minimal and no artefact) and significantly different from no artefact score two (p<0.05).
**Conclusion:** SRRs preserve anatomical information, are slightly preferred but introduce some artefacts relative to standard SST2W imaging.

ESGAR
European Society
Gastrointestinal and Abdominal Radiology

### A

Aalbers A.: SS 14.1
Abdelaziz O.: SS 4.10
Abitbol C.: SS 3.3
Aboufirass Y.: SS 5.4
Agostini A.: SS 6.6, SS 11.3, SS 11.7
Agridag Ucpinar B.: SS 9.10
Ahn S.J.: SS 13.8
Aiello V.: SS 2.5, SS 12.6
Akay S.: SS 3.8
Aksakal M.: SS 13.6
Al-Nowfal A.: SS 4.1
Alberich-Bayarri A.: SS 7.2
Albertoni L.: SS 2.6
Aldegheri V.: SS 2.6
Aleksander-Markuszewska Z.: SS 10.1
Alfaro I.: SS 11.1
Allegra F.: SS 8.8
Amaddeo G.: SS 6.4
Ambrosetti M.C.: SS 8.7, SS 8.10, SS 10.10
Amin Z.: SS 14.10
An C.: SS 3.2
An J.: SS 4.6
Antonarelli M.: SS 11.3, SS 11.7
Apine I.: SS 11.4
Arcuri P.P.: SS 2.5, SS 12.6
Atar E.: SS 3.3
Atasoy K.: SS 9.9
Atkinson D.: SS 14.10
Aubé C.: SS 1.4
Auer T.A.: SS 12.7
Ayed A.: SS 12.10
Ayuso C.: SS 6.2
Azorin Vicente J.P.: SS 3.7, SS 11.5
Azoulay A.: SS 8.6

### B

Ba-Ssalamah A.: SS 12.2, SS 12.8
Badat N.: SS 14.3
Badia S.: SS 11.9
Bae J.: SS 1.2
Bainbridge A.: SS 1.9
Bali M.A.: SS 5.2, SS 11.9
Balyemez U.: SS 3.8
Baran Aksakal F.N.: SS 13.6
Baranes L.: SS 6.4
Barcellitti J.: SS 12.10
Bargellini I.: SS 5.3
Barison A.: SS 2.6
Bashir M.R.: SS 6.1
Bastati-Huber N.: SS 12.2, SS 12.8
Batakis D.N.: SS 12.1
Batista Domenech A.: SS 3.7, SS 11.5
Beaussier H.: SS 14.3
Beckers R.C.J.: SS 7.1
Beer A.: SS 12.2
Beer L.: SS 12.8
Beets-Tan R.G.H.: SS 5.7, SS 7.1, SS 12.9, SS 14.1
Beleù A.: SS 3.10, SS 8.10, SS 10.8, SS 10.9
Bellini D.: SS 2.1, SS 9.2, SS 9.5, SS 11.9
Bellini N.: SS 10.9
Bellomi M.: SS 10.1
Belmonte E.: SS 6.2
Benseghir T.: SS 5.10
Bertotto I.: SS 2.9
Bertucci C.: SS 2.5, SS 12.6
Berzigotti A.: SS 1.3, SS 13.3
Bläker H.: SS 12.7
Boehringer A.S.: SS 12.1
Bonatti M.: SS 9.4, SS 10.10
Boni G.: SS 5.3
Borgheresi A.: SS 6.6, SS 11.3, SS 11.7
Borhani A.A.: SS 1.7, SS 6.8, SS 13.1
Boursier J.: SS 1.4
Bozzi E.: SS 5.3
Bracci B.: SS 14.2
Brancatelli G.: SS 8.8
Braren R.: SS 7.4
Brook A.: SS 8.9
Bruix J.: SS 6.2
Brunsing R.L.: SS 7.9
Byenfeldt M.: SS 14.6

### C

Calame P.: SS 4.3, SS 13.5
Caldéraro J.: SS 6.4
Campi C.: SS 2.6
Campioni D.: SS 11.3
Camurcuoglu E.: SS 9.10
Cannella R.: SS 1.7, SS 6.1, SS 6.8, SS 8.8, SS 13.1
Caparroz C.: SS 6.2
Capozzi N.: SS 11.1
Cappello G.: SS 5.8
Carbone I.: SS 9.2, SS 9.5, SS 11.9
Cardano G.: SS 8.3
Cardobi N.: SS 3.10, SS 8.4, SS 10.8, SS 10.9
Caruso D.: SS 2.1, SS 2.3, SS 2.7, SS 7.8, SS 14.2
Castera L.: SS 1.1, SS 5.1
Castiglione D.G.: SS 8.8
Catania M.: SS 8.10
Cauchy F.: SS 13.2, SS 13.10
Cela F.: SS 11.7
Cervoni J.P.: SS 13.5
Chandramohan A.: SS 2.2
Chang H.C.: SS 7.5
Chao S.-L.: SS 5.2
Chapman S.J.: SS 11.6
Chatellier G.: SS 5.1, SS 14.3
Chen J.: SS 14.4
Chen S.M.: SS 6.9
Chen W.: SS 6.10
Chincarini M.: SS 8.7
Chiu W.H.K.: SS 5.6, SS 7.5
Cho E.J.: SS 1.2
Choi B.I.: SS 1.2
Choi S.-Y.: SS 3.4, SS 8.2, SS 8.5
Choudhoury K.R.: SS 6.1
Chouhan M.D.: SS 1.9, SS 14.10
Christe A.: SS 1.3, SS 13.3
Chung C.: SS 12.1, SS 14.4
Chung H.S.: SS 14.9
Chung W.-S.: SS 4.6
Cindoruk M.: SS 13.6
Cioni R.: SS 5.3
Cobelli R.: SS 4.7
Coi F.: SS 9.1
Conte G.: SS 6.6
Cook G.: SS 3.1
Coppi P.D.: SS 14.10
Corthouts B.: SS 3.9
Crimì F.: SS 2.6
Cros J.: SS 8.6
Cucci E.: SS 10.5
Cybulski A.: SS 10.4

### D

D'Arrigo B.: SS 7.8
D'Onofrio M.: SS 3.10, SS 8.4, SS 8.10, SS 10.8, SS 10.9
Dalla Bona E.: SS 6.6
Dallongeville A.: SS 14.3
Darnell A.: SS 6.2
Dasyman N.: SS 13.1
Dave M.: SS 11.2
De Boer M.: SS 5.7
De Haas R.J.: SS 2.4
De Jong K.P.: SS 2.4
De Lucia S.: SS 11.8
De Maria L.: SS 7.8
De Robertis R.: SS 3.10, SS 8.4, SS 8.10, SS 10.8, SS 10.9
De Santis D.: SS 2.1, SS 2.7
De Sousa Mendes M.: SS 5.9
De Vos N.: SS 13.2
De With P.H.N.: SS 8.1
Debry J.-B.: SS 5.10

Decicco C.: SS 8.9
Delabrousse E.: SS 4.3, SS 13.5
Delgado T.I.: SS 7.9, SS 12.1, SS 14.4
Delmastro E.: SS 2.9
Demir A.A.: SS 10.3
Denecke T.: SS 5.9, SS 12.7
Deng Y.: SS 6.9
Descovich-Garces A.: SS 8.9
Devito A.: SS 4.8
Di Chio A.: SS 7.7
Díaz-González Á.: SS 6.2
Dierckx R.A.J.O.: SS 2.4
Dieudonne A.: SS 5.1
Dijkhoff R.: SS 7.1
Dimarco M.: SS 8.8
Dioguardi Burgio M.: SS 1.1, SS 5.10, SS 12.5
Djabbari M.: SS 6.4
Djedjos S.: SS 12.1, 14.4
Dobroschke J.: SS 4.4
Drago S.G.: SS 9.8
Drazinos P.: SS 13.9
Dregely I.: SS 3.1
Dromain C.: SS 12.4
Dubois M.: SS 12.10
Duran R.: SS 12.4
Durmaz S.: SS 9.10

### E

Eapen A.: SS 2.2
Ebner L.: SS 1.3, SS 13.3
Ebner M.: SS 14.10
El Addouli H.: SS 3.9
Elmer M.C.: SS 12.8
Emadeldin S.: SS 4.10
Engbersen M.: SS 14.1
Ersen M.: SS 3.8
Erturk S.M.: SS 9.10
Esendağlı Yılmaz G.: SS 13.6
Evans R.: SS 9.6

### F

Faccini R.: SS 2.3
Faccioli N.: SS 9.4
Faletti R.: SS 7.7
Fazio N.: SS 10.1
Fehrenbach U.: SS 5.9, SS 12.7
Feier D.: SS 12.2
Fernandez-Clotet A.: SS 11.1
Ferrari R.: SS 2.3
Ferri M.: SS 9.1
Festa A.: SS 11.8
Fighera A.: SS 8.3
Fiorentini G.: SS 6.8
Firmin L.: SS 14.10
Flamen P.: SS 5.4
Fodero G.: SS 2.5, SS 12.6
Fonio P.: SS 7.7
Fontanella G.: SS 11.8
Forner A.: SS 6.2
Forte V.: SS 7.8
Fourquet A.: SS 12.10
Fowler K.: SS 7.9
Fragner R.: SS 12.2
Frulloni L.: SS 8.3
Funicelli L.: SS 10.1
Furlan A.: SS 1.7, SS 6.1, SS 6.8, SS 13.1

### G

Gabriele P.: SS 2.9
Gaillard F.: SS 7.6
Galati M.C.: SS 12.6
Galletto Pregliasco A.: SS 6.4
Gao J.: SS 2.10
Garcia Alba C.: SS 5.10
Garcia-Junco M.: SS 7.2
Garin E.: SS 5.1
Garteiser P.: SS 1.1
Gatos I.: SS 13.9
Gatti M.: SS 7.7
Gavrielli S.: SS 3.3
Geisel D.: SS 5.9, SS 12.7
Gemini S.: SS 11.3
Ghorra C.: SS 12.3
Giandola T.: SS 4.8
Giannetta V.: SS 10.1
Giannini V.: SS 2.9, SS 5.8
Giannotti G.: SS 10.8, SS 10.9
Giaretta A.: SS 3.10
Gibson R.N.: SS 7.6
Giovagnoni A.: SS 6.6, SS 11.3, SS 11.7
Glickman J.: SS 8.9
Goh V.: SS 3.1
Gómez Muñoz F.: SS 5.7
Gonzalez N.S.: SS 4.4
Grazzini G.: SS 9.3
Grecchi A.: SS 3.10, SS 8.10, SS 10.9
Grieser C.: SS 12.7
Grillet F.: SS 4.3, SS 13.5
Gräni C.: SS 1.3
Guarasci F.: SS 7.7
Guarnera A.: SS 2.7
Guido G.: SS 7.8
Gül S.: SS 5.9
Gulani V.: SS 11.2
Gullì C.: SS 10.5

### H

Hall-Craggs M.: SS 1.9
Halligan S.: SS 3.6, SS 9.6
Han J.K.: SS 1.2, SS 6.7, SS 13.8, SS 14.9
Harada T.L.: SS 10.6
Hasenstab K.A.: SS 7.9, SS 12.1
Hayoz R.H.: SS 12.4
He B.J.: SS 6.9
Heeres B.: SS 12.9
Heid I.: SS 7.4
Helliwell J.A.: SS 11.6
Henderson W.: SS 12.1, SS 14.4
Hermann A.-L.: SS 5.1
Hermans J.J.: SS 8.1
Heverhagen J.: SS 1.3, SS 13.3
Hezode C.: SS 6.4
Higaki A.: SS 7.9
Hobeika C.: SS 13.10
Houwers J.B.: SS 7.1, SS 12.9
Hrycyk J.: SS 1.3, SS 13.3
Hsiao A.: SS 7.9
Huang T.Y.: SS 7.5
Huber A.: SS 1.3, SS 13.3
Hudson S.R.: SS 4.1
Hui E.S.: SS 7.5
Hwang J.A.: SS 8.2, SS 8.5
Hızel K.: SS 13.6

### I

Iannicelli E.: SS 7.3, SS 9.1
Ichikawa S.: SS 7.9
Ilhan H.: SS 5.5
Im S.-A.: SS 14.9
Imani F.: SS 5.7
Ingenerf M.K.: SS 5.5
Ippolito D.: SS 4.8, SS 9.8, SS 14.8
Ith M.: SS 1.3
Ivan C.: SS 4.1

### J

Jang S.: SS 14.9
Janse M.H.A.: SS 8.1
Jayne D.G.: SS 11.6
Jeljeli S.: SS 3.1
Jesudason M.R.: SS 2.2
Jiang H.: SS 6.5
Jiang Y.: SS 11.2
Jimenez-Pastor A.: SS 7.2
Jo S.J.: SS 2.8
Joo I.: SS 6.7
Jouve De Guibert P.-H.: SS 12.10

### K

Kaiser L.: SS 5.5
Kaissis G.: SS 7.4
Kang H.-J.: SS 6.7
Karim H.: SS 5.5
Katz J.: SS 11.2
Kazci Z.: SS 9.10
Keles B.: SS 9.10
Kharrat R.: SS 6.4
Kim H.: SS 1.2
Kim J.E.: SS 4.2
Kim J.H.: SS 3.4, SS 6.7, SS 8.5, SS 13.8
Kim J.Y.: SS 4.6
Kim M.-J.: SS 3.2, SS 6.3
Kim R.H.: SS 14.5
Kim S.H.: SS 2.8, SS 4.2, SS 14.9
Kim Y.: SS 4.5
Klompenhouwer E.: SS 5.7
Knebel J.-F.: SS 12.4
Kok N.: SS 14.1
Kong S.-H.: SS 14.9
Kuang S.C.: SS 6.9
Kwee T.C.: SS 2.4

### L

Labruna R.: SS 10.1
Lacognata C.: SS 2.6
Laganà D.: SS 2.5, SS 12.6
Laghi A.: SS 2.1, SS 2.3, SS 2.7, SS 4.9, SS 7.3, SS 7.8, SS 9.1, SS 9.2, SS 9.5, SS 11.9, SS 14.2
Lahaye M.: SS 14.1
Lambregts D.: SS 5.7, SS 7.1, SS 12.9, SS 14.1
Lampson P.-A.: SS 12.10, SS 14.5
Landolfi F.: SS 7.3, SS 7.8, SS 9.1
Laniado M.: SS 4.4
Laurino F.: SS 4.9
Lazareth M.: SS 1.10
Le Deunf L.: SS 12.10
Lebigot J.: SS 1.4
Lebtahi R.: SS 5.1
Lee D.H.: SS 1.2, SS 1.8
Lee E.S.: SS 1.8
Lee E.Y.P.: SS 5.6
Lee H.-J.: SS 14.9
Lee H.J.: SS 4.6
Lee J.: SS 1.2, SS 4.5
Lee J.E.: SS 3.4, SS 8.2, SS 8.5
Lee J.Y.: SS 1.2
Lee S.: SS 6.3, SS 8.2
Lee Y.: SS 4.2
Lee Y.B.: SS 1.2
Lemort M.: SS 5.2, SS 5.4
Li J.: SS 6.10
Litjens G.: SS 8.1
Lo W.-C.: SS 11.2
Lohöfer F.: SS 7.4
Lombardo F.: SS 9.4, SS 10.10
Londhe D.: SS 2.2
Lopez-Gonzalez R.: SS 7.2
Lovell T.: SS 7.6
Lucertini E.: SS 2.7, SS 14.2
Luciani A.: SS 6.4, SS 12.5
Lucidarme O.: SS 12.10, SS 14.5

### M

Maas M.: SS 5.7, SS 7.1, SS 12.9
Maggs D.: SS 1.9
Maino C.: SS 4.8, SS 9.8, SS 14.8
Malakhia A.: SS 4.3
Mallett S.: SS 3.6
Mamidiapalli A.: SS 12.1
Mancinelli M.: SS 11.8
Mancini Terracciano C.: SS 2.3
Mandorfer M.: SS 12.8
Manessis E.: SS 13.9
Manganiello C.A.T.: SS 11.8
Mannes I.: SS 14.3
Manning M.: SS 8.9
Mansueto G.: SS 8.3, SS 8.7, SS 9.4, SS 10.4, SS 10.10
Mantellini P.: SS 9.3
Marin D.: SS 6.1
Marrazzo A.: SS 10.5
Marsoni S.: SS 5.8
Marti-Aguado D.: SS 7.2
Marti-Bonmati L.: SS 3.7, SS 7.2, SS 11.5
Martini I.: SS 7.3, SS 9.1
Marx C.: SS 1.3, SS 13.3
Masamunt M.: SS 11.1
Mascalchi M.: SS 9.3
Mazzaro A.: SS 8.7
Mazzei E.: SS 2.5
Mazzetti S.: SS 2.9, SS 5.8
McCusker M.W.: SS 7.6
Mebis W.: SS 3.9
Meek D.: SS 5.7, SS 7.1
Menghini R.: SS 10.4
Menys A.: SS 11.6
Meyer M.: SS 6.1
Middleton M.: SS 12.1, SS 14.4
Miles A.: SS 9.6
Min J.H.: SS 8.2
Minervini M.I.: SS 1.7
Missere M.: SS 10.5
Mitchell D.: SS 7.6
Mittal R.: SS 2.2
Moltoni G.: SS 14.2
Montemezzi S.: SS 8.4
Moon J.Y.: SS 4.5, SS 8.2
Moret A.: SS 1.4
Morris S.: SS 9.6
Mortele K.J.: SS 8.9
Mosca P.: SS 11.3
Moser A.J.: SS 8.9
Moura Cunha G.: SS 7.9, SS 12.1
Mule S.: SS 6.4, SS 12.5
Müller T.: SS 12.7
Murelli V.: SS 4.7
Murshid K.J.A.: SS 2.4
Myers R.P.: SS 12.1, SS 14.4

### N

Nagakawa Y.: SS 10.6
Nagao T.: SS 10.6
Negrelli R.: SS 8.4
Neiman V.: SS 3.3
Neji R.: SS 3.1
Nicolay S.: SS 3.9
Nicolini D.: SS 6.6
Nivolli A.: SS 1.10

### O

Obmann V.: SS 1.3, SS 11.2, SS 13.3
Oh D.-Y.: SS 14.9
Op De Beeck B.: SS 3.9
Ordás I.: SS 11.1
Orgera G.: SS 4.9
Orsini E.B.: SS 14.8
Osman A.: SS 4.1
Osti M.F.: SS 9.1
Ottaviani L.: SS 11.3, SS 11.7
Ourselin S.: SS 14.10
Oven Ustaalioglu B.B.: SS 10.3
Özenirler S.: SS 13.6
Özhan Oktar S.: SS 13.6
Ozmen E.: SS 9.10

### P

Paisant A.: SS 1.4
Palumbo A.A.: SS 4.7
Panda A.: SS 11.2
Panes J.: SS 11.1
Panvini N.: SS 2.1, SS 7.8, SS 14.2
Panzuto F.: SS 7.3
Paradis V.: SS 1.1
Paramatti R.: SS 2.3
Park H.J.: SS 8.2
Park K.S.: SS 4.5

Park S.J.: SS 2.8
Park Y.N.: SS 3.2
Pashapoor S.: SS 9.9, SS 11.10
Patel P.: SS 14.10
Pecorelli A.: SS 4.8, SS 9.8, SS 14.8
Pedrazzi G.: SS 4.7
Pellegrino S.: SS 8.8
Pennings J.P.: SS 2.4
Pereira H.: SS 5.1
Perez Girbes A.: SS 3.7, SS 11.5
Perrone O.: SS 5.3
Phou V.: SS 12.10
Picchia S.: SS 11.9
Pigneur F.: SS 6.4, SS 12.5
Pilozzi E.: SS 9.1
Pisani A.: SS 11.3
Pisano A.: SS 4.9
Pitura R.: SS 11.4
Plodeck V.: SS 4.4
Polici M.: SS 7.3
Pommier R.: SS 12.3
Pötter-Lang S.: SS 12.2, SS 12.8
Potu C.K.: SS 14.4
Pratschke J.: SS 5.9
Prenois A.: SS 12.10
Prezzi D.: SS 3.1
Prokop M.: SS 8.1
Pucciarelli F.: SS 2.7
Puhl G.: SS 5.9
Purcell Y.: SS 12.5

Q

Quaia E.: SS 2.6
Quinn L.: SS 3.6

R

Rachakonda V.: SS 1.7
Ram T.S.: SS 2.2
Raschzok N.: SS 12.7
Rautou P.-E.: SS 1.1, SS 1.10, SS 13.2
Regge D.: SS 2.9, SS 5.8
Regnault H.: SS 6.4
Reiberger T.: SS 12.8
Reig M.E.: SS 6.2
Reizine E.: SS 1.1, SS 12.5
Rengo M.: SS 2.1, SS 2.3, SS 9.2, SS 9.5, SS 11.9
Restaino G.: SS 10.5
Rhee H.: SS 3.2
Ribeiro Castro Roriz D.J.: SS 3.7
Ricke J.: SS 5.5
Rimola J.: SS 6.2, SS 11.1
Rinzivillo M.: SS 7.3
Ripoll E.: SS 6.2
Riva L.: SS 4.8, SS 14.8
Rizzo G.: SS 3.10, SS 8.10, SS 10.8, SS 10.9
Roccia S.: SS 2.5, SS 12.6
Roche V.: SS 5.10
Romagnoli R.: SS 7.7
Romano M.: SS 6.6
Rong D.L.: SS 6.9
Ronot M.: SS 1.1, SS 1.10, SS 5.1, SS 5.10, SS 8.6, SS 12.3, SS 12.5, SS 13.2, SS 13.10
Ropella-Panagis K.: SS 11.2
Rosati E.: SS 14.2
Rossi M.: SS 4.9
Rudenko P.: SS 3.7, SS 11.5
Rummeny E.J.: SS 7.4
Russo U.: SS 4.7

S

Saifi A.: SS 5.2
Saito K.: SS 10.6
Sakai N.: SS 1.9
Sali L.: SS 9.3
Sallustio G.: SS 10.5
Sanchez M.: SS 5.1
Santi E.: SS 9.4
Sarno A.: SS 3.10
Sartoris R.: SS 1.10, SS 13.2, SS 13.10
Scalise P.: SS 5.3
Schawkat K.: SS 8.9
Schlein A.: SS 7.9
Schmelzle M.: SS 12.7
Schmid-Tannwald C.: SS 5.5
Schwartzman A.: SS 7.9
Seah J.C.Y.: SS 7.6
Seehofer D.: SS 12.7
Şendur H.: SS 13.6
Seyfried N.: SS 11.2
Shen C.: SS 6.8
Sibert A.: SS 5.10
Siddiqui U.: SS 2.2
Sikora A.K.: SS 2.5, SS 12.6
Singer T.: SS 8.9
Singh A.: SS 2.2
Sinh P.: SS 11.2
Sirlin C.B.: SS 7.9, SS 12.1, SS 14.4
Sironi S.: SS 4.8, SS 9.8, SS 14.8
Siveke J.: SS 7.4
Snoeckx A.: SS 3.9
Song B.: SS 1.6, SS 6.5, SS 13.4
Soubrane O.: SS 13.10
Soydan L.: SS 10.3
Spinhoven M.J.: SS 3.9
Spolverato G.: SS 2.6
Staal F.: SS 5.7, SS 12.9
Stajgis P.: SS 11.5
Steffen I.: SS 5.9
Stephenson J.A.: SS 4.1
Stern J.: SS 5.9
Stirling J.: SS 3.1
Stramare R.: SS 2.6

T

Taghavi M.: SS 5.7, SS 7.1
Tagliamonte M.: SS 9.4
Talei Franzesi C.: SS 4.8, SS 9.8, SS 14.8
Tamir S.: SS 3.3
Tandoi F.: SS 7.7
Tang J.S.N.: SS 7.6
Tasar M.: SS 3.8
Taylor S.A.: SS 1.9, SS 3.6, SS 9.6, SS 14.10
Tedesco G.: SS 10.8
Terilli F.: SS 11.7
Terraz S.: SS 12.3
Testa I.: SS 3.10, SS 10.9
Tiberia F.: SS 9.2, SS 9.5
Tipaldi M.A.: SS 4.9
Tolan D.: SS 11.6
Torregrosa Andrés A.: SS 3.7, SS 11.5
Trebeschi S.: SS 7.1
Trionfera G.: SS 9.2, SS 9.5
Tse D.: SS 5.6
Tsung A.: SS 6.8

U

Urkan M.: SS 3.8

V

Valletta R.: SS 9.4, SS 10.4, SS 10.10
Van Beers B.: SS 1.1
Van Der Heide U.: SS 7.1
Van Dorth D.: SS 12.9
Van Eden H.: SS 14.1
Van Hoyweghen A.: SS 3.9
Van Lunenburg J.: SS 5.6
Van't Sant I.: SS 14.1
Vanzulli A.: SS 5.8
Vardhanabhuti V.: SS 5.6
Velzing J.: SS 14.1
Ventura L.: SS 9.3
Vercauteren T.: SS 14.10
Verhoef C.: SS 7.1
Verma R.: SS 4.1
Vernuccio F.: SS 6.1, SS 8.8
Vicini S.: SS 9.2, SS 9.5
Vietti Violi N.: SS 12.4

Vilgrain V.: SS 1.1, SS 1.10, SS 5.1, SS 5.10, SS 8.6, SS 12.3, SS 12.5, SS 13.2, SS 13.10
Villanacci R.: SS 11.8
Vivarelli M.: SS 6.6
Voena C.: SS 2.3
Vouche M.: SS 5.2, SS 5.4
Vullierme M.-P.: SS 8.6

### W

Wagner M.: SS 12.10, SS 14.5
Wan S.: SS 13.4
Wang J.: SS 6.9
Wang K.: SS 7.9, SS 12.1
Wang W.: SS 7.6
Wang X.: SS 2.10
Wei Y.: SS 1.6
Weichert W.: SS 7.4
Wilson G.C.: SS 6.8
Wright K.: SS 11.2
Wulf M.: SS 4.4

### X

Xie S.D.: SS 6.9
Xu J.: SS 10.2

### Y

Yamaguchi H.: SS 10.6
Yang F.: SS 6.10
Yang H.: SS 6.9
Yang H.-K.: SS 14.9
Yang W.: SS 6.10
Yarmenitis S.D.: SS 13.9
Ye Z.: SS 1.6
Yoon J.-H.: SS 4.2
Yosef L.: SS 3.3
Yuan H.: SS 5.6
Yücel C.: SS 13.6
Yunaiyama D.: SS 10.6

### Z

Zamboni G.A.: SS 8.3, SS 8.7, SS 10.4, SS 10.10
Zappa M.: SS 9.3
Zer A.: SS 3.3
Zerunian M.: SS 2.1, SS 2.3, SS 2.7, SS 7.3
Zhang Y.: SS 6.9
Zhu F.: SS 6.10
Zhu J.: SS 2.10
Ziegelmayer S.: SS 7.4
Zinger S.: SS 8.1
Zins M.: SS 14.3
Zoumpoulis P.S.: SS 13.9
Zucchetta P.: SS 2.6

The ESGAR 2019 Book of Abstracts is a supplement to Insights into Imaging and published under the Creative Commons Attribution License 4.0 (CC BY 4.0)
